# Genome analysis and phylogenetic characterization of two deformed wing virus strains from *Apis cerana* in Vietnam

**DOI:** 10.7717/peerj.9911

**Published:** 2020-09-21

**Authors:** Ha T. Thu, Nguyen T.K. Lien, Pham T. Lanh, Bui T.T. Duong, Nguyen T. Hoa, Man H. Phuoc, Pham H. Thai, Dong Van Quyen

**Affiliations:** 1 Laboratory of Molecular Microbiology, Institute of Biotechnology, Vietnam Academy of Science and Technology, Hanoi, Vietnam; 2 Laboratory of Functional Genomics, Institute of Genome Research, Vietnam Academy of Science and Technology, Hanoi, Vietnam; 3 Research Center for Tropical Bees and Beekeeping, Vietnam National University of Agriculture, Hanoi, Vietnam; 4 University of Science and Technology of Hanoi, Vietnam Academy of Science and Technology, Hanoi, Vietnam

**Keywords:** *Apis cerana*, Complete genome sequence, Deformed wing virus, Honeybee, Iflavirus

## Abstract

**Background:**

Deformed wing virus (DWV) is a virulent virus that causes honeybee disease. DWV can exist as a latent infection in honeybees, outbreak into epidemics, and cause serious damage to beekeeping cross the world, including Vietnam.

**Methods:**

The two DWV strains circulating in Vietnamese honeybee, *Apis cerana,* were first isolated from adult honeybees in North Vietnam (DWV-NVN) and South Vietnam (DWV-SVN). Their complete nucleotide sequences were determined, aligned, and compared with other DWV strains*.*

**Results:**

The two Vietnamese DWV strains comprised 10,113 bp and contained a large single open reading frame (ORF) of 2,893 amino acids, initiating at nucleotide 1,130 and terminating at nucleotide 9,812. Multiple nucleotide sequence alignment between these two DWV-VN strains and DWV strains in *A. mellifera* was performed. The DWV-VN strains showed a low genetic identity (from 91.4% to 92.0%) with almost of these strains, but lower identities (89.2% and 89.4%) with UK2 and (89.6%) with the China2 strain. Low identities (91.7% and 91.9%) were also observed between the China3 strain (in *A. cerana*) and the DWV-VN strains, respectively. The deduced amino acid sequence alignment showed high genetic similarities (97.0%–97.9%) when the USA1, Chile, Italy1, France, UK1, UK2, Japan, Korea2, China1, China2 and China3 strains were compared to the DWV-VN strains. This ratio was 96.7% and 96.8% when the Korea1 strain was compared to the DWV-SVN and DWV-NVN strains, respectively. Numerous amino acid substitutions were identified in the L, VP3, and RdRp sequences. Notably, we observed six substitutions positioned at amino acids 27 (E > I), 98 (S > T), 120 (A > V), 153 (M > T), 170 (D > F), and 174 (Y > F) in the L protein, two amino acid changes at positions 980 (S > A) and 1032 (E > T) in VP3, and one amino acid change at position 2627 (R > C) unique to the DWV-VN strains. Phylogenetic analysis based on complete genome sequences, RdRp sequences and Simplot analysis indicated that there was a significant difference between DWV-VN strains in *A. cerana* and DWV strains in *A. mellifera*. The results suggested that the genetic variations of the DWV-VN strains in *A. cerana* help them to adapt geographical conditions and may lead to change the viral pathogenicity of DWV-VN strains.

## Introduction

Since it first isolated from honeybees in Japan in the 1980s, DWV has spread all over the world (Allen & Ball, 1996; Calderon et al., 2003; Ellis & Munn, 2005; Antunez et al., 2006; Berenyi et al., 2007). It is found at every stages of development of honeybee (Chen, Higgins & Feldlaufer, 2005; Fievet et al., 2006; De Miranda & Fries, 2008). Moreover, DWV can exist in colonies with asymptomatic infections or benign symptoms. A line of evidence showed that DWV is the cause of colony losses and was most strongly linked to colony collapse (Chen, Higgins & Feldlaufer, 2005; Highfield et al., 2010; Genersch et al., 2010; Pohorecka et al., 2011; Dainat et al., 2012; Martin, Ball & Carreck, 2013).

DWV is usually transmitted through trophallaxis from adult honeybees or from the queens to progeny. In colonies, DWV may be re-activated when conditions are favorable (Chen et al., 2006; Yue, Gisder & Genersch, 2007; De Miranda & Fries, 2008; Mockel, Gisder & Genersch, 2011). DWV is considered to have a serious impact to morphological transformation from pupae to adult honeybees (Yang & Cox-Foster, 2007). The DWV infected honeybees have typical symptoms with the wings is deformed, the belly is short, flatulence and paralysis (Bailey & Ball, 1991), and their lifespan is significantly shortened (Yang & Cox-Foster, 2007). As a consequence, the number of worker honeybees decreases, leading to colony collapse (Berenyi et al., 2006).

Due to their huge effects on honeybee industry, many studies of complete genome of DWV have been carried out (Lanzi et al., 2006; Ongus et al., 2004; De Miranda & Genersch, 2010; Barriga et al., 2012; Reddy et al., 2013; Fei et al., 2019) in order to understand the virus evolution and how they can adapt to different hosts and geographic conditions. DWV has been classified into the *Iflavirus* genus based on the structure of its genome (Lanzi et al., 2006; Chen et al., in press). It is a RNA positive-sense with structural proteins at the N-terminal section and nonstructural proteins at the C-terminal section (Lanzi et al., 2006; Ongus et al., 2004). The N-terminal section consists of a leader polypeptide (L protein) and proteins VP2, VP1, and VP3. The C-terminal section contains of the RNA helicase, the putative VPG (genome-linked viral protein), and the RNA-dependent RNA polymerase (RdRp) (Lanzi et al., 2006; De Miranda & Genersch, 2010). However, almost all of the DWV published strains isolated from *A. mellifera* and DWV genome sequences are highly conserved (Berenyi et al., 2007). Research on the DWV genome sequence in *A. cerana* is very limited with only one publication of Fei et al. (2019). Therefore, understanding the genetic variations of DWV strains in *A. cerana* will be important to protect honeybees against the disease.

In the present study, the complete sequence of DWV isolated in *A. cerana* from different regions in Vietnam were determined and compared with those of DWV strains from other countries. The phylogenetic relationship and similarity among these DWV strains were analyzed to get new insights into the evolutionary origins of the DWV strains circulating and infecting *A. cerana* in Vietnam.

## Materials and Methods

### Sample collection and cDNA synthesis

The *A. cerana* adults were randomly collected from healthy hives / colonies in Hanoi (North) and Can Tho (South) of Vietnam. The owner of the honeybees permitted sampling and research of their hives located in Hanoi and Can Tho province, Vietnam. RNA extraction and cDNA synthesis were followed by Thu et al. (2016). cDNAs were synthesized using random hexamer primers of Maxima Reverse Transcriptase kit (Thermo Fisher Scientific Inc, Watham, MA, USA).

The DNA-positive samples were found by RT-PCR using a primer pair DWV F-5′-CGATTTATGCCTTCCATAGCG-3′ and DWV R-5′-ACTAAAATTAGGACGCATTACC-3′ in PCR conditions: 95 °C for 5 min at first, after that 40 cycles of 95 °C for 20 s, 55 °C for 30 s, 72 °C for 1 min, and 72 °C for 5 min at the final in a C1000 Thermal Cycler machine (Bio-Rad, USA).

### Amplification and nucleotide sequencing

In this study, six pairs of primer (Table 1) were designed based on the full sequence of six strains Italy1-AJ489744, Chile-JQ413340, Korea1-JX878304, UK-HM067437, Korea2-JX878305 and USA1-AY292384), used to amplify the full sequence genome of the Vietnamese DWV strains. PCR-amplification was carried out according to Choe et al. (2012a). After gel extraction, PCR products were sequenced directionally by ABI 3500 Genetic Analyzer machine.

**Table 1:** Primers used to obtain the complete nucleotide sequence of DWV isolated from *A. cerana* in Vietnam (in this study).

**Fragments**	**Primer**	**Sequence ofprimers**	**Position**		**Size(Kb)**		
DWVFg1	DWV 1F	5′CGATTTATGCCTTCCATAGCG 3′	1–21		2,37
	DWV 1RR	5′CTATCGCAGAAATTACTAC 3′(primer for sequencing)	727–748				
	DWV 1R	5′TGCCAGTAGTCCAATCTGG 3′	2351–2369				
DWVFg2	DWV2F	5′CCAAGTTGGTCAATTACAAGC 3′	2164–2184)		1,86
	DWV2R	5′CAATGATCAATATCCTTATCCG 3′	4002–4024				
DWVFg3	DWV3F	5′TTGAAGCTATTCCAGAAGG 3′		3831–3849		1,77	
	DWV3R	5′CACAGGAGTACGACTCGCACG 3′	5615–5636		
DWVFg4	DWV4F	5′GGAAATGGGATCGAATCC 3′	5503–5520		1,77
	DWV4R	5′CAACTTGCGGCCACCACTTG 3′	7258–7277				
DWVFg5	DWV5F	5′GGTACCAAGAAGGGTATG 3′	7109–7126		1,71
	DWV5R	5′CAATCCGTGAATATAGTGTGAGG 3′	8798–8820				
DWVFg6	DWV6F	5′CTTGGAATACTAGTGCTGG 3′	8619–8637		1,52
	DWV6FF	5′GATGGAATTTACGGATCAGG 3′(primer for sequencing)	9472–9491				
	DWV6R	5′ACTATTATGGTTAAAACTATAC 3′	10119–10140				

### Genomic, phylogenetic and simplot analysis

The DNASTAR program was used to assemble nucleotide sequences of DWV strains. Aligned sequence of nucleotides and amino acids were analyzed by BioEdit software version 7.0.9.0 (Hall, 1999) with published DWV sequences JX878304- Korea1 and JX878305- Korea2 as references to construct the complete genome sequences. For multiple sequence alignments (MSAs), we used BioEdit software version 7.0.9.0 (Hall, 1999); firstly, the nucleotide sequences were translated into amino acid sequences then the deduced amino acid sequences were used to construct the phylogenetic tree by Mega7 (Kumar, Stecher & Tamura, 2016). Published DWV sequences: aY292384- USA1, JQ413340- Chile, AJ489744- Italy1, KX373899- France, GU109335- UK1, KJ437447- UK2, AB070959- Japan, JX878304- Korea1, JX878305- Korea2, MF770715- China1, MF036686- China2, MH165180- China3, DWV type A, DWV type B, and DWV type C were used to built a phylogenetic tree with the neighbor-joining (NJ) method (Saitou & Nei, 1987), and the genetic distances were computed using Kimura 2 parameter (Kimura, 1980). A bootstrap value of 1,000 replicates was applied to yield a robust phylogeny. The Simplot software (Ray, 2003) was used to plot the differences between the DWV-VN and other strains. The complete genome sequences of DWV strains were multiple aligned and analyzed using the Kimura 2 parameter distance model with a window size of 200 bp and a step size of 20 bp with the gap strip on Satharasinghe et al. (2016) and Li et al. (2020). The percent similarities are shown in the Results section.

## Results

### Genome analysis

The complete genomes of two Vietnamese DWV strains (DWV-VN), firstly isolated from *A. cerana*, were sequenced and published in GenBank under accession numbers: MN607197 (for DWV-NVN strain was isolated from North of Vietnam) and MN607198 (for DWV-SVN strain was isolated from South of Vietnam).

The nucleotide sequences of both DWV-NVN and DWV-SVN genomes comprised 10,113 bp and contained a large single open reading frame (ORF) coded for 2,893 amino acids, initiating at nucleotide 1130 and terminating at nucleotide 9,812. The genetic identities of these two strains were 98.0% and 99.3% for nucleotide sequences and amino acid sequences, respectively. Multiple nucleotide sequence alignment (Table S1) revealed that DWV-VN showed low genetic identities (91.4%–92.0%) with DWV strains in *A. mellifera* such as USA1, Chile, Italy1, France, UK1, Korea1, Korea2, Japan, China1 strains and much lower identity (89.2%, 89.4% and 89.6%) compared to UK2 and China2 strain. The genetic identities between DWV-China3 strain in *A. cerana* and DWV-VN strains were in 91.7% and 91.9%, respectively. When compare the nucleotide sequence at 5′UTR region, they share 93.1%–95.1% identity with those from other countries, and much lower identity (83.1% and 82.8%) compared to the China2 strain. The nucleotide sequence for 3′UTR region showed a high genetic identities (100%) between DWV-NVN strain and DWV-SVN strain. However, the DWV-VN strains shared low genetic identities (90.1%–92.1%) with DWV strains from other countries (including Italy1, UK1, Korea1, Korea2, and China3) and 82.3%–83.3% identity with DWV strains from USA1, Chile, France, UK2, Japan, and China1. Alignment results of amino acid sequences of DWV-VN strains and other DWV strains (Table S1) showed the genetic identities between strains were in 96.7%–97.9%.

The result of multiple sequence alignment of amino acid sequences indicated that DWV-VN strains carried feature structure for DWVs such as VQAKP**E**^**211**^MD, DNPNP**G**^**218**^PD, IRAKP**E**^**464**^MD, IGGNN**M**^**485**^VNP, IEAIP**E**^**901**^GE, VRAVP**E**^**1159**^GP, ISAVP**E**^**1248**^AP, GIAKP**E**^**1760**^MD**,** GLASP**Q**^**2093**^GL**,** TAAFP**E**^**2180**^GT (Lanzi et al., 2006; De Miranda & Genersch, 2010). In the L protein, DWV-VN strains had six substitutions at amino acids 27 (E > I), 98 (S > T), 120 (A > V), 153 (M > T), 170 (D > F), and 174 (Y > F) in comparison to other strains (Fig. S1A). In the VP3 region of DWV-VN strains, we also observed two substitutions at amino acids 980 (S > A) and 1032 (E > T) compared to other strains (Fig. S1B). The alignment of the RdRp sequences pointed out that DWV-VN strains had a substitution at amino acid 2627 R > C, which is different from other strains (Fig. S1C). In addition, in RdRp of DWV-VN strains we also found one substitution at amino acid 2485 (L > V) that was not found in other investigated strains exept UK1 and UK2 strains. Whereas, the amino acid change at positions 2693 (Y > C) was similar to other strains including France, UK1, UK2, Japan, Korea1, Korea2, China1, China2, and China3 strains compared to USA1, Chile, and Italy1 strains.

### Phylogenetic and Simplot analysis

**Figure 1:**
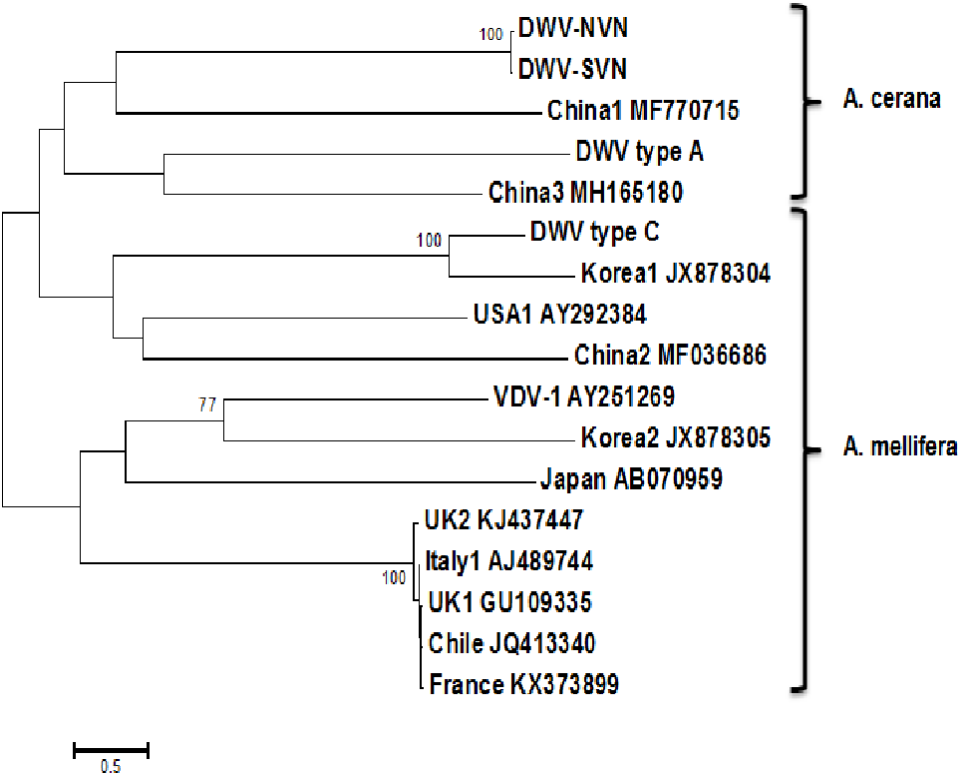
Phylogenetic analysis of the complete sequences. The tree was constructed using multiple sequence alignment of the complete sequence of DWV strains of DWV type A, DWV type B (VDV1-AY251269), DWV type C, USA (USA1-AY292384), Chile (JQ413340), Italy (Italy1-AJ489744), France (KX373899), UK (UK1-GU109335, UK2-KJ437447), Japan (AB070959), Korea (Korea1-JX878304, Korea2-JX878305), China (China1-MF770715, China2-MF036686, and China3-MH165180), and Vietnam (DWV-NVN, DWV-SVN).

The phylogenetic trees were built based on the complete genome sequences and RdRp sequences to compare the genetic relationships between the DWV strains. The tree based on the complete sequences (Fig. 1) showed that DWV strains divided into two distinct groups: the first group containing DWV strains in *A. mellifera*; the second group containing DWV strains in *A. cerana*. However, in this phylogenetic tree, China1 strain (in honeybee *A. mellifera*) also belongs to the group of *A. cerana*. The phylogenetic tree based on the RdRp sequences (Fig. S1D) segregated the DWV strains into two groups: the first group consisting of DWV strains in Asian honeybee and the second group including DWV strains in the Western or Europe honeybee. Interestingly, DWV strains from the UK belonged to a separate clade in the middle between two groups.

**Figure 2:**
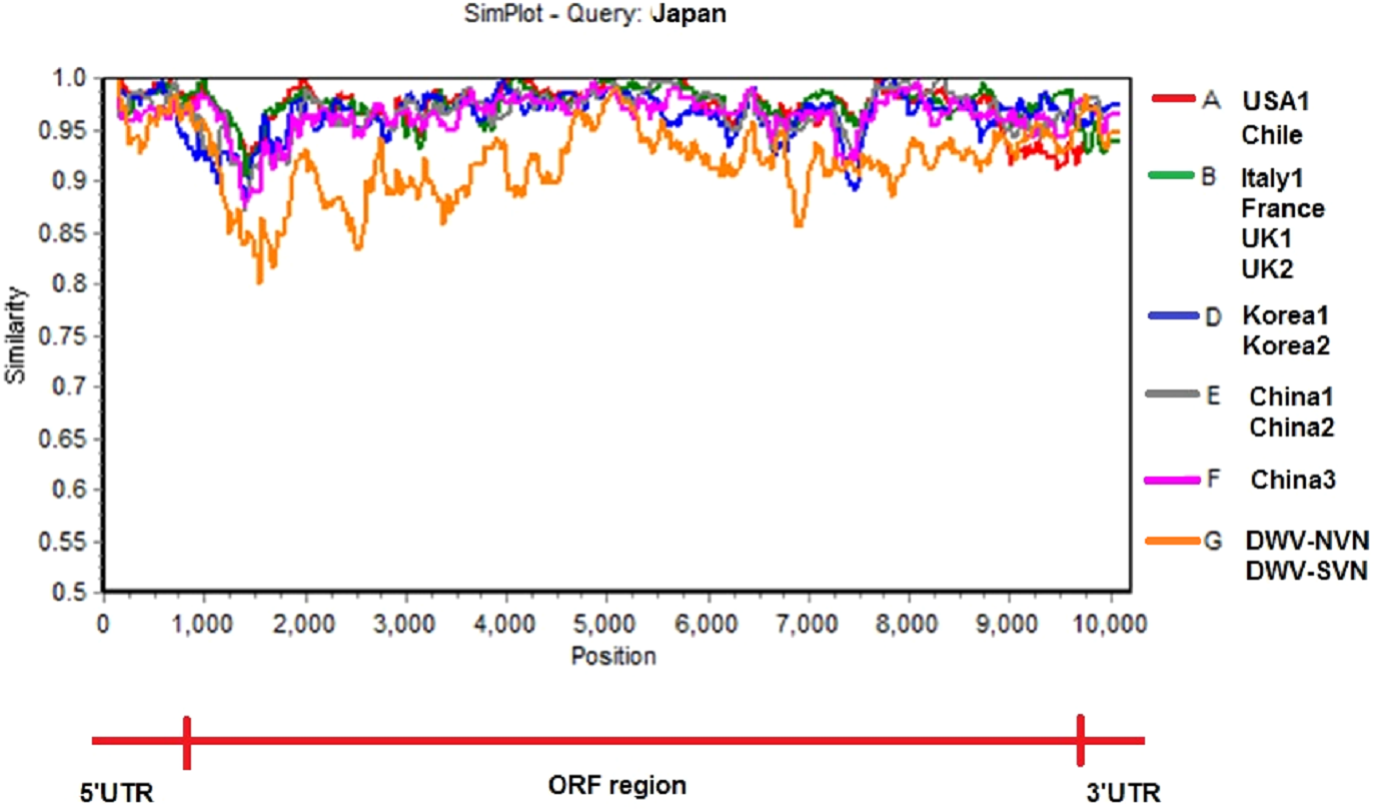
Similarity plot of the two complete DWV-VN genome sequences and other complete genome sequences (available in the GenBank database: AY292384- USA1, JQ413340- Chile, AJ489744- Italy1, KX373899- France, GU109335- UK1, KJ437447- UK2, AB070959- Japan, JX878304- Korea1, JX878305- Korea2, MF770715- China1, MF036686- China2, MH165180- China3, DWV-NVN, and DWV-SVN). The multiple sequence alignment of the complete genome sequences of DWV strains were analyzed with Japan strain using as reference sequence. The SimPlot analysis were performed using the Kimura 2 parameter distance model with a window size of 200 bp and a step size of 20 bp with the gap strip on.

Simplot analysis (Fig. 2 and Fig. S2) showed a significant difference in complete genome sequences between DWV-VN strains and other strains. As shown in Fig. 2, except the 5′UTR, RdRp, and 3′UTR regions, the entire ORF region the DWV-VN genomes were very different from the genomes of other DWVs. In particular, the L, VP1, VP2, and VP3 protein regions (from 1000 to 4500), and the polyprotein region (from 5250 to 9000) were the most different to other DWV strains.

## Discussion

In this study, the sequences of two Vietnamese DWV strains that infected *A. cerana* from different regions of Vietnam were determined. The genome of two DWV-VN strains contained a single large ORF initiating at nucleotide 1,130 and terminating at nucleotide 9,812. The genome of DWV-VN strains was the same with other members in *Iflavirus* family consisting of structural proteins at the 5′ end and the nonstructural proteins at the 3′ end arranged in a unique order. Analysis of nucleotide sequences of DWV-VN strains isolated from *A. cerana* showed the low genetic identities (89.2%–92.0%) when compared to other DWV strains. The low genetic identities were also found in the 5′UTR region between DWV-VN strains and other DWV strains with 82.8%–95.1%. Notably, the 3′UTR region of DWV-VN strains shared 82.3%–92.1% identities with other DWVs, whereas the Korea strains showed a closer relation of 89.7%–94.7% with other strains (Reddy et al., 2013). These results indicated that there was a significant difference between DWV strains in *A. cerana* and *A. mellifera*. The results were remarkable because the 5′UTR and 3′UTR regions play a vital role in translation and stability of the viral mRNA (Song et al., 2006; Kim & Samal, 2010). It can also explain for the difference in DWV infection percentage between *A. cerana* and *A. mellifera*. These ratios may be 8.07% in *A. cerana* and up to 66.6% in *A. mellifera* in Korea (Choi et al., 2008; Choe et al., 2012b). The DWV infection was particularly high, up to over 90% in Europe and USA where *A. mellifera* is widespread (Tentcheva et al., 2004; Baker & Schroeder, 2008; Welch et al., 2009).

Besides, genome analysis showed that there were many substitutions in structural protein region of DWV-VN strains compared to other strains. In the VP3, the substitutions only occurred at two amino acid sites (Fig. S1B). Two substitutions in VP3 region were reported by Benreyi et al., (2007). Interestingly, we observed many amino acid variations in L protein at six different points (Fig. S1A), and these changes were only found in Vietnamese DWV strains but not in other strains even in China3 strain (a DWV strain in *A. cerana* honeybees). It has been known that this region was a hotspot for microvariation (Lanzi et al., 2006). Lanzi et al. (2006) found that the L protein was 5.4-fold variable than the rest of the structural protein and 3.3-fold variable than the nonstructural protein. The L proteins are also different among picornaviruses and are related to pathology through suppression of host cap-dependent mRNA translation (Glaser, Cencic & Skern, 2001) and stimulate the activity of internal ribosome entry site virus (Hinton et al., 2002). The variations found in the L protein suggested that the DWV-VN strains have changed their pathogenicity, however, further investigations are needed to identify the effects of these changes in translation and virulence of the DWV-VN strains. The RdRp is known to be indispensable for replication of the genome of viruses as well as epigenetic control and post-transcription of cellular gene expression (Ng, Arnold & Cameron, 2008). It is highly conserved and plays the role in viral evolution (Elena & Sanjuan, 2005). The increased rates of mutation in this region in the viral population allows the mutation to be selected under the host defence mechanisms and other environmental factors (Crotty, Cameron & Andino, 2001). In our study, the results of amino acid sequence alignment of this region showed that the DWV-VN strains had three amino acid changes in comparison to other strains. The amino acid change at position 2485 (L> V) was also found in the genome of two DWV strains from UK (Fig. S1C). And two amino acid changes at position 2627 and 2693 were found on the strains from UK, Japan, Korea, and China in comparison to strains from USA, Chile, Italy and France. However, there were changes at position 2627 (R> C) in DWV-VN and 2627 (R> S) in other strains.

Phylogenetic tree constructed from complete sequences indicated that the DWV strains isolated from *A. mellifera* and *A. cerana* belonged to two separate groups (Fig. 1). DWV strains in honeybees *A. cerana* belonged to the DWV type A group. Otherwise, DWV strains in bees *A. mellifera* were divided into two subgroups: DWV type B and DWV type C. It has been known that Varroa-DWV interaction has led to the dominance of a particular DWV lineage, supplanting other DWV variation (Ryabov et al., 2014; Wilfert et al., 2016). This result displayed that there was a significant difference between DWV infected in *A. cerana* and those infected in *A. mellifera*. Phylogenetic analysis based on nucleotide sequences of RdRp region showed that the DWV strains isolated from Asian honeybee and the strains isolated from Western and American honeybee were in divided into two separate groups (Fig. S1D), suggesting that the DWV strains have adapted to the geographical conditions in different regions. Zhang et al. (2012) also provided evidence of the important role of genetic relatedness and the geographical proximity in adaptation of the viruses. Also, the results from the Simplot analysis suggested a significant difference between DWV strains circulating in the Vietnamese honeybee and other *A. mellifera* (Fig. 2). This difference was also observed in the China3 strain in *A. cerana*. Interestingly, the Simplot graph (Fig. 2) revealed that there was high variation between the DWV-VN and other strains, especially in the L,VP1, VP2, VP3, and the polyprotein regions, whereas little variation was observed in the 5′UTR, RdRp, and 3′UTR regions between DWV-VN and other strains.

## Conclusions

This is the first report of complete genome sequence of DWV isolated in Vietnamese honeybee *A. cerana*. Our results provide the evidences that the DWV-VN strains infecting honeybee in Vietnam had changed their genome that may help them adapt the geographical conditions and these may lead to change their pathogenicity. Our study also provides useful information for further research on the phylogenetic origins of Vietnamese DWV strains

##  Supplemental Information

10.7717/peerj.9911/supp-1 Supplemental Information 1 Multiple sequence alignment of the amino acid sequences and phylogenetic analysis of the RdRp domains **A.** Multiple sequence alignment of the amino acid sequences in the L protein region of DWV strains (DWV-NVN and DWV-SVN) with those of USA1 (AY292384), Chile (JQ413340), Italy1 (AJ489744), France (KX373899), UK1 (GU109335), UK2 (KJ437447), Japan (AB070959), Korea1 (JX878304), Korea2 (JX878305), China1 (MF770715), China2 (MF036686) and China3 (MH165180). In the L protein region, there were six substitutions in the deduced amino aicd sequence that occurred at amino acids 27 (E¿I), 98 (S¿T), 120 (A¿V), 153 (M¿T), 170 (D¿F), and 174 (Y¿F) in DWV-VN strains when compared to other strains.
**B.** Multiple sequence alignment of the amino acid sequences in the VP3 region of DWV strains (DWV-NVN and DWV-SVN) with those of USA1 (AY292384), Chile (JQ413340), Italy1 (AJ489744), France (KX373899), UK1 (GU109335), UK2 (KJ437447), Japan (AB070959), Korea1 (JX878304), Korea2 (JX878305), China1 (MF770715), China2 (MF036686) and China3 (MH165180). There were two amino acid changes at positions 980 (S¿A) and 1032 (E¿T) that found in DWV-VN strains in comparison to other strains.
**C.** Multiple sequence alignment of the amino acid sequences in the RdRp region of DWV strains (DWV-NVN and DWV-SVN) with those of USA1 (AY292384), Chile (JQ413340), Italy1 (AJ489744), France (KX373899), (UK1 (GU109335), UK2 (KJ437447), Japan (AB070959), Korea1 (JX878304), Korea2 (JX878305), China1 (MF770715), China2 (MF036686) and China3 (MH165180). There were one amino acid change at position 2627 R¿C in DWV-VN strains, which is different from other strains and other one at position 2485 (L¿V) that found in DWV-VN strains and UK strains.
**Fig. S1D.** Phylogenetic analysis of the RdRp domains. The tree was constructed using multiple sequence alignment of the RdRp sequence of DWV strains in USA (USA1-AY292384), Chile (JQ413340), Italy (Italy1-AJ489744), France (KX373899), UK (UK1-GU109335, UK2-KJ437447), Japan (AB070959), Korea (Korea1-JX878304, Korea2-JX878305), China (China1-MF770715, China2-MF036686, and China3-MH165180), and Vietnam (DWV-NVN, DWV-SVN).

10.7717/peerj.9911/supp-2 Supplemental Information 2 Multiple alignment sequence of complete genome sequence of DWV strains in this study

10.7717/peerj.9911/supp-3 Supplemental Information 3 Nucleotide sequences and amino acid sequences for the coding region homology (%) between DWV-VN and the other reference sequences

## Additional Information and Declarations

### Field Study Permissions

The following information was supplied relating to field study approvals (i.e., approving body and any reference numbers): Field experiments were approved by the Research Center for Tropical Bees and Beekeeping Vietnam National University of Agriculture, Hanoi, Vietnam and the hive owner.

### DNA Deposition

The following information was supplied regarding the deposition of DNA sequences: Sequences are available at Genbank: MN607197 and MN607198.

### Data Availability

The following information was supplied regarding data availability: The raw measurements are available in the Supplemental Files.
