# Supplementary material for: Genome analysis and phylogenetic characterization of two deformed wing virus strains from Apis cerana in Vietnam
Source: PeerJ. 2020 Sep 21;8:e9911. doi: 10.7717/peerj.9911 (PMC7513742; doi:10.7717/peerj.9911)
Supplement: Supplemental Information 1 — A. Multiple sequence alignment of the amino acid sequences in the L protein region of DWV strains (DWV-NVN and DWV-SVN) with those of USA1 (AY292384), Chile (JQ413340), Italy1 (AJ489744), France (KX373899), UK1 (GU109335), UK2 (KJ437447), Japan (AB070959), Korea1 (JX878304), Korea2 (JX878305), China1 (MF770715), China2 (MF036686) and China3 (MH165180). In the L protein region, there were six substitutions in the deduced amino aicd sequence that occurred at amino acids 27 (E¿I), 98 (S¿T), 120 (A¿V), 153 (M¿T), 170 (D¿F), and 174 (Y¿F) in DWV-VN strains when compared to other strains. B. Multiple sequence alignment of the amino acid sequences in the VP3 region of DWV strains (DWV-NVN and DWV-SVN) with those of USA1 (AY292384), Chile (JQ413340), Italy1 (AJ489744), France (KX373899), UK1 (GU109335), UK2 (KJ437447), Japan (AB070959), Korea1 (JX878304), Korea2 (JX878305), China1 (MF770715), China2 (MF036686) and China3 (MH165180). There were two amino acid changes at positions 980 (S¿A) and 1032 (E¿T) that found in DWV-VN strains in comparison to other strains. C. Multiple sequence alignment of the amino acid sequences in the RdRp region of DWV strains (DWV-NVN and DWV-SVN) with those of USA1 (AY292384), Chile (JQ413340), Italy1 (AJ489744), France (KX373899), (UK1 (GU109335), UK2 (KJ437447), Japan (AB070959), Korea1 (JX878304), Korea2 (JX878305), China1 (MF770715), China2 (MF036686) and China3 (MH165180). There were one amino acid change at position 2627 R¿C in DWV-VN strains, which is different from other strains and other one at position 2485 (L¿V) that found in DWV-VN strains and UK strains. Fig. S1D. Phylogenetic analysis of the RdRp domains. The tree was constructed using multiple sequence alignment of the RdRp sequence of DWV strains in USA (USA1-AY292384), Chile (JQ413340), Italy (Italy1-AJ489744), France (KX373899), UK (UK1-GU109335, UK2-KJ437447), Japan (AB070959), Korea (Korea1-JX878304, Korea2-JX878305), China (China1-MF770715, China2-MF036686, and China3-MH165 [file peerj-08-9911-s001.pdf]

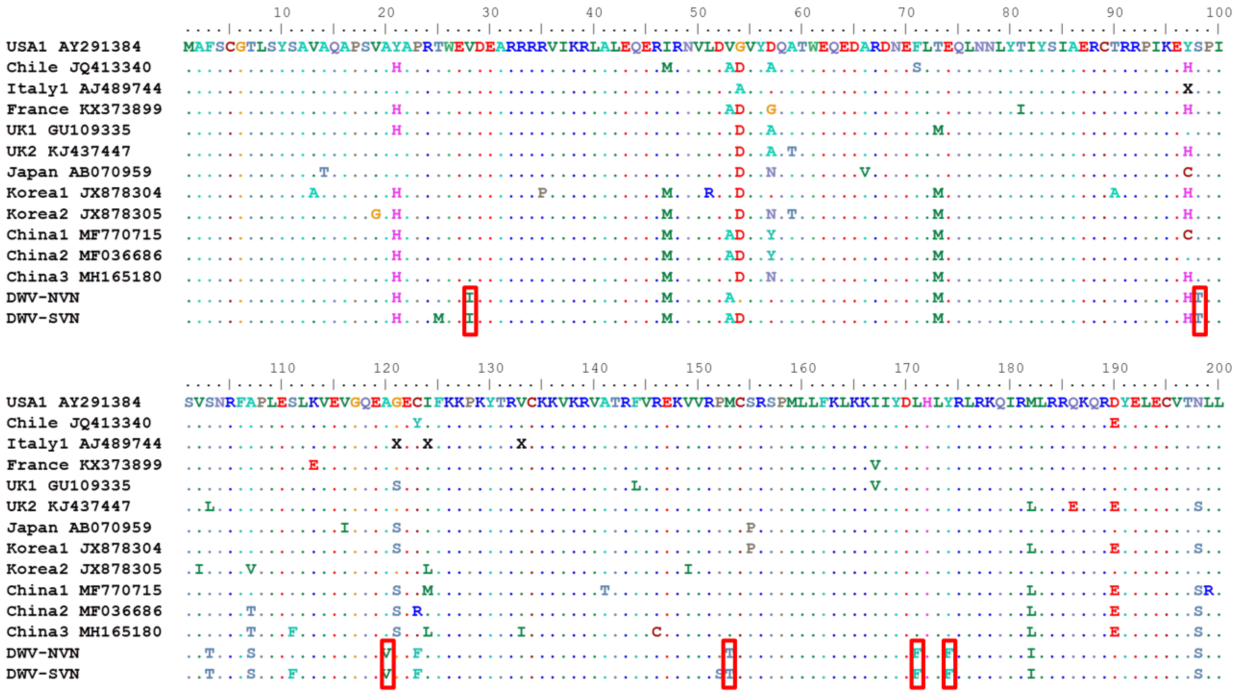

Fig. S1A. Multiple sequence alignment of the amino acid sequences in the L protein region of DWV strains (DWV-NVN and DWV-SVN) with those of USA1 (AY292384), Chile (JQ413340), Italy1 (AJ489744), France (KX373899), UK1 (GU109335), UK2 (KJ437447), Japan (AB070959), Korea1 (JX878304), Korea2 (JX878305), China1 (MF770715), China2 (MF036686) and China3 (MH165180). In the L protein region, there were six substitutions in the deduced amino aicd sequence that occurred at amino acids 27 (E>I), 98 (S>T), 120 (A>V), 153 (M>T), 170 (D>F), and 174 (Y>F) in DWV-VN strains when compared to other strains.

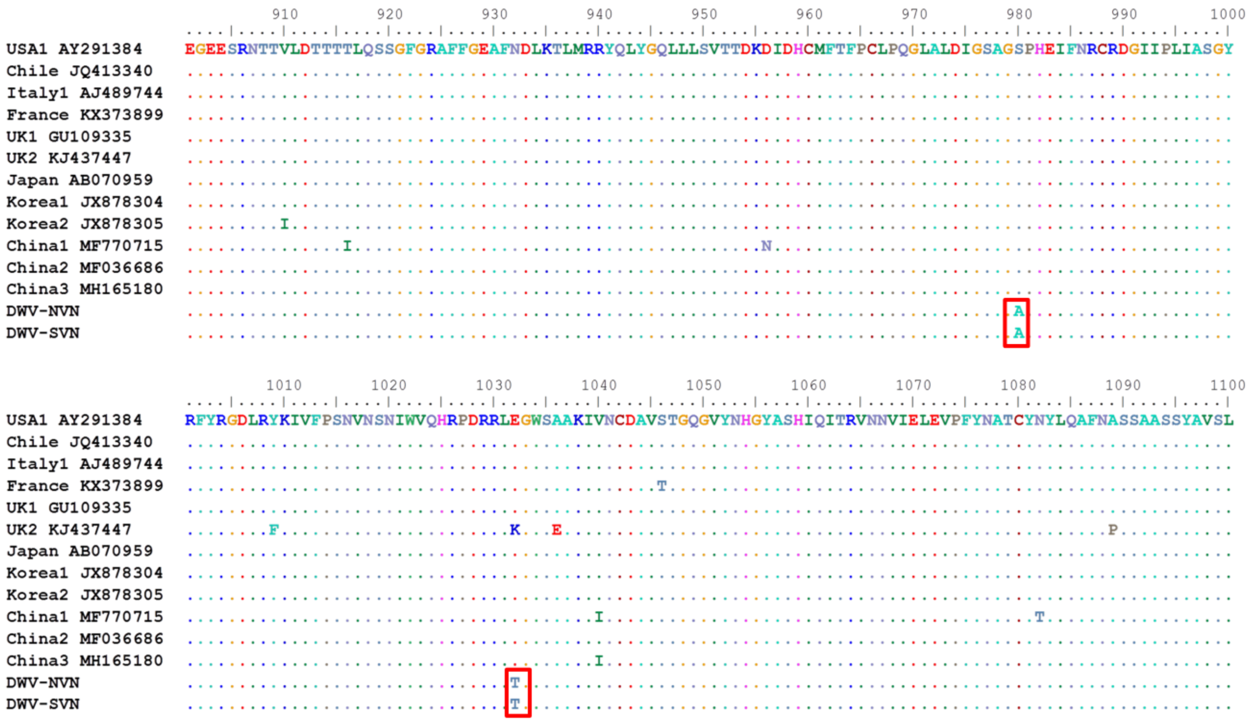

Fig. S1B. Multiple sequence alignment of the amino acid sequences in the VP3 region of DWV strains (DWV-NVN and DWV-SVN) with those of USA1 (AY292384), Chile (JQ413340), Italy1 (AJ489744), France (KX373899), UK1 (GU109335), UK2 (KJ437447), Japan (AB070959), Korea1 (JX878304), Korea2 (JX878305), China1 (MF770715), China2 (MF036686) and China3 (MH165180). There were two amino acid changes at positions 980 (S>A) and 1032 (E>T) that found in DWV-VN strains in comparison to other strains.

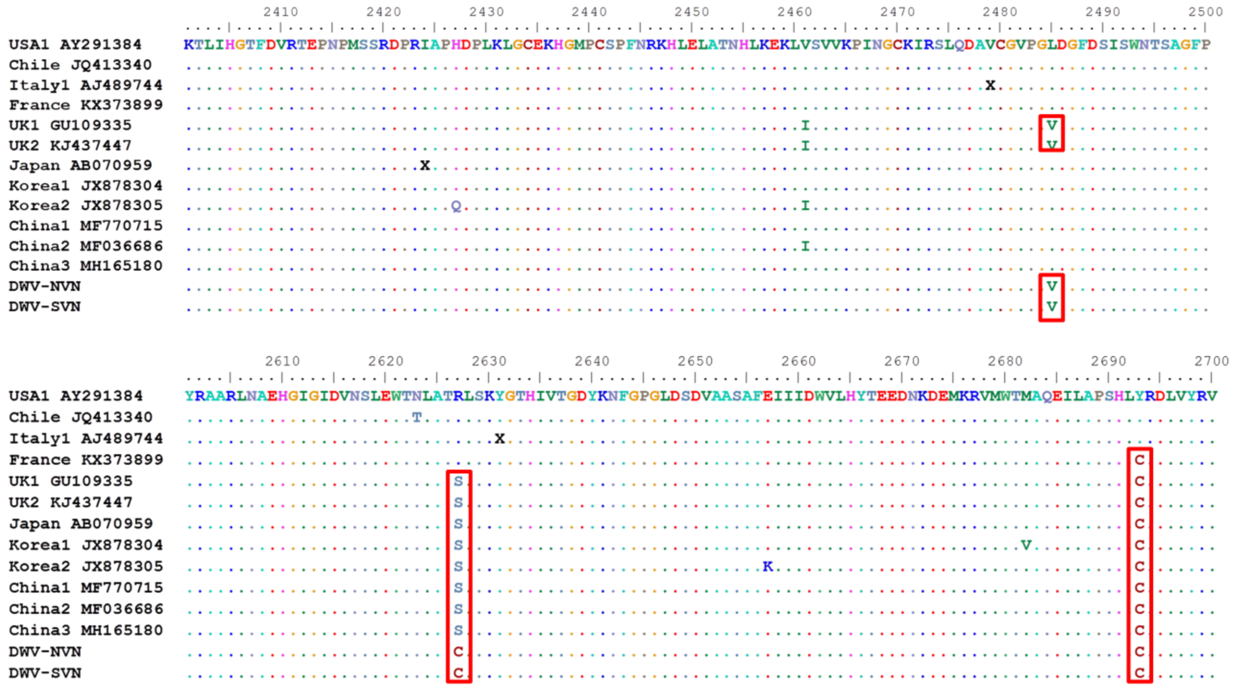

Fig. S1C. Multiple sequence alignment of the amino acid sequences in the RdRp region of DWV strains (DWV-NVN and DWV-SVN) with those of USA1 (AY292384), Chile (JQ413340), Italy1 (AJ489744), France (KX373899), (UK1 (GU109335), UK2 (KJ437447), Japan (AB070959), Korea1 (JX878304), Korea2 (JX878305), China1 (MF770715), China2 (MF036686) and China3 (MH165180). There were one amino acid change at position 2627 R>C in DWV-VN strains, which is different from other strains and other one at position 2485 (L>V) that found in DWV-VN strains and UK strains.

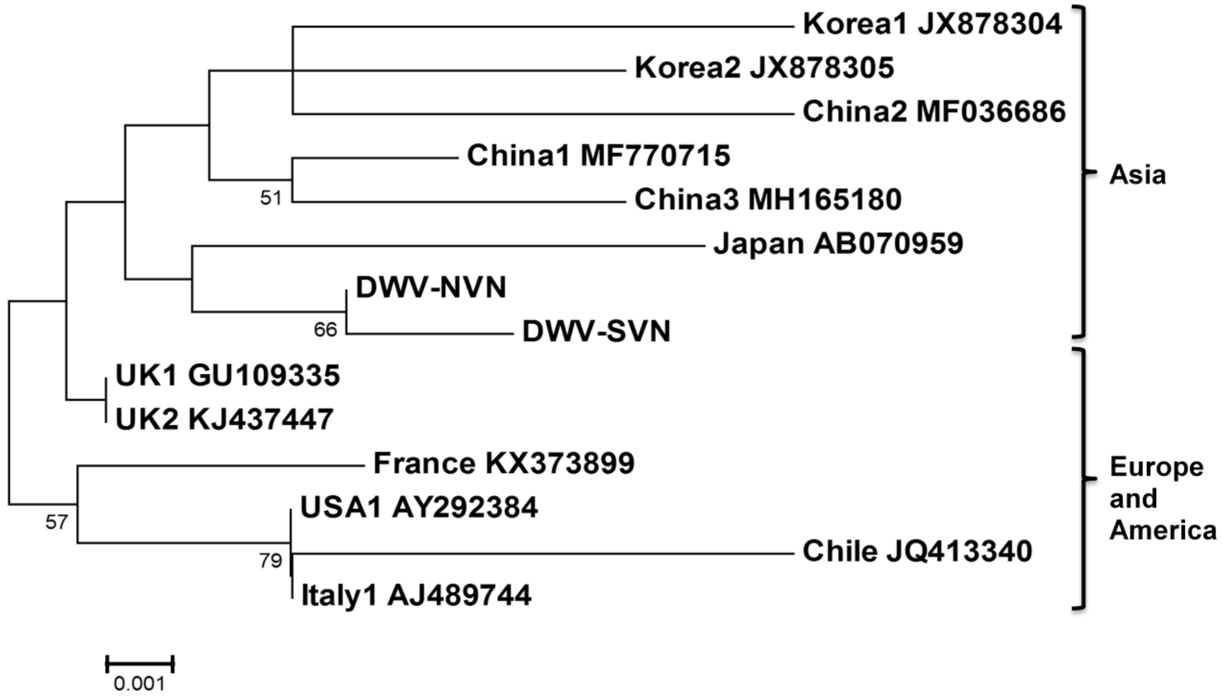

Fig. S1D. Phylogenetic analysis of the RdRp domains. The tree was constructed using multiple sequence alignment of the RdRp sequence of DWV strains in USA (USA1-AY292384), Chile (JQ413340), Italy (Italy1-AJ489744), France (KX373899), UK (UK1-GU109335, UK2-KJ437447), Japan (AB070959), Korea (Korea1-JX878304, Korea2-JX878305), China (China1-MF770715, China2-MF036686, and China3-MH165180), and Vietnam (DWV-NVN, DWV-SVN).
