# Supplementary material for: Genome analysis and phylogenetic characterization of two deformed wing virus strains from Apis cerana in Vietnam
Source: PeerJ. 2020 Sep 21;8:e9911. doi: 10.7717/peerj.9911 (PMC7513742; doi:10.7717/peerj.9911)
Supplement: Supplemental Information 2 [file peerj-08-9911-s002.pdf]

Fig. S2. Multiple alignment sequence of complete genome sequence of DWV strains in this study.

```
                         10        20        30        40        50        60        70        80        90       100
                ....|....|....|....|....|....|....|....|....|....|....|....|....|....|....|....|....|....|....|....|
Japan AB070959  GAATTCTAGGGCGATTTATGCCTTCCATAGCGAATTACGGTGCAACTAACAATTTTAGATAGTAGCCATGAACAAACATTATAG-TAGCTCAC-TACGTA
USA1 AY291384   ----------------TATGCCTTCCATAGCGAATTACGGTGCAACTAACAATTTTAGATAGTAGCCATGAACAAACATTATAG-TGGCTCAC-TACGTA
Chile JQ413340  -----------CGATTTATGCCTTCCATAGCGAATTACGGTGCAACTAACAATTTTAGATAGTAGCCATGAACAAACATTATAG-TAGCTCAC-TACGTA
Italy1 AJ489744 -----------CGATTTATGCCTTCCATAGCGAATTACGGTGCAACTAACAATTTTAGATAGTAGCCATGAACAAACATTATAG-TAGCTCAC-TACGTA
France KX373899 -----------CGATTTATGCCTTCCATAGCGAATTACGGTGCAACTAACAATTTTAGATAGTAGCCATGAACAAACATTATAG-TAGCTCAC-TACGTA
UK1 GU109335    -----------CGATTTATGCCTTCCATAGCGAATTACGGTGCAACTAACAATTTTAGATAGTAGCCATGAACAAACATTATAG-TGGCTCAC-TACGTA
UK2 KJ437447    -----------CGATTTATGCCTTCCATAGCGAATTACGGTGCAACTAACAATTTTAGATAGTAGCCATGAACAAACATTATAG-TGGCTCAC-TACGTA
Korea1 JX875304 ----------------------------AGCGAATTACGGTGCAACTAACAATTTTAGATAGTAGCCATGAACAAACATTATAG-TAGCTCAC-TACGTA
Korea2 JX878305 ----------------------------AGCGAATTACGGTGCAACTAACAATTTTAGATAGTAGCCATGAACAAACATTATAGGTAGCTCACCTACGTA
China1 MF770715 -----------CGATTTATGCCTTCCATAGCGAATTACGGTGCAACTAACAATTTTAGATAGTAGCCATGAACAAACATTATAG-TGGCTCAC-TACGTA
China2 MF036686 ----------------------------------------------------------------------------------------------------
China3 MH165180 -----------------------TCCATAGCGAATTACGGTGCAACTAACAATTTTAGATAGTAGCCACGAACAAACATTATAG-TAGCTCAC-TACGTA
DWV-NVN         -----------------------TCCATAGCGAATTACGGTGCAACTAACAATTTTAGATAGTAGCCATGAACAAACATTATAA-TAGCTCAC-TACGTA
DWV-SVN         -----------------------TCCATAGCGAATTACGGTGCAACTAACAATTTTAGATAGTAGCCATGAACAAACATTATAA-TAGCTCAC-TACGTA

                        110       120       130       140       150       160       170       180       190       200
                ....|....|....|....|....|....|....|....|....|....|....|....|....|....|....|....|....|....|....|....|
Japan AB070959  TTGATCATTTTTATAATGACTTGCGTAGYATGAAGCGC-TGCTTGTAGTTGTAACTATGTTAYTTTGCAAGTTGGAGTTTACTATTTTGGATTATGAATA
USA1 AY291384   TTGATCATTTTTATAATGACTTGCGTAGCATGAAGCGCATGCTTGTAGTTGTAACTATGTTACTTTGCAAGTTGGAGTTTACTATTTTGGATTATGAATA
Chile JQ413340  TTGATCATTTTTATAATGACTTGCGTAGCATGAAGCGCATGCTTGTAGTTGTAACTATGTTACTTTGCAAGTTGGAGTTTACTATCTTGGATTATGAGTA
Italy1 AJ489744 TTGATCATTTTTATAATGACTTGCGTAGCATGAAGCGCATGCTTGTAGTTGTAACTATGTTACTTTRCAAGTTGGAGTTTACTATYTTGGATTATGAATA
France KX373899 TTGATCATTTTTATAATGACTTGCGTAGCATGAAGCGCATGCTTGTAGTTGTAACTATGTTACTTTGCAAGTTGGAGTTTACTATTTTGGATTATGAATA
UK1 GU109335    TTGATCATTTTTATAATGACTTGCGTAGCATGAAGCGCATGCTTGTAGTTGTAACTATGTTACTTTACAAGTTGGAGTTCACTATCTTGGATTATGAGTA
UK2 KJ437447    TTGATCATTTTTATAATGACTTGCGTAGCATGAAGCGCATGCTTGTAGTTGTAACTATGTTACTTTACAAGTTGGAGTTCACTATCTTGGATTATGAGTA
Korea1 JX875304 TTGATCATTTTTAGAATGACTTGCGTAGCATGAAGCGCATGCTTGTAGTTGTAACTATGTTACTTTGCAAGTTGGAGTTTACTATCTTGGATTATGAATA
Korea2 JX878305 TTGATCATTTTTAGAATGACTTGCGTAGCATGAAGCGCATGCTTGTAGTTGTAACTATGTTACTTTGCAAGTTGGAGTTTACTATCTTGGATTATGAATA
China1 MF770715 TTGATCATTTTTACAATGACTTGCGTAGCATGAAGCGCATGCTTGTAGTTGTAACTATGTTACTTTGCAAGTTGGAGTTTACTATTTTGGATTATGAATA
China2 MF036686 ------------------------------------------------CTCCGA--ATGTT-CTTTGCA-GTTGGAGTTTACTATTTTGGATTATGAATA
China3 MH165180 TTGATCATTCTTACAATGACTTGCGTAGCATGAAGCGCATGCTTGTAGTTGTAACCATGTTACTTTGCAAGTTGGAGTTTACTATCTTGGATTATGAATA
DWV-NVN         TTGATCATTTTTACAATGACTTGCGTAGCATGAAGCGCATGCTTGTAGTTGTAACTATGTTACTTTGCAAGTTGGAGTTTATTATTTTGGATTATGAATA
DWV-SVN         TTGATCATTTTTACAATGACTTGCGTAGCATGAAGCGCATGCTTGTAGTTGTAACTATGTTACTTTGCAAGTTGGAGTTTATTATTTTGGATTATGAATA

                        210       220       230       240       250       260       270       280       290       300
                ....|....|....|....|....|....|....|....|....|....|....|....|....|....|....|....|....|....|....|....|
Japan AB070959  TGTGCACTTAGTGTCTGTATTTATAGTCGTTTGTGGTTCAAGGTTTTGTGTTAGTAGTACATTTATGTATGAATGTACCTTTAGTATGAATGTTATAGAA
USA1 AY291384   TGTGCACTTAGTGTCTGTATTTATAGTCGTTTGTGGTTCAAGGTTTTGTGTTAGTAGTACACTTATGTATGAATGTACCTTTAGTATGAATGTTATAGAA
Chile JQ413340  TGTGCACTTAGTGTCTGTATTTATAGTCGTTTGTGGTTCAAGGTTTTGTGTTAGTAGTACACTTATGTATGAATGTACCTTTAGTATGAATGTTATAGAA
Italy1 AJ489744 TGTGCACTTAGTGTCTGTATTTATAGTCGTTTGTGGTTCAAGGTTTTGTGTTAGTAGTACACTTATGTATGAATGTAYCTTTAGTATGAATGTTATAGAA
France KX373899 TGTGCACTTAGTGTCTGTATTTATAGTCGTTTGTGGTTCAAGGTTTTGTGTTAGTAGTACACTTACGTATGAATGTACCTTTAGTATGAATGTTACAGAA
UK1 GU109335    TGTGCACTTAGTGTCTGTATTTATAGTCGTTTGTGGTTCAAGGTTTTGTGTTAGTAGTACACTTATGTATGAATGTACCTTTAGTATGAATGTTATAGAA
UK2 KJ437447    TGTGCACTTAGTGTCTGTATTTATAGTCGTTTGTGGTTCAAGGTTTTGTGTTAGTAGTACACTTATGTATGAATGTACCTCTAGTATGAATGTTATAGAA
Korea1 JX875304 TGTGCACTTAGTGTCTGTATTTATAGTCGTTTGTGGTTCAAGGTTTTGTGTTAGTAGTACACTTATGTATGAATGTACCTTTAGTATGAATGTTATAGAA
Korea2 JX878305 TGTGCACTTAGTGTCTGTATTTATAGTCGTTTGTGGTTCAAGGTTTTGTGTTAGTAGTACACTTATGTATGAATGTACCTTTAGTATGAATGTTATAGAA
China1 MF770715 TGTGCACTTAGTGTCTGTATTTATAGTCGTTTGTGGTTCAAGGTTTTGTGTTAGTAGTACATTTATGTATGAATGTACTCTTAGTATGAATGTTATAGAA
China2 MF036686 TGTGCACTTAGTGTCTGTATTTATAGTCGTTTGTGGTTCAAGGTTTTGTGTTAGTAGTACATGTATGTATGAATGTACCTTTAGTATGAATGTTATAGAA
China3 MH165180 TGTGCACTAAGTGTCTGCATTTATAGTCGTTTGTGGTTCAAGGTTTTGTGTTAGTAGTACACTTATGAATGAATGTACCTTTAGTATGAATGTTATAGAA
DWV-NVN         TGTGCACTTAGTGTCTGTATTTATAGTCGTTTATGGTTCAAGGTTTTGTGTTAGTAGTACGTTTATGTATGAATGTACCCTTAGTATGAATGTTATAGAA
DWV-SVN         TGTGCACTTAGTGTCTGTATTTATAGTCGTTTGTGGTTCAAGGTTTTGTGTTAGTAGTACGCTTATGTATGAATGTACCCTTAGTATGAATGTTATAGAA

                        310       320       330       340       350       360       370       380       390       400
                ....|....|....|....|....|....|....|....|....|....|....|....|....|....|....|....|....|....|....|....|
Japan AB070959  TGACAATATCGAAGGAAAAATCTTTATAAAATA-CAAAAATATTGTTTTWATTACTTCGATATGGTGTTTTATAGAGTAGATTGCCATGTGACCGCTCAT
USA1 AY291384   TGACAATATCGAAAGAAAAATCTTTATAAAATA-CAAAAATATTGTTTTTATTATTTCGATATGGTGTTTTATAGAGTAGATTGCCATGTGACCGCTCGT
Chile JQ413340  TGACAATATCGAAGGAAAAATTTTTATAAAATA-CAAAAATATTGTTTTTATTATTTCGATATGGTGTTTTATAGAGTAGATTGCCATGTGACCGCTCAT
Italy1 AJ489744 TGACAATATCGAARGAAAAATYTTTATAAAATA-CAAAAATATTGTTTTTATTATTTCGATATGGTGTTTTATAGAGTAGATTGCCATGTGACCGCTCRT
France KX373899 TGACAATATCGAAAGAAAAATCCTTATAAAATA-CAAAAATATTGCTTTTATTATTTCGATATGGTGTTTTATAGAGTAGATTGCCATGTGACCGCTCAT
UK1 GU109335    TGACAGTATCGAAGGAAAAATTTTTATAAAATA-CAAAAATATTGTTCTTATTATTTCGATACGGTGTTTTATAGAGTAGATTGCCATGTGACCGCTCAT
UK2 KJ437447    TGACAGTATCGAAGGAAAAATCTTTATAAAATA-CAAAAATATTGTTTTTATTATTTCGATACGGCGTTTTATAGAGTAGATTGCCATGTGACCGCTCAT
Korea1 JX875304 TGACAATATCGAAAGAAAAATCTTTATAAAATA-CAAAAATATTGTTTTTATTATTTCGATATGGTGTTTTATAGAGTAGATTGCCATGTGACCGCTCGT
Korea2 JX878305 TGACAATATCGAAAGAAAAATCTTTATAAAATA-CAAAAATATTGTTTTTATTATTTCGATATGGTGTTTTATAGAGTAGATTGCCATGTGACCGCTCGT
China1 MF770715 TGACAATATCGAAAGAAAAATCTTTATAAAAATACAAAAATATTATTTTTATTATTTCGATATGGTGTTTTATAGAGTAGATTGCCATGTGACCGCTCAT
China2 MF036686 TGACAATATCGAAAGAAAAATCTTTATAAAATA-CAAAAATATTGTTTTTATTATTTCGATATGGTGTTTTATAGAGTAGATTGCCATGTGACCGCTCAT
China3 MH165180 TGACAATATCGAAAGAAAAATCTTTATAAAATATAAAAAATATTGTTTTTATTATTTCGATATGGTGTTTTATAGAGTAGATTGCCATGTGACCGCTCGT
DWV-NVN         TGACAATATCGAAAGAAAA-CTCTTATAAAATA-CAAAAATATTATTTATGTTATTTCGATATGGTGTTTTATAGAGTAGATTGCCATGTGACCGCTCAT
DWV-SVN         TGACAATATCGAAAGAAAA-CCCCTATAAAATA-CAAAAATATTATTTATATTATTTCGATATGGTGTTTTATAGAGTAGATTGCCATGTGACCGCTCAT
```

```
                             410       420       430       440       450       460       470       480       490       500
                    ....|....|....|....|....|....|....|....|....|....|....|....|....|....|....|....|....|....|....|....|
Japan AB070959      AGAAGTCCATTATGGTTTATCAATCGAAGTTGAATGTATTTATAAGAATATTATACTTAATTAGTAATATTAGTAGTCCGTAACTATTATCATCCTTTTT
USA1 AY291384       AGAAGTCCATTATGGTTTATCAATCGAAGTTGAATGTATTTATAAGAATATTATACTTAATTAGTAATATTAGTAGTCCGTAACTATTATCATCCTTTT-
Chile JQ413340      AGAAGTCCATTATGGTTTATCAATCGAAGTTGAATGTACTTATAAGAATATTATACTTAATTGGTAATATTAGTAGTCCGTAACTATTATCATCCTTTT-
Italy1 AJ489744     AGAAGTCCATTATGGTTTATCAATCGAAGTTGAATGTATTTATAAGRATATTATACTTAATTAGTAATATTAGTAGTCCGTAACTATTATCATCCTTTT-
France KX373899     AGAAGTCCATTATGGTTTATCAATCGAAGTTGAATGTATGTATAAGGATATTATACTTAATTAGTAATATTAGTAGTCCGTAACTATTATCATCCTTTT-
UK1 GU109335        AGAAGTCCATTATGGTTTATCAATCGAAGTTGAATGTATTTATAAGGATATTATACTTAATTAGTAATATTAGTAGTCCGTAACTATTATCATCCTTTT-
UK2 KJ437447        AGAAGTCCATTATGGTCTATCAATCGAAGTTGAATGTATTTATAAGGACATTATACTTAATTAGTAATATTAGTAGTCCGTAACTATTATCATCCTTTT-
Korea1 JX875304     AGAAGTCCATTATGGTTTATCAATCGAAGTTGAATGTATTTATAAGAATATTCTACTTAATTAGTAATATTAGTAGTCCGTAACTATTATCATCCTTTT-
Korea2 JX878305     AGAAGTCCATTATGGTTTATCAATCGAAGTTGAATGTATTTATAAGAATATTCTACTTAATTAGTAATATTAGTAGTCCGTAACTATTATCATCCTTTT-
China1 MF770715     AGAAGTCCATTATGGTTTATCAATCGAAGTTGAATGTATTTATAAGAATATTCTATTTAATTAGTAATATTAGTAGTCCGTAACTATTGTCATCCTTTT-
China2 MF036686     AGAAGTCCATTATGGTTTATCAATCGAAGTTGAATGTATTTATAAGAATATTATACTTAATTAGTAATATTAGTAGTCCGTAACTATTGTCATCCTTTT-
China3 MH165180     AGAAGTCCATTATGGTTTATCAATCGAAGTTGAATGTATCTATAAGAATATTCTACTTAATTAGTAATATAAGTAGTCCGTAACTATTATCATCCTTTT-
DWV-NVN             AGAAGTCCATTATGGTTTATCAATCGAAGTTGAATGTACTTATAAGAGCATTGTACTTAATTAGTAATATTAGTAGTCCGTAACTATTATCGTCCTTTT-
DWV-SVN             AGAAGTCCATTATGGTTTATCAATCGAAGTCGAATGTACTTATAAGAACATTGTACTTAATTAGTAATATTAGTAGTCCGTAACTATTATCATCCTTTT-
```

```
                             510       520       530       540       550       560       570       580       590       600
                    ....|....|....|....|....|....|....|....|....|....|....|....|....|....|....|....|....|....|....|....|
Japan AB070959      TACAGTTTGATGTGATAATAGACCACTGCAGTATCGAGTAGAGTTTCGAATGCGTAGTGCAATAGTACAATCACTGTCACCGACCATCTATTGTAATGAT
USA1 AY291384       -TCAGTTTGATGTGATAATAGACCACTGCAGTATCGAGTAGAGTTTCGAATGCGTAGTGCAATAGTACAATCACTGTCACCGACCATCTATTGTAATGAT
Chile JQ413340      -TCAGTTTGATGTGATAATAGACCACTGCAGTATCGAGTAGAGTTCCGAATGCGTAGTGCAATAGTATAATCACTGTCACCGACCATCTATTGTAATGAT
Italy1 AJ489744     -TCAGTTTGATGTGATAATAGACCACTGCAGTATCGAGTAGAGTTTCGAATGCGTAGTGCAATAGTATAATYACTGTCACCGACCATCTATTGTAATGAT
France KX373899     -TCAGTTTGATGTGATAATAGACCACTGCAGTATCGAGTAGAGTTTCGAATGCGTAGTGCAATAGTATAATCACTGTCACCGACCATCTATTGTAATGAT
UK1 GU109335        -TCAGTTTGATGTGATAATAGACCACTGCAGTATCGAGTAGAGTTTCGAATGCGTAGTGCAATAGTATAATCACTGTCACCGACCATCTATTGTAATAAT
UK2 KJ437447        -TCAGTTTGATGTGATAATAGACCACTGCAGTATCGAGTAGAGTTTCGGATGCGTAGTGCAATAGTATAATCACTGTCACCGACCATCTATTGTAATGAT
Korea1 JX875304     -ACAGTTTGATGTGATAATAGACCACTGCAGTATCGAGTAGAGTTTCGAATGCGTAGTGCAATAGTATAATCACTGTCACCGACCATCTATTGTAATGAT
Korea2 JX878305     -ACAGTTTGATGTGATAATAGACCACTGCAGTATCGAGTAGAGTTTCGAATGCGTAGTGCAATAGTATAATCACTGTCACCGACCATCTATTGTAATGAT
China1 MF770715     -ATAGTTTGATGTGACAATAGACCACTGCAGTATCGAGTAGAGTTTCGAAAGCGTAGTGCAATAGTATAATCACTGTCACCGACCATCTATCGTAATGAT
China2 MF036686     -ATAGTTTGATGTGACAATAGACCACTGCAGTATCGAGTAGAGTTTCGAACGCGTAGTGCAATAGTATAATCACTGTCACTGACCATCTATTGTAATGAT
China3 MH165180     -ACAGTTTGATGTGATAATAGACCGCTGCAGTATCGAGTAGAGTTTCGAATGCGTAGTGCAATAGTATAATCACTGTCACCGACCATCTATTGTAATGGT
DWV-NVN             -ACAGTTTGATGTGATAATAGACCACTGCAGTATCGAGTAGAGTTTTGAACGCGTAGTGCAATAGTATAATCACTGTCACCGACCATCTATTGTAATGAT
DWV-SVN             -ACAGTTTGATGTGATAATAGACCACTGCAGTATCGAGTAGAGTTTCGAACGCGTAGTGCAATAGTATAATCACTGTCACCGACCATCTATTGTAATGGT
```

```
                             610       620       630       640       650       660       670       680       690       700
                    ....|....|....|....|....|....|....|....|....|....|....|....|....|....|....|....|....|....|....|....|
Japan AB070959      AGATCTGTCGGAAACCATTATTTATGAAGTGACTAGCAATCATGGATTAAATTAGATG-GTATTCTAGTTTAGAGGTGATTCGGCGCTGCGGTGCGACTG
USA1 AY291384       AGATTTGTCGGAAACCATTATTTATGAAGTGACTAGCAATCATGGATTAAATTAGATG-GTATTCTAGTTTAGAGGTGATTCGGCGCTGCGGTGCGACTG
Chile JQ413340      AGATCTGTCGGAAACCATTATTTATGAAGTGACTAGCAATCATGGATTAAATTAGATG-GTATTCTAATTTAGAGGTGATTCGGCGCTGCGGTGCGACTG
Italy1 AJ489744     AGATCTGTCGGAAACCATTATTTATGAAGTGACTAGCAATCATGGATTAAATTAGATG-GTATTCTAGTTTAGAGGTGATTCGGCGCTGCGGTGCGACTG
France KX373899     AGATCTGTCGGAAACCATTATTTATGAAGTGACTAGCAATCATGGATTAAATTAGATG-GTATTCTAGTTTAGAGGTGATTCGGCGCTGCGGTGCGACTG
UK1 GU109335        AGATCTGTCGGAAACCATTATTTATGAAGTGACTAGCAATCATGGATTAAATTAGATG-GTATTCTAGTTTAGAGGTGATTCGGCGCTGCGGTGCGACTG
UK2 KJ437447        AGATCTGTCGGAAACCATTATTTATGAAGTGACTAGCAATCATGGATTAAATTAGATG-GTATTCTAGTTTAGAGGTGATTCGGCGCTGCGGTGCGACTG
Korea1 JX875304     AGATCTGTCGGAAACCATTATTTATGAAGTGACTAGCAATCATGGATTAAATTAGATG-GTATTCTAGTTTAGAGGTGATTCGGCGCTGCGGTGCGACGA
Korea2 JX878305     AGATCTGTCGGAAACCATTATTTATGAAGTGACTAGCAATCATGGATTAAATTAGATG-GTATTCTAGTTTAGAGGTGATTCGGCGCTGCGGTGCGACTG
China1 MF770715     AGATCTGTCGGAAACCATTATTTATGAAGTGACTAGCAATCATGGATTAAATTAGATG-GTATTCTAGTTTAGAGGTGATTCGGCGCTGCGGTGCGACTG
China2 MF036686     AGATCTGTCGGAAACCATTATTTATGAAGTGACTAGCAATCATGGATTAAATTAGATG-GTATTCTAGTTTAGAGGTGATTCGGCGCTGCGGTGCGACTG
China3 MH165180     GGGTCTGTCGGAAACCATTATTTATGAAGTGACTAGCAATCATGGATTAAATTAGATGTGTATTCTAGTTTAGAGGCGATTCGGCGCTGCGGTGCGACTG
DWV-NVN             AGGTCTGTCGGAAACCATTATTTATGAAGTGACTAGCAATCATGGATTAAATTAGATG-GTATTCTAATTTAGAGGCGATTCGGCGCTGCGGTGCGACTG
DWV-SVN             AGGTCTGTCGGAAACCATTATTTATGAAGTGACTAGCAATCATGGATTAAATTAGATG-GTATTCTAATTTAGAGGCGATTCGGCGCTGCGGTGCGACTG
```

```
                             710       720       730       740       750       760       770       780       790       800
                    ....|....|....|....|....|....|....|....|....|....|....|....|....|....|....|....|....|....|....|....|
Japan AB070959      AAACTTCTAAATTAGCATGTCAGATTGTATTATGAATGCGTTAGTAGTAATTTCTGCGATAGAGCTGGGACCCCTCAGTCTCTCAGGTATTGTATGAGGC
USA1 AY291384       AAACTTCTAAATTAGCATGTCAGATTGTATTATGAATGCGTTAGTAGTAATTTCTGCGATAGAGCTGGGACCCCTCAGTCTCTCAGGTATTGTACGAGGC
Chile JQ413340      AAACTTCTAAGTTAGCATGTCAGATTATATTATGAATGCGTTAGTAGTAATTTCTGCGATAGAGCTGGGACCCCTCAGTCTCTCAGGTATTGTACGAGGC
Italy1 AJ489744     AAACTTCTAAATTAGCATGTCAGATTATATTATGAATGCGTTAGTAGTAATTTCTGCGATAGAGCTGGGACCCCTCAGTCTCTCAGGTATTGTACGAGGC
France KX373899     AAACTTCTAAATTAGCATGTCAGATTATATTATGAATGCGTTAGTAGGAATTTCTGCGATAGAGCTGGGACCCCTCAGTCTCTCAGGTATTGTATGAGGC
UK1 GU109335        AAACTTCTAAATTAGCATGTCAGGTTATATTATGAATGCGTTAGTAGTAATTTCTGCGATAGAGCTGGGACCCCTCAGTCTCTCAGGTATTGTATGAGGC
UK2 KJ437447        AAACTTCTAAATTAGCATGTCAGGTTATATTATGAATGCGTTAGTAGTAATTTCTGCGATAGAGCTGGGACCCCTCAGTCTCTCAGGTATTGTATGAGGC
Korea1 JX875304     AAACTTCTAAATTAGCATGTCAGATTATATTATGAATGCGTTAGTAGTAATTTCTGCGATAGAGCTGGGACCCCTCAGTCTCTCAGGTATTGTATGCGGC
Korea2 JX878305     AAACTTCTAAATTAGCATGTCAGATTATATTATGAATGCGTTAGTAGTAATTTCTGCGATAGAGCTGGGACCCCTCAGTCTCTCAGGTATTGTATGAGGC
China1 MF770715     AAACCTCTAAATTAGCATGTCAGATTATATTATGAATGCGTTAGTAGTAATTTCTGCGATAGAGCTGGGACCCCTCAGTCTCTCAGGTATTGTATGCGGC
China2 MF036686     AAACTTCTAAATTAGCATGTCAGATTATATTATGAATGCGTTAGTAGTAATTTCTGCGATAGAGCTGGGACCCCTCAGTCTCTCAGGTATTGTATGAGGC
China3 MH165180     AAACTCCTAAACTAGCATGTCAGATTGTATTATGAATGCGCTAGTAGTAATTTCTGCGATAGAGCTGGGACCCCTCAGTCTCTCAGGTATTGTATGAGGC
DWV-NVN             AAACTTCTAAATTAGCATGTCAGATTATATTATGAATGCGTTAGTAGTAATTTCTGCGATAGAGCTGGGACCCCTCAGTCTCTCAGGTATTGTATGAGGC
DWV-SVN             AAACTTCTAACTTAGCATGTCAGATTATATTATGAATGCGTTAATAGTAATTTCTGCGATAGAGCTGGGACCCCTCAGTCTCTCAGGTATTGTATGAGGC
```

```
                          810       820       830       840       850       860       870       880       890       900
                 ....|....|....|....|....|....|....|....|....|....|....|....|....|....|....|....|....|....|....|....|
Japan AB070959   GAAAGTGTGAAAGTTTTGTATATGTTTTTATATATACGACTGTATCGGGAATTCCTTTAGCAAGAATCCTTTTAATACAGTATAATTTGTGCTACGGTAC
USA1 AY291384    GAAAGTGTGAAAGTTTTGTATGTGTTTTTATATGTACGACTGTATCGGGAATTCCTTTAGCAAGAATCCTTTTAATACAGTATAATCTGTGCTACGGTAC
Chile JQ413340   GAAAGTGTGAAAGTTTTGTATGTGTTTTTATATGTACGACTGTATCGGGACTTCCTTTAGCAAGAATCCTTTTAATACAGTATAATTTGTGCTACGGTAC
Italy1 AJ489744  GAAAGTGTGAAAGTTTTGTATGTGTTTTTATATGTACGACTGTATCGGGAATTCCTTTAGCAAGAATCCTTTTAATACAGTATAATTTGTGCTACGGTAC
France KX373899  GAAAGTGTGAAAGTTTTGTATGTATTTTTATATGTACGACTGTATCGGGAATTCCTTTAGCAAGAATCCTTTTAATACAGGATAATTTGTGCTACGGTAC
UK1 GU109335     GAAAGTGTGAAAGTTTTGTATGTGTTTTTATATGTACGACTGTATCGGGAATTCCTTTAGCAAGAATCCTTTTAATACAGTATAATCTGTGCTACGGTAC
UK2 KJ437447     GAAAGTGTGAAAGTTTTGTATGTGTTTTTATATGTACGACTGTATCGGGAATTCCTTTAGCAAGAATCCTTTTAATACAGTATAATCTGTGCTACGGTAC
Korea1 JX875304  GAAAGTGTGAAAGTTTTGTAGGTGTTTTTATATATGTCACAGTATCGGGAATTCCTTTAGCAAGAAACCTTTTAATACAGTATAATTTGAGCTACGGTAC
Korea2 JX878305  GAAAGTGTGAAAGTTTTGTAGGTGTTTTTATATGTACGACTGTATCGGGAATTCCTTTAGCAAGAATCCTTTTAATACAGTATAATTTGTGCTACGGTAC
China1 MF770715  GAAAGTGTGAAAGTTTTGTAGGTGTTTTCATAGGTACGACTGTATCGGGAACTCCTTTAGCAAGAATCCTTTTAATACAGTATAATTTGTGCTACGGTAC
China2 MF036686  GAAAGTGTGAAAGTTT-GTAGGTGTTCTTATATGTACGACTGTATCGGGAATTCCTTTAGCAAGAATCCTTTCAATGCAGTATAATTTGTGCTACGGTAC
China3 MH165180  GAAAGTGTGAAAGTTTTGTATGTATTTTTATATGTACGACTGTATCGGGACTTCCTTTAGCAAGAATCCTTTTAATACAGTATAATTTGTGCTACGGTAC
DWV-NVN          GAAAGTGTGAAAGTTTTGTATGTGTTTTTGTACGTACGACTGTATCGGGATTTCCTTTAGCAAGAATCCTTTTAATACAGTATAATTTGTGCTACGGTAC
DWV-SVN          GAAAGTGTGAAAGTTTTGTATGTGTTTTTGTACGTACGACTGTATCGGGATTTCCTTTAGCAAGAATCCTTTTAATACAGTATAATTTGTGCTACGGTAC

                          910       920       930       940       950       960       970       980       990      1000
                 ....|....|....|....|....|....|....|....|....|....|....|....|....|....|....|....|....|....|....|....|
Japan AB070959   GTTACGTTCGCAGGGCACCCGTTAATGTCTCATAGCCCAGACGATGGCGGATGGAAAGACATCATATTTTATTTTAATGCTGTCTTTATTGCTGATTTAT
USA1 AY291384    GTTACGTTCGCAGGGCACCCGTTAATGTCTCATAGCCCAGACGATGGCGGATGGAAAGACATCATATTTTATTTTAATGCTGTCTTTATTGCTGATTTAT
Chile JQ413340   GTTACGTTCGCAGGGCACCCGTTAATGTCTCATAGCCCAGACGATGACGGATGGAAAGACATCATATTTTATTTTAATGCTGTCTTTATTGCTGATTTAT
Italy1 AJ489744  GTTACGTTCGCAGGGCACCCGTTAATGTCTCATAGCCCAGACGATGGCGGATGGAAAGACATCATATTTTATTTTAATGCTGTCTTTATTGCTGATTTAT
France KX373899  GTTACGTTCGCAGGGCACCCGTTAATGTCTCATAGCCCAGACGATGGCGGATGGAAAGACATCATATTTTATTTTAATGCTGTCTTTAATGCTGATTTAT
UK1 GU109335     GTTACGTTCGCAGGGCACCCGTTAATGTCTCATAGCCCAGACGATGGCGGATGGAAAGACATCATATTTTATTTTAATGCTGTCTTTATTGCTGATTTAT
UK2 KJ437447     GTTACGTTCGCAGGGCACCCGTTAATGTCTCATAGCCCAGACGATGGCGGATGGAAAGACATCATATTTTATTTTAATGCTGTCTTTATTGCTGATTTAT
Korea1 JX875304  GTTACGTTCGCAGGGCTCCCGTTAAGGTCCCATAGCCCAGACAATGGCGGATGGAAAGACATCATATTTTATTTTAATGCGGTCTTTATGGCTGATTTAT
Korea2 JX878305  GTTACGTTCGCAGGGCACCCGTTAATGTCTCATAGCCCAGACGATGGCGGATGGAAAGACATCATATTTTATTTTAAAGTCGGCATTATTGCTGAATTAT
China1 MF770715  GTTACGTTCGCAGGGCACCCGTTAATGTCTCATGGCCCAGACGATGGCGGATGGAAAGACATCATATTTTATTTTAATGCTGTCTTTATTGCTGATTTAT
China2 MF036686  GTTACGTTCGCAGGGCACCCGTTAATGTCTCATAGCCCAGACGATGGCGGATGGAAAGACATCATATTTTATTTTAATGCTGTTTGTATCGCTGATTTAT
China3 MH165180  GTTACGTTCGCAGGGCACCCGTTAATGTCTCATAGCCCAGACGATGGCGGATGGAGAGACATCATATTTTATTTTAATGCTGTCTTTATTGCTGATTTAT
DWV-NVN          GTTACGTTCGCAGGGCACCCGTTAATGTCTCATAGCCCAGACGATGGCGGATGGAAAGACATCATTTTTCTTTTTAATGCTGCCTTTATTGCTGATTTAT
DWV-SVN          GTTACGTTCGCAGGGCACCCGTTAATGTCTCATAGCCCAGACGATGGCGGATGGAAAGACATCATTTTTAAATTTAATGCTGTCTTTATTGCTGATTTAT

                         1010      1020      1030      1040      1050      1060      1070      1080      1090      1100
                 ....|....|....|....|....|....|....|....|....|....|....|....|....|....|....|....|....|....|....|....|
Japan AB070959   TTTGCTGTTTTTATTTGCTATTTTATATTTGCTAATTTTCATTATTGCGAAATATATTACATTGCTATTTTTATTATATACGCTAGATTCAATTTTATTT
USA1 AY291384    CTTGCTGTTTTTATTTGCTATTTTATATTTGCTAATTTTCATTATTGCGAAATATATTACATTGCTATTTTTATTATATACGCTAGATTCAATTTTATTT
Chile JQ413340   TTTGCTGTTTTTATTTGCTATTTTATATTTGCTGATTTTCATTATTGCGAAATATATTACATTGCTATTTTTATTATACACGCTAGATTCAATTTTATTT
Italy1 AJ489744  TTTGCTGTTTTTATTTGCTATTTTATATTTGCTAATTYTCATTATTGCGAAATATATTACATTGCTATTTTTATTATATACGCTAGATTCAATTTTATTT
France KX373899  CTTGCTGTTTTTATTTGCTATTTTATATTTGCTAATTTTCATTATTGCGAAATATATTACATTGCTATTTTTATTATATACGCTAGATTCAATTTTATTT
UK1 GU109335     TTTGCTGTTTTTATTTGCTATTTTATATTTGCTAATTTCCATTATTGCGAAATATATTACATTGCTATTTTTATTATATACGCTAGATTCAATTTTATTT
UK2 KJ437447     TTTGCTGTTTTTATTTGCTATTTTATATTTGCTAATTTTCATTATTGCGAAATATATTACATTGCTATTTTTATTATATACGCTAGATTCAATTTTATTT
Korea1 JX875304  CTTGCTGTTTTTATTTGCTATTTTATATTTGCTAATTTTCATTATAGCTAATTATATCGCATTGCTATTTTTATTATTTACGCTAGATTCAATTTTATTT
Korea2 JX878305  TTTGCTGTTTTTATTTGCAATCTTAAATTTGCCAATTTTCATTATTGCGAATTAAATTGCCTTGCTATTTTTATTATTTACGCCAGATTCAATTTTATTT
China1 MF770715  TTTGCTGTTTTCATTTGCTATTTTATATTTGCTAATTTTCATTATTGCTAAATATATTATATTGCTATTTTTATTGTATACGCTAGATTCAATTTTATTT
China2 MF036686  TTTGCTGTCTTTATTTGCTATCTTATATTTGCTAATTTTCAATATTGCTAACTATATTGCATTGCTATTTTTATTATTTACGCTAGACTTAATTTTATTA
China3 MH165180  TTTGCTGTTTTCATTTGCTATTTTATATTTGCTAATTTTCATTATTGCTAAATATATTACATTGCTATTTTTATTATATACGCTAGATTCAATTTTATTA
DWV-NVN          TTTGCTGCTTTTATTTGCTACTCTATATTTGCTAATTTTCATTATTGCGACATATATTACATTGCTATTTTTATTATATACGCTAGATTCAATTTTATTT
DWV-SVN          TTTGCTGTTTTCATTTGCTATTTTATATTTGCTAATTTTCATTATTGCGACATATATTACATTGCTATTTTTATTATATACGCTAGATTCGATTTTATTT

                         1110      1120      1130      1140      1150      1160      1170      1180      1190      1200
                 ....|....|....|....|....|....|....|....|....|....|....|....|....|....|....|....|....|....|....|....|
Japan AB070959   ATCCTATATTTTCAATTTAATTTTGATTTTGAAGGTAAATATATATAATTAATTATTAGAAATGGCCTTTAGTTGTGGAACTCTCTCTTACTCTGCCGTC
USA1 AY291384    TTTCTATATTTTCAATTTAATTTTGATTTCGAAGGTAAATATATATAATTAATTATTAAAAATGGCCTTTAGTTGTGGAACTCTTTCTTACTCTGCCGTC
Chile JQ413340   TTCCTATATTTTCAATTTAATTTTGATTTTGAAGGTAAATATATATAATTAATTATTAAAAATGGCCTTTAGCTGTGGAACTCTTTCTTACTCTGCCGTC
Italy1 AJ489744  TTYCTATATTTTCAATTTAATTTTGATTTCGAAGGTAAATATATATAATTRATTATTAAAAATGGCCTTTAGTTGTGGAACTCTYTCTTACTCTGCCGTC
France KX373899  TTCCTATATTTTCAATTTAATTTTGATTTCGAAGGTAAATATATATAATTAATTATTAAAAATGGCCTTTAGCTGTGGAACTCTTTCTTACTCTGCTGTC
UK1 GU109335     TTTCTATATTTTCAATTTAATTTTGATTTCGAAGGTAAATATATATAATTAATTATTAAAAATGGCCTTTAGTTGTGGAACTCTTTCTTACTCTGCCGTC
UK2 KJ437447     TTTCTATATTTTCAATTTAATTTTGATTTCGAAGGTAAATATATATAATTAATTATTAAAAATGGCCTTTAGTTGTGGAACTCTTTCTTACTCTGCCGTT
Korea1 JX875304  ACTCTATATTTCCAATTTAATTTTGATTTTGAAGGTAAATATATATAATTGATTACTAACAATGGCCTTTAGCTGCGGAACTCTTTCTTACTCTGCCGCC
Korea2 JX878305  ATCCTATATTTTCAATTTAATTTTGATTTTGAAGGGAAATATATATAATTGATTATTAACAATGGCCTTTAGCTGCGGAACCCTTTCTTACTCTGCCGTC
China1 MF770715  ATCCTATATTTTCAATTTGATTTTGATTTTGAAGGTAAATATATATAATTAAATACAAAAAATGGCTTTTAGTTGTGGAACTCTCTCCTACTCTGCCGTT
China2 MF036686  ATCCTATATTTTCAATTTAATTTTGATTTTGAAGGTAAATATATATAATTAATTACTAAAAATGGCCTTTAGTTGTGGAACTCTCTCTTATTCTGCCGTT
China3 MH165180  ATCCTATATTTTCAATTTAATTTTGATTTTGAAGGTAAATATATATAATTAATTTCTAAAAATGGCCTTTAGTTGTGGAACTCTCTCTTACTCTGCCGTT
DWV-NVN          ATCTTATATTTTCAATTTGATTTTGATTTTGAAGGTAAATATATATAATAACTTA-GAAAAATGGCTTTTAGTTGTGGAACCCTCTCTTACTCTGCTGTC
DWV-SVN          ATCCTATATTTTCAATTTGATTTTGATTTTGAAGGAAAATATATATAATAAATTA-TAAAAATGGCTTTTAGTTGTGGAACTCTCTCTTACTCTGCTGTC
```

```
                         1210      1220      1230      1240      1250      1260      1270      1280      1290      1300
                    ....|....|....|....|....|....|....|....|....|....|....|....|....|....|....|....|....|....|....|....|
Japan AB070959      ACCCAAGCTCCGTCTGTCGCCTATGCACCTCGTACATGGGAAGTTGATGAAGCTAGGCGGCGCCGAGTTATTAAACGTTTGGCGCTGGAGCAAGAACGTA
USA1 AY291384       GCCCAAGCTCCGTCTGTCGCCTATGCACCTCGTACATGGGAAGTTGATGAAGCTAGGCGGCGCCGAGTCATTAAACGTTTGGCGCTGGAGCAAGAACGTA
Chile JQ413340      GCCCAAGCTCCGTCTGTCGCCCATGCACCTCGTACATGGGAAGTTGATGAAGCTAGGCGGCGCCGAGTCATTAAACGTTTGGCGCTGGAGCAAGAACGTA
Italy1 AJ489744     GCCCAAGCTCCGTCTGTCGCYTATGCACCTCGTACATGGGAAGTTGATGAAGCTAGGCGGCGCCGAGTCATTAAACGTTTGGCGCTGGAGCAAGAACGTA
France KX373899     GCCCAAGCTCCGTCCGTCGCCCATGCACCTCGTACATGGGAAGTTGATGAAGCTAGGCGGCGCCGAGTCATTAAACGTTTGGCGCTGGAGCAAGAACGTA
UK1 GU109335        GCCCAAGCTCCGTCTGTTGCCCATGCACCTCGTACATGGGAAGTTGATGAAGCTAGGCGGCGCCGAGTCATTAAACGTTTGGCGCTGGAGCAAGAACGTA
UK2 KJ437447        GCCCAAGCTCCGTCTGTCGCCTATGCACCTCGTACATGGGAAGTTGATGAAGCTAGGCGGCGCCGAGTCATTAAACGTTTGGCGCTGGAGCAAGAACGTA
Korea1 JX875304     GCCCAAGCCCCGTCTGTCGCCCATGCACCTCGTACATGGGAAGTTGATGAAGCTAGGCGGCGCCCAGTTATTAAACGTTTGGCGCTGGAGCAAGAACGTA
Korea2 JX878305     GCCCAAGCCCCGTCTGGCGCCCATGCTCCTCGTACATGGGAAGTTGATGAAGCTAGGCGGCGCCGAGTTATTAAACGTTTGGCGCTGGAGCAAGAACGTA
China1 MF770715     GCCCAAGCTCCGTCTGTTGCCCACGCTCCTCGTACATGGGAGGTTGATGAAGCTAGGCGGCGCCGAGTCATTAAACGTTTGGCGTTAGAGCAAGAACGTA
China2 MF036686     GCCCAAGCTCCGTCTGTCGCCCATGCTCCTCGTACATGGGAAGTTGATGAAGCTAGGCGGCGCCGAGTTATTAAACGTTTGGCGCTGGAGCAAGAACGTA
China3 MH165180     GCCCAAGCTCCGTCTGTCGCCCATGCTCCTCGTACATGGGAAGTTGATGAAGCTAGGCGGCGCCGAGTTATTAAACGTTTAGCACTGGAGCAAGAACGTA
DWV-NVN             GCCCAGGCTCCGTCCGTCGCCCATGCACCCCGTACGTGGGAAATTGATGAAGCTAGGCGACGCCGCGTAATTAAGCGGTTGGCGCTGGAACAAGAACGTA
DWV-SVN             GCCCAGGCTCCGTCTGTCGCCCATGCACCCCGTATGTGGGAAATTGATGAAGCTAGGCGACGCCGCGTAATTAAGCGGTTGGCGCTGGAACAAGAACGTA

                         1310      1320      1330      1340      1350      1360      1370      1380      1390      1400
                    ....|....|....|....|....|....|....|....|....|....|....|....|....|....|....|....|....|....|....|....|
Japan AB070959      TTCGTAATGTTCTTGACGTTGACGTCTATAACCAGGCGACATGGGAACAGGAAGACGTGCGCGATAACGAGTTCCTAACGGAACAACTAAACAATTTATA
USA1 AY291384       TTCGTAACGTTCTTGACGTTGGCGTCTATGACCAGGCGACATGGGAACAGGAGGACGCGCGCGATAATGAGTTCCTAACGGAACAATTAAACAATTTATA
Chile JQ413340      TGCGTAACGTTCTTGACGCTGACGTCTATGCCCAGGCGACATGGGAACAGGAGGACGCGCGCGATAATGAGTCCCTAACGGAACAATTAAATAATTTATA
Italy1 AJ489744     TTCGTAACGTTCTTGACGTTGCCGTCTATGACCAGGCGACATGGGAACAGGAGGACGCGCGCGATAATGAGTTCCTAACGGAACAATTAAAYAATTTATA
France KX373899     TTCGTAACGTTCTTGACGCTGACGTCTATGGCCAGGCGACGTGGGAACAGGAGGACGCGCGCGATAATGAGTTCCTAACGGAACAATTAAACAATTTATA
UK1 GU109335        TTCGTAACGTTCTTGACGTTGACGTCTATGCCCAGGCAACATGGGAACAGGAGGACGCGCGCGATAATGAGTTCCTAATGGAACAATTAAACAATTTATA
UK2 KJ437447        TTCGTAACGTTCTTGACGTTGATGTCTATGCCCAGACGACATGGGAACAAGAGGACGCGCGGGATAATGAGTTCCTAACGGAACAATTAAACAATTTATA
Korea1 JX875304     TGCGTAACGTTCGTGACGTTGACGTCTATGACCAGGCGACATGGGAACAGGAGGACGCGCGCGATAATGAATTCCTAATGGAACAATTAAATAATTTATA
Korea2 JX878305     TGCGTAACGTTCTGGACGTTGACGTCTATAACCAGACGACATGGGAACAGGAGGACGCGCGCGACAATGAGTTCCTAATGGAACAATTAAATAATTTATA
China1 MF770715     TGCGTAATGTTCTTGACGCTGACGTTTATTACCAGGCGACATGGGAACAGGAGGACGCGCGTGACAATGAGTTCCTAATGGAACAATTAAATAATTTATA
China2 MF036686     TGCGTAACGTTCTTGACGCTGACGTTTATTACCAGGCGACATGGGAACAGGAGGATGCGCGCGACAATGAGTTCCTAATGGAACAATTAAATAATTTATA
China3 MH165180     TGCGTAACGTTCTGGACGTTGATGTCTATAACCAGGCGACATGGGAACAGGAGGACGCGCGCGATAATGAGTTCCTAATGGAACAATTAAATAATTTATA
DWV-NVN             TGCGTAACGTTCTTGACGCTGGAGTTTATGACCAGGCGACATGGGAACAGGAAGACGCGCGAGACAACGAGTTCCTAATGGAACAATTAAATAATTTATA
DWV-SVN             TGCGTAACGTTCTTGATGCTGATGTTTATGACCAGGCGACATGGGAACAGGAAGACGCGCGAGACAACGAGTTCCTAATGGAACAATTAAATAACTTATA

                         1410      1420      1430      1440      1450      1460      1470      1480      1490      1500
                    ....|....|....|....|....|....|....|....|....|....|....|....|....|....|....|....|....|....|....|....|
Japan AB070959      TACTATTTATTCCATCGCTGAACGTTGTACGCGCCGGCCTATCAAAGAGTGCTCTCCCATATCAGTTTCGAATAGGTTCGCCCCATTGGAATCCCTTAAG
USA1 AY291384       TACTATTTATTCGATCGCTGAACGTTGTACGCGTCGGCCTATCAAAGAGTACTCTCCTATATCAGTTTCGAATAGGTTTGCTCCACTGGAATCCCTCAAG
Chile JQ413340      TACTATTTATTCGATCGCTGAACGTTGTACGCGTCGGCCTATCAAAGAGCACTCTCCTATATCAGTTTCGAATAGGTTTGCTCCATTGGAATCCCTTAAG
Italy1 AJ489744     TACTATTTATTCGATCGCTGAACGTTGTACGCGTCGGCCTATCAAAGAGYACTCTCCTATATCAGTTTCGAATAGGTTTGCTCCACTGGARTCCCTYAAG
France KX373899     TATTATTTATTCGATCGCTGAACGTTGTACGCGTCGGCCTATCAAAGAGCACTCTCCTATATCAGTTTCGAATAGGTTTGCTCCACTGGAATCCCTCGAG
UK1 GU109335        TACTATTTATTCGATCGCTGAACGTTGTACGCGTCGGCCTATCAAAGAGTACTCGCCTATATCAGTTTCGAATAGGTTTGCTCCACTGGAATCCCTCAAG
UK2 KJ437447        TACTATTTATTCGATCGCTGAACGTTGTACGCGTCGGCCCATCAAAGAGCACTCTCCTATATCAGTTTTGAATAGGTTTGCTCCACTGGAATCTCTCAAG
Korea1 JX875304     TACTATTTATTCGATCGCTGAACGTTGTGCGCGTCGACCTATCAAAGAGCACTCTCCTATATCAGTTTCGAATAGATTTGCCCCACTGGAATCCCTCAAG
Korea2 JX878305     TACTATTTATTCGATCGCTGAACGTTGTACGCGTCGGCCTATCAAAGAACATTCTCCTATATCGATTTCGAATAGGTTTGTCCCATTGGAATCCCTCAAG
China1 MF770715     TACTATTTATTCCATTGCTGAGCGTTGTACGCGTCGGCCTATTAAAGAGTGCTCTCCTATATCAGTTTCGAATAGGTTTGCCCCATTGGAATCTCTCAAG
China2 MF036686     TACTATTTATTCCATTGCTGAGCGTTGTACGCGTCGGCCTATTAAAGAGTATTCTCCTATATCAGTTTCGAATAGGTTTACCCCATTGGAATCTCTCAAG
China3 MH165180     TACTATTTATTCGATCGCTGAACGTTGTACGCGTCGGCCCATCAAAGAACATTCTCCTATATCAGTTTCGAATAGGTTTACCCCATTGGAATTTCTCAAG
DWV-NVN             TACTATTTATTCGATAGCTGAACGTTGTACGCGCCGACCTATTAAAGAACATACTCCTATATCAGTTACGAACCGGTTTTCTCCATTGGAATCCCTTAAG
DWV-SVN             TACTATTTATTCGATAGCTGAACGTTGTACGCGCCGACCTATTAAAGAACATACTCCTATATCAGTTACGAACCGGTTTTCTCCATTGGAATTCCTTAAG

                         1510      1520      1530      1540      1550      1560      1570      1580      1590      1600
                    ....|....|....|....|....|....|....|....|....|....|....|....|....|....|....|....|....|....|....|....|
Japan AB070959      GTCGAAATTGGTCAAGAAGCAAGCGAATGTATATTTAAGAAACCTAAATATACGCGCGTTTGCAAGAAAGTGAAGCGTGTTGCAACTCGCTTCGTTCGCG
USA1 AY291384       GTCGAGGTCGGTCAAGAAGCAGGCGAATGTATATTTAAGAAACCTAAATATACGCGCGTTTGCAAGAAAGTGAAGCGTGTTGCAACTCGCTTCGTTCGTG
Chile JQ413340      GTCGAGGTCGGTCAAGAGGCAGGCGAATATATATTTAAGAAACCTAAATATACGCGCGTTTGCAAGAAAGTGAAGCGTGTTGCAACTCGCTTCGTTCGTG
Italy1 AJ489744     GTCGAGGTCGGTCAAGAAGCARGCGAATGTATRTTTAAGAAACCTAAATATACGCGCRTTTGCAAGAAAGTGAAGCGTGTTGCAACTCGCTTCGTTCGTG
France KX373899     GTCGAGGTCGGTCAAGAAGCAGGCGAATGTATATTTAAGAAACCTAAATATACGCGCGTTTGCAAAAAAGTGAAGCGTGTTGCAACTCGCTTCGTTCGTG
UK1 GU109335        GTCGAGGTTGGTCAAGAAGCAAGCGAATGTATATTTAAGAAACCTAAATATACGCGCGTTTGCAAGAAAGTGAAGCGTGTTGCAACTCGCCTCGTTCGTG
UK2 KJ437447        GTCGAGGTCGGTCAAGAAGCAGGCGAATGTATATTTAAGAAACCTAAATATACGCGCGTTTGCAAGAAAGTGAAGCGCGTTGCAACTCGCTTCGTTCGTG
Korea1 JX875304     GTCGAAGTCGGTCAAGAAGCAAGCGAATGTATATTTAAGAAACCTAAATATACGCGCGTTTGCAAGAAAGTGAAGCGTGTTGCAACTCGTTTCGTTCGCG
Korea2 JX878305     GTTGAAGTCGGTCAAGAAGCAGGCGAATGTTTATTTAAGAAACCTAAATATACGCGCGTCTGCAAGAAAGTGAAGCGTGTTGCAACTCGCTTTGTTCGTG
China1 MF770715     GTCGAAGTCGGTCAAGAAGCAAGCGAATGTATGTTTAAGAAACCTAAATATACGCGCGTTTGCAAGAAAGTGAAGCGTGTTACAACTCGCTTCGTTCGTG
China2 MF036686     GTCGAAGTCGGTCAAGAAGCAAGCGAACGTATATTTAAGAAACCTAAATATACGCGCGTTTGCAAGAAAGTGAAGCGCGTTGCAACTCGCTTCGTTCGTG
China3 MH165180     GTCGAAGTCGGTCAAGAAGCAAGCGAATGTTTATTTAAGAAACCTAAATATACGCGCATTTGTAAGAAAGTGAAGCGTGTCGCAACTCGCTTCGTTTGTG
DWV-NVN             GTCGAAGTCGGTCAAGAAGTAGGCGAATTTATATTTAAGAAACCTAAATATACACGCGTCTGCAAGAAGGTAAAGCGAGTCGCGACCCGCTTTGTTCGTG
DWV-SVN             GTCGAAGTTGGTCAAGAAGTAGGCGAATTTATATTTAAGAAACCTAAATATACACGCGTCTGTAAGAAAGTAAAGCGAGTCGCGACCCGCTTTGTTCGTG
```

```
                        1610      1620      1630      1640      1650      1660      1670      1680      1690      1700
                    ....|....|....|....|....|....|....|....|....|....|....|....|....|....|....|....|....|....|....|....|
Japan AB070959      AAAAAGTTGTTCGTCCTATGTGTCCCAGATCCCCTATGCTATTGTTTAAGCTTAAGAAAATTATTTATGATTTGCATTTATATAGGTTAAGAAAACAGAT
USA1 AY291384       AAAAAGTTGTTCGTCCTATGTGTTCTAGATCCCCTATGCTATTATTTAAGCTTAAGAAAATTATTTATGATTTGCACTTATATAGATTAAGAAAACAGAT
Chile JQ413340      AAAAAGTTGTTCGTCCTATGTGTTCTAGATCCCCTATGCTATTATTTAAGCTTAAGAAAATTATTTATGATTTGCATTTATATAGATTAAGAAAGCAGAT
Italy1 AJ489744     AAAAAGTTGTTCGTCCTATGTGYTCWAGATCCCCTATGCTATTATTTAAGCTTAAGAAAATTATTTATGATTTGCATTTATATAGATTAAGAAAACAGAT
France KX373899     AAAAAGTTGTTCGTCCTATGTGTTCTAGATCCCCTATGCTATTATTTAAGCTTAAGAAAGTTATTTATGATTTGCATTTATATAGATTAAGAAAACAGAT
UK1 GU109335        AAAAAGTTGTTCGTCCTATGTGTTCTAGATCCCCTATGCTATTATTTAAGCTTAAGAAAGTTATTTATGATTTGCATTTATATAGATTAAGAAAACAGAT
UK2 KJ437447        AAAAAGTCGTTCGTCCTATGTGTTCTAGATCCCCTATGCTCTTATTTAAGCTTAAGAAAATTATTTATGATTTGCACTTATATAGATTAAGAAAACAGAT
Korea1 JX875304     AAAAAGTTGTTCGTCCCATGTGTCCTAGATCCCCTATGTTATTGTTTAAGCTTAAGAAAATTATTTATGATTTACATTTATATAGGTTAAGAAAACAGAT
Korea2 JX878305     AAAAAATTGTGCGTCCTATGTGTTCTAGATCCCCTATGCTATTATTTAAGCTTAAGAAAATTATTTATGATTTGCATTTATATAGGTTAAGAAAACAGAT
China1 MF770715     AAAAAGTTGTTCGTCCCATGTGTTCCAGATCCCCTATGTTATTATTTAAACTTAAGAAAATTATTTATGATTTGCATTTGTATAGGTTAAGAAAACAGAT
China2 MF036686     AAAAAGTCGTTCGTCCTATGTGTTCCAGATCTCCTATGTTATTATTTAAACTTAAGAAAATTATTTATGACTTGCATTTATATAGGTTAAGAAAACAGAT
China3 MH165180     AAAAAGTCGTTCGTCCTATGTGTTCCAGATCTCCTATGCTATTATTTAAGCTTAAGAAAATTATTTATGATTTGCATTTATATAGGTTAAGAAAACAGAT
DWV-NVN             AGAAAGTTGTTCGTCCTACGTGTAGTAGATCTCCTATGTTATTGTTTAAACTTAAGAAAATTATTTATGATTTTCATTTATTTAGATTAAGAAAACAAAT
DWV-SVN             AGAAAGTTGTTCGTTCTACGTGTAGTAGATCTCCTATGTTGTTGTTTAAACTTAAGAAAATTATTTATGATTTTCATTTATTTAGATTAAGAAAACAAAT
```

```
                        1710      1720      1730      1740      1750      1760      1770      1780      1790      1800
                    ....|....|....|....|....|....|....|....|....|....|....|....|....|....|....|....|....|....|....|....|
Japan AB070959      TAGGATGTTGAGACGTCAAAAACAGCGCGATTATGAATTAGAGTGTGTCACTAATCTGTTACAATTATCGAATCCGGTGCAGGCAAAACCAGAGATGGAT
USA1 AY291384       TAGGATGTTGAGACGTCAAAAACAGCGCGATTACGAGTTAGAGTGTGTCACTAATCTGTTACAATTATCGAATCCGGTGCAGGCAAAACCAGAGATGGAT
Chile JQ413340      TAGGATGTTGAGACGTCAAAAACAGCGCGAGTATGAGTTAGAGTGTGTCACTAATCTGTTACAATTATCGAATCCGGTGCAGGCAAAACCAGAGATGGAT
Italy1 AJ489744     TAGGATGTTGAGACGTCAAAAACAGCGCGATTATGAGTTAGAGTGTGTCACTAATCTGYTACAATTATCGAATCCAGTGCAGGCAAAACCAGAGATGGAT
France KX373899     TAGGATGTTGAGACGTCAAAAACAGCGCGATTATGAGTTAGAGTGTGTCACTAATCTATTACAATTATCGAATCCAGTGCAGGCAAAACCAGAGATGGAT
UK1 GU109335        TAGGATGTTGAGACGTCAAAAACAGCGCGATTATGAGTTAGAATGTGTCACTAATCTGTTACAATTATCGAATCCGGTGCAGGCAAAGCCAGAGATGGAT
UK2 KJ437447        TAGGCTTCTCAGACGCGAAAAACAGCGTGAATACGAGTTAGAGTGTGTTACTAGTTTGCTACAGCTATCTAATCCTGTTTCAGCTAAACCTGAGATGGAC
Korea1 JX875304     TAGGCTGTTGAGACGCCAGAAACAGCGTGAGTATGAGTTAGAGTGCGTCACTAGTCTGTTACAATTATCGGATCCGGTGCAGGCAAAACCAGAGATGGAT
Korea2 JX878305     TAGGATGTTGAGACGTCAAAAACAGCGCGATTATGAGTTAGAGTGTGTCACTAATCTGTTACAATTATCGAATCCGGTGCAGGCAAAGCCAGAGATGGAT
China1 MF770715     TAGGCTGTTGAGACGCCAAAAACAGCGTGAGTATGAGTTAGAGTGCGTCACTAGTCGGTTACAATTATCGGATCCGGTGCAGGCAAAACCAGAGATGGAT
China2 MF036686     TAGGTTGTTGAGACGCCAAAAACAGCGTGAGTATGAGTTAGAGTGCGTCACTAGTCTGTTACAATTATCGGATCCAGTGCAGGCAAAACCAGAGATGGAT
China3 MH165180     TAGGCTGTTGAGACGCCAAAAACAGCGTGAGTATGAGTTAGAGTGCGTCACTAGTCTGTTACAATTATTAGATCCGGTGCAGGCAAAACCAGAGATGGAT
DWV-NVN             TAGGATATTAAGGCGTCAGAAGCAGCGTGATTATGAGTTAGAATGTGTTACCAGTCTATTACAATTGGCAAATCCGGTACAGGCGAAACCAGAAATGGAT
DWV-SVN             TAGGATATTAAGGCGTCAGAAGCAGCGGGATTATGAGTTAGAGTGTGTTACCAGTCTATTACAATTGGCAAATCCGGTACAGGCGAAACCGGAAATGGAT
```

```
                        1810      1820      1830      1840      1850      1860      1870      1880      1890      1900
                    ....|....|....|....|....|....|....|....|....|....|....|....|....|....|....|....|....|....|....|....|
Japan AB070959      AACCCTAATCCAGGACCTGATGGCGAGGGTGAAGTTGAATTAGAAAAAGATAGTAACGTTGTTTTAACAACTCAGCGAGATCCTAGTACATCTATTCCAG
USA1 AY291384       AACCCTAATCCAGGACCTGATGGCGAGGGTGAAGTTGAATTAGAAAAGGATAGTAATGTTGTTTTAACAACTCAGCGAGATCCTAGTACATCTATTCCAG
Chile JQ413340      AACCCTAATCCAGGACCTGATGGCGAGGGTGAAGTTGAATTAGAAAAGGATAGTAATGTTGTTTTAACAACTCAGCGAGATCCTAGTACATCTATTCCAG
Italy1 AJ489744     AACCCTAATCCAGGACCTGATGGCGAGGGTGAAGTTGAATTAGAAAAGGATAGCAATGTTGTTTTAACAACTCAGCGAGATCCTAGTACATCTATTCCAG
France KX373899     AACCCTAATCCAGGACCTGATGGCGAGGGTGAAGTTGAATTAGAAAAGGACAGTAATGTTGTTTTAACAACTCAGCGAGATCCTAGTACATCTATTCCAG
UK1 GU109335        AATCCTAATCCAGGACCTGATGGCGAGGGTGAAGTTGAATTAGAAAAGGATAGTAATGTTGTTTTAACAACTCAGCGAGATCCTAGTACATCTATTCCAG
UK2 KJ437447        AATCCTAATCCTGGTCCAGATGGTGAAGGTGAAGTTGAATTAGAAAAGGATAGTAATGTAGTATTAACTACACAACGTGATCCTAGTACCTCTATTCCTG
Korea1 JX875304     AACCCTAATCCAGGACCTGATGGCGAGGGTGAAGTTGAACTAGAAAAGGATAGTAATGTTGTGTTAACAACTCAGCGAGATCCTAGTACATCTATTCCAG
Korea2 JX878305     AACCCTAATCCAGGACCTGATGGCGAGGGTGAAGTTGAATTAGAAAAGGATAGTAACGTTGTTTTAACAACTCAGCGAGATCCTAGTACATCAATTCCAG
China1 MF770715     AACCCTAATCCAGGACCTGATGGCGAGGGTGAAGTTGAACTAGAAAAGGATAGTAATGTTGTTTTAACAACTCAGCGAGATCCTAGTACATCTATTCCAG
China2 MF036686     AACCCTAATCCAGGACCTGATGGTGAGGGTGAAGTTGAACTAGACAAGGATAGTAATGTTGTTTTAACAACTCAGCGAGATCCTAGTACATCCATTCCAG
China3 MH165180     AATCCTAATCCAGGACCTGATGGCGAGGGTGAAGTTGAATTAGAAAAGGATAGTAATGTTGTTTTAACGACTCAGAGAGATCCTAGTACATCTATTCCAG
DWV-NVN             AATCCTAATCCCGGACCTGATGGAGAAGGTGAAGTTGAATTAGAAAAGGATAGTAATGTTGTTTTAACAACTCAGCGTGATCCTAGTACATCTATTCCAG
DWV-SVN             AATCCTAATCCCGGACCTGATGGAGAAGGTGAAGTTGAATTAGAAAAGGATAGTAATGTTGTTCTAACAACTCAGCGTGATCCTAGTACATCCATTCCAG
```

```
                        1910      1920      1930      1940      1950      1960      1970      1980      1990      2000
                    ....|....|....|....|....|....|....|....|....|....|....|....|....|....|....|....|....|....|....|....|
Japan AB070959      CGCCGGTGAGCGTAAAATGGAGTAGATGGACTAGTAATGATGTAGTAGATGATTACGCCACAATCACATCTCGATGGTATCAGATTGCTGAATTTGTTTG
USA1 AY291384       CGCCGGTGAGCGTAAAATGGAGTAGATGGACTAGTAATGATGTAGTAGATGATTACGCCACAATCACATCTCGATGGTATCAGATTGCTGAATTTGTTTG
Chile JQ413340      CGCCGGTGAGCGTAAAATGGAGTAGATGGACTAGTAATGATGTAGTAGATGATTACGCCACAATTACATCTCGATGGTATCAGATTGCTGAATCTGTTTG
Italy1 AJ489744     CGCCGGTGAGCGTAAAATGGAGTAGATGGACTAGTAATGACGTAGTAGATGATTATGCCACAATCACATCTCGATGGTAYCAGATTGCTGAATTTGTTTG
France KX373899     CGCCGGTGAGCGTAAAATGGAGTAGATGGACTAGTAATGATGTAGTAGATGATTACGCCACAATCACATCTCGATGGTATCAGATTGCTGAATTTGTTTG
UK1 GU109335        CGCCGGTGAGCGTAAAATGGAGTAGATGGACTAGTAATGATGTAGTAGATGATTACGCCACAATCACATCTCGATGGTATCAGATTGCTGAATTTGTTTG
UK2 KJ437447        CTCCAACTAGTGTGAAGTGGAGTAGATGGACTAGTAATGATGTTGTGGATGATTATGCCACTATAACTTCGCGTTGGTATCAGATTGCCGAATTTGTATG
Korea1 JX875304     CGCCGGTGAGCGTAAAATGGAGTAGATGGACTAGTAATGATGTAGTAGATGATTATGCCACAATCACATCTCGATGGTATCAGATTGCTGAATTTGTTTG
Korea2 JX878305     CGCCGGTGAGCGTAAAATGGAGTAGATGGACTAGTAATGATGTAGTAGATGATTACGCCACAATCACATCTCGATGGTATCAGATTGCTGAATTTGTTTG
China1 MF770715     CGCCGGTGAGCGTAAAATGGAGTAGATGGACTAGTAATGATGTAGTAGATGATTATGCTACAATCACATCTCGATGGTATCAGATTGCTGAATTTGTGTG
China2 MF036686     CACCGGTGAGTGTGAAATGGAGTAGATGGACTAGTAATGATGTAGTAGATGATTACGCCACAATCACATCTCGATGGTATCAGATTGCTGAATTTGTTTG
China3 MH165180     CGCCGGTGAGCGTAAAGTGGAGTAGATGGACTAGTAATGATGTGGTAGATGATTACGCCACAATCACATCTCGATGGTATCAGATTGTCGAATTTGTTTG
DWV-NVN             CACCCATGAGCGTAAGATGGAGTAGATGGACTAGTAATGATGTAGTTGATGACTATGCCACAATTACATCTCGGTGGTATCAGATTGCTGAATTCGTTTG
DWV-SVN             CACCTGTGAGCGTAAGATGGAGTAGATGGACTAGTAATGATGTAGTTGATGACTATGCTACAATTACATCTCGGTGGTACCAGATTGCTGAATTCGTTTG
```

```
                      2010      2020      2030      2040      2050      2060      2070      2080      2090      2100
                  ....|....|....|....|....|....|....|....|....|....|....|....|....|....|....|....|....|....|....|....|
Japan AB070959    GTCGAAGGATGATCCATTTGATAAGGAGTTAGCACGTTTAATTTTGCCTCGTGCTTTGTTATCTAGTATAGAGGCTAATTCTGATGCTATATGTGATGTG
USA1 AY291384     GTCGAAGGATGATCCATTTGATAAGGAGTTAGCACGTTTAATTTTGCCTCGTGCTTTGTTATCTAGTATAGAGGCTAATTCTGATGCTATATGTGATGTG
Chile JQ413340    GTCGAAGGATGATCCATTTGATAAGGAGTTGGCACGTTTAATTTTGCCTCGTGCTTCGTTATCTAGTATAGAGGCTAATTCTGATGCTATATGTGATGTG
Italy1 AJ489744   GTCGAAGGATGATCCATTTGATAAGGAGTTAGCACGTTTAATTTTGCCTCGTGCTTTGTTATCTAGTATAGAGGCTAATTCTGATGCTATATGTGATGTG
France KX373899   GTCGAAGGATGATCCATTTGATAAGGAGTTGGCACGTTTAATTTTGCCTCGTGCTTTGTTATCTAGTATAGAGGCTAATTCTGATGCTATATGTGATGTG
UK1 GU109335      GTCGAAGGATGATCCATTTGATAAGGAGTTAGCACGTTTAATTTTGCCTCGTGCTTTGTTATCTAGTATAGAGGCTAATTCTGATGCTATATGTGATGTG
UK2 KJ437447      GTCAAAGGATGATCCATTTGATAAGGAATTGGCGCGTTTAATTTTACCTCGAGCTTTGTTATCTAGTATTGAGGCTAATTCTGACGCTATTTGTGATGTA
Korea1 JX875304   GTCGAAGGATGATCCATTTGATAAGGAGTTGGCACGCTTAATTTTGCCTCGTGCTTTGTTATCTAGTATAGAAGCTAATTCCGACGCTATATGTGATGTG
Korea2 JX878305   GTCGAAGGATGACCCATTTGATAAGGAGTTAGCACGCTTAATTTTGCCTCGTGCTTTGTTATCTAGTATAGAGGCCAATTCTGATGCTATATGTGATGTG
China1 MF770715   GTCGAAGGATGATCCATTTGATAAGGAATTAGCACGCTTAATTTTGCCTCGTGCTTTGTTATCTAGTATAGAGGCTAATTCTGATGCCATATGTGATGTG
China2 MF036686   GTCAAAGGATGATCCATTTGATAAGGAGTTAGCACGTTTAATTTTGCCTCGTGCTTTGTTATCTAGTATAGAGGCTAATTCTGATGCTATATGTGATGTG
China3 MH165180   GTCGAAGGATGATCCATTTGATAAGGAATTGGCACGCTTAATTTTGCCTCGTGCTTTGTTATCTAGTATAGAGGCTAATTCTGATGCTATATGTGATGTG
DWV-NVN           GTCGAAGGATGATCCATTTGATAAAGAATTAGCACGTTTAATTTTGCCTCGTGCTTTATTGTCTAGTATAGAGGCTAATTCTGATGCTATATGTGATGTA
DWV-SVN           GTCGAAGGATGATCCATTTGATAAAGAATTAGCACGTTTAATTTTGCCTCGTGCTTTATTGTCTAGTATAGAGGCTAATTCTGATGCTATATGTGATGTA

                      2110      2120      2130      2140      2150      2160      2170      2180      2190      2200
                  ....|....|....|....|....|....|....|....|....|....|....|....|....|....|....|....|....|....|....|
Japan AB070959    CCTAATACTATTCCATTTAAGGTACCCGCATATTGGCGAGGCGATATGGAAGTTAGAGTTCAAATTAGTTCTAATAAATTCCAAGTTGGTCAATTACAAG
USA1 AY291384     CCTAATACTATCCCATTTAAGGTACACGCATATTGGCGAGGCGATATGGAAGTTAGAGTTCAAATTAATTCAAATAAATTCCAAGTTGGTCAATTACAAG
Chile JQ413340    CCTAATACTATCCCATTTAAGGTACATGCATATTGGCGAGGCGATATGGAAGTTAGAGTTCAAATTAATTCAAATAAATTCCAAGTTGGTCAATTGCAAG
Italy1 AJ489744   CCTAATACTATCCCATTTAAGGTACACGCATATTGGCGAGGCGATATGGAAGTTAGAGTTCAAATTAATTCAAATAAATTCCAAGTTGGTCAATTACAAG
France KX373899   CCTAATACCATCCCATTTAAGGTACACGCATATTGGCGAGGCGATATGGAAGTTAGAGTTCAAATTAATTCAAATAAATTCCAAGTTGGTCAATTACAAG
UK1 GU109335      CCTAATACTATCCCATTTAAGGTACACGCATATTGGCGAGGCGATATGGAAGTTAGAGTTCAAATTAATTCAAATAAATTCCAAGTTGGTCAATTACAAG
UK2 KJ437447      CCTAATACTATTCCGTTTAAGGTACATGCATATTGGCGTGGAGATATGGAAGTTCGAGTGCAGATTAACTCGAATAAATTCCAGGTTGGTCAATTACAGG
Korea1 JX875304   CCTAATACTATCCCATTTAAGGTACACGCATATTGGCGAGGCGATATGGAAGTTAGAGTTCAAATTAATTCAAATAAATTTCAAGTTGGTCAATTACAAG
Korea2 JX878305   CCTAATACTATTCCATTTAAGGTACACGCATATTGGCGAGGCGATATGGAAGTTAGAGTTCAAATTAATTCAAATAAATTCCAAGTTGGTCAATTACAAG
China1 MF770715   CCTAATACTATTCCATTTAAGGTACATGCATACTGGCGAGGCGATATGGAAGTTAGAGTTCAAATTAATTCAAATAAATTCCAAGTTGGTCAATTACAAG
China2 MF036686   CCTAATACCATTCCATTTAAGGTACACGCATATTGGCGAGGCGATATGGAAGTTAGAGTTCAAATTAATTCAAATAAATTCCAAGTTGGTCAATTACAAG
China3 MH165180   CCTAATACTATCCCATTTAAGGTACACGCATATTGGCGAGGCGATATGGAAGTTAGAGTTCAAATTAATTCAAATAAATTCCAAGTTGGTCAATTACAAG
DWV-NVN           CCTAATACCATTCCTTTTAAGGTGCACGCGTATTGGCGCGGCGATATGGAAGTTCGAGTTCAAATTAATTCGAATAAGTTCCAGGTTGGTCAATTGCAAG
DWV-SVN           CCTAATACCATTCCTTTTAAGGTGCACGCGTATTGGCGAGGCGATATGGAAGTTCGAGTTCAAATTAATTCGAATAAATTCCAGGTTGGTCAATTGCAAG

                      2210      2220      2230      2240      2250      2260      2270      2280      2290      2300
                  ....|....|....|....|....|....|....|....|....|....|....|....|....|....|....|....|....|....|....|
Japan AB070959    CTACTTGGTATTATTCGGATCATGAGAATTTGAACATATCGTCTAAGAGAAGCGTTTATGGATTTTCACAAATGGATCATGCTTTGATTAGTGCGTCAGC
USA1 AY291384     CTACTTGGTATTATTCAGATCATGAGGATTTGAATATATCGTCTAAGAGAAGCGTTTATGGATTTTCACAAATGGATCATGCTTTGATTAGTGCGTCAGC
Chile JQ413340    CTACTTGGTATTATTCGGATCATGAGAATTTGAATATATCGTCTAAGAGAAGCGTTTATGGATTTTCACAAATGGATCATGCTTTGATTAGTGCATCAGC
Italy1 AJ489744   CTACTTGGTATTATTCGGATCATGAGAATTTGAATATATCGTCTAAGAGAAGCGTTTATGGATTTTCACAAATGGATCATGCTTTGATTAGTGCGTCAGC
France KX373899   CTACTTGGTATTATTCGGATCATGAGAATTTGAATATATCGTCTAAAAGAAGCGTTTATGGATTTTCACAAATGGATCATGCTTTGATTAGTGCGTCAGC
UK1 GU109335      CTACTTGGTATTATTCGGATCATGAGAATTTGAATATATCGTCTAAGAGAAGCGTTTATGGATTTTCACAAATGGATCATGCTTTGATTAGTGCGTCAGC
UK2 KJ437447      CAACTTGGTACTATTCGGATCATGAAAATTTGAATATCCAGACGAAGCGAAGTGTGTATGGTTTTTCGCATATGGATCATGCTTTGATTAGTGCATCAGC
Korea1 JX875304   CTACTTGGTATTATTCGGATCATGAGAATTTGAATATATCGTCTAAGAGAAGCGTTTATGGATTTTCACAAATGGATCATGCTTTGATTAGTGCGTCAGC
Korea2 JX878305   CTACTTGGTATTATTCGGATCATGAGAATTTGAATATATCGTCTAAGAGAAGTGTTTATGGATTTTCACAAATGGATCATGCTTTGATTAGTGCGTCAGC
China1 MF770715   CTACTTGGTATTATTCGGATCATGAGAATTTGAATATATCGTCTAAGAGAAGCGTTTATGGATTTTCGCAAATGGACCATGCTTTGATTAGTGCGTCAGC
China2 MF036686   CTACTTGGTATTATTCGGATCATGAGAATTTGAATATATCGTCTAAGAGAAGCGTTTATGGTTTTTCACAAATGGATCATGCTTTGATTAGTGCGTCAGC
China3 MH165180   CTACTTGGTACTATTCGGATCATGAGAATTTGAATATATCGTCTAAGAGAAGCGTTTATGGATTTTCACAAATGGATCATGCTTTGATTAGTGCGTCAGC
DWV-NVN           CAACTTGGTATTATTCAGATCATGAAAATTTGAATATAGCATCTAAGAGAAGTGTATATGGATTTTCACAAATGGATCATGCCTTGATTAGTGCATCGGC
DWV-SVN           CAACTTGGTATTATTCAGATCATGAAAATTTGAATATAGCATCTAAAAGAAGTGTATATGGATTTTCACAAATGGATCATGCCTTGATTAGTGCATCGGC

                      2310      2320      2330      2340      2350      2360      2370      2380      2390      2400
                  ....|....|....|....|....|....|....|....|....|....|....|....|....|....|....|....|....|....|....|
Japan AB070959    AAGTAATGAAGCAAAATTAGTTATTCCGTTCAAGCATGTTTATCCATTTTTACCGACAAGAATTGTGCCAGATTGGACTACTGGCATTTTAGATATGGGT
USA1 AY291384     AAGTAATGAAGCAAAATTAGTTATTCCATTTAAGCATGTTTATCCATTTTTACCAACAAGAATTGTGCCAGATTGGACTACTGGCATTTTAGATATGGGT
Chile JQ413340    AAGCAATGAAGCAAAATTAGTTATTCCATTCAAGCACGTTTATCCATTTTTACCGACAAGAATTGTGCCAGATTGGACTACTGGCATTTTAGATATGGGT
Italy1 AJ489744   AAGTAATGAAGCAAAATTAGTTATTCCATATAAGCATGTTTATCCATTTTTACCGACAAGAATTGTGCCAGATTGGACTACTGGCATTTTAGATATGGGT
France KX373899   AAGTAATGAAGCAAAATTAGTTATTCCATATAAGCATGTTTATCCATTCTTACCGACAAGAATTGTGCCAGATTGGACTACTGGCATTCTAGATATGGGT
UK1 GU109335      AAGTAATGAAGCAAAATTAGTTATTCCATTCAAGCATGTTTATCCATTTTTACCGACAAGAATTGTGCCAGATTGGACTACTGGCATTTTAGATATGGGT
UK2 KJ437447      GAGTAATGAAGCAAAATTAGTGATACCTTTTAAACACGTATATCCATTCTTACCAACGCGTGTCGTTCCTGATTGGACAACTGGTATTCTTGATATGGGT
Korea1 JX875304   AAGTAACGAAGCAAAATTAGTTATTCCGTATAAGCATGTTTATCCATTTTTACCGACAAGAATTGTGCCAGATTGGACTACTGGCATTTTAGATATGGGT
Korea2 JX878305   AAGTAAAGAAGCAAAATTAGTTATTCCGTATAAGCATGTTTATCCATTTTTACCGACAAGAATTGTGCCAGATTGGACTACTGGCATTTTAGATATGGGT
China1 MF770715   AAGTAATGAAGCAAAATTAGTTATTCCGTTTAAGCATGTTTATCCATTTTTACCAACAAGAATTGTGCCAGATTGGACTACTGGCATTTTAGATATGGGT
China2 MF036686   AAGTAACGAAGCAAAATTAGTTATTCCATTTAAACATGTTTATCCATTTTTACCGACGAGAATTGTACCAGATTGGACTACTGGCATTTTAGATATGGGT
China3 MH165180   AAGTAACGAAGCAAAATTAGTTATTCCGTTTAAACACGTTTATCCATTTTTACCAACAAGAATTGTGCCAGATTGGACTACTGGCATTTTAGATATGGGT
DWV-NVN           GAGTAATGAAGCAAAATTGGTTATTCCATTTAAACATGTTTATCCATTTTTACCAACACGAATTGTGCCAGACTGGACCACTGGTATTTTGGATATGGGT
DWV-SVN           AAGTAACGAAGCAAAATTAGTTATTCCATTTAAACATGTTTATCCATTTTTACCAACAAGAATTGTGCCAGATTGGACCACTGGTATTTTGGATATGGGT
```

```
                          2410      2420      2430      2440      2450      2460      2470      2480      2490      2500
                    ....|....|....|....|....|....|....|....|....|....|....|....|....|....|....|....|....|....|....|....|
Japan AB070959      GCTTTGAACATTCGTGTAATTGCTCCCTTACGGATGAGTGCTACTGGTCCAACTACCTGTAATGTCGTCGTGTTTATTAAATTAAATAACAGCGAGTTTA
USA1 AY291384       GCTTTGAACATTCGTGTAATTGCTCCCTTACGGATGAGTGCTACTGGTCCAACTACCTGTAATGTCGTTGTGTTTATTAAATTAAATAACAGCGAGTTTA
Chile JQ413340      GCTTTGAACATTCGTGTAATTGCTCCCTTACGGATGAGTGCTACTGGTCCAACTACCTGTAATGTCGTCGTGTTTATTAAATTAAATAACAGCGAGTTTA
Italy1 AJ489744     GCTTTGAACATTCGTGTAATTGCTCCCTTACGGATGAGTGCTACTGGTCCAACTACCTGTAATGTCGTCGTGTTTATTAAATTAAATAACAGCGAGTTTA
France KX373899     GCTTTGAACATTCGTGTAATTGCTCCCTTACGGATGAGTGCTACTGGTCCAACCACTTGTAATGTCGTCGTGTTTATTAAATTAAATAACAGCGAGTTTA
UK1 GU109335        GCTTTGAACATTCGTGTAATTGCTCCCTTACGGATGAGTGCTACTGGTCCAACTACCTGTAATGTCGTTGTGTTTATTAAATTAAATAACAGCGAGTTTA
UK2 KJ437447        ACCTTAAATATTCGTGTGATTGCTCCACTACGTATGAGTGCGACGGGACCAACCACTTGTAATGTTGTAGTATTTATTAAGTTAAATAATAGTGAATTCA
Korea1 JX875304     GCTCTGAACATTCGTGTAATAGCTCCCTTACGGATGAGTGCTACTGGTCCAACTACCTGTAATGTCGTTGTGTTTATTAAATTAAATAACAGCGAGTTTA
Korea2 JX878305     GCTTTGAACATTCGTGTAATAGCTCCCCTACGGATGAGTGCTACTGGCCCAACTACCTGTAAAGTCGTCGTCTTTATTAAGTTAAACAACAGCGAGTTTA
China1 MF770715     GCTTTGAATATTCGTGTTATTGCTCCCTTACGGATGAGTGCTACTGGTCCAACTACTTGTAACGTCGTTGTGTTTATTAAATTAAATAATAGCGAGTTTA
China2 MF036686     GCTTTGAATATTCGTGTAATTGCTCCCTTACGGATGAGTGCTACTGGACCAACTACCTGTAATGTCGTTGTGTTTATTAAATTAAATAACAGCGAGTTTA
China3 MH165180     GCTTTGAATATTCGTGTTATTGCTCCCTTACGGATGAGTGCTACTGGTCCAACTACCTGTAACGTCGTCGTGTTTATTAAATTAAATAATAGCGAGTTTA
DWV-NVN             GCCTTGAATATTCGTGTAATTGCTCCTTTGCGAATGAGTGCTACTGGTCCTACGACTTGTAATGTCGTTGTATTTATTAAATTGAATAATAGTGAGTTTA
DWV-SVN             GCCTTGAATATTCGTGTAATTGCTCCTTTGCGAATGAGTGCTACTGGCCCTACGACTTGTAATGTCGTTGTATTTATTAAACTGAATAATAGTGAGTTTA

                          2510      2520      2530      2540      2550      2560      2570      2580      2590      2600
                    ....|....|....|....|....|....|....|....|....|....|....|....|....|....|....|....|....|....|....|....|
Japan AB070959      CAGGGACTTCTTCTGGTAAGCTTTATGCGAGTCAAATCAGGGCAAAACCTGAGRTGGATCGTATATTAAATTTGGCAGAGGGATTGCTAAATAACACGAT
USA1 AY291384       CAGGGACTTCTTCTGGTAAGTTTTATGCGAGCCAAATCAGGGCAAAACCTGAGATGGATCGTATATTAAATTTAGCAGAGGGATTGTTGAATAACACGAT
Chile JQ413340      CAGGGACTTCCTCTGGTAAGTTTTATGCGAGCCAAATCAGGGCAAAACCTGAGATGGATCGTATATTAAATTTAGCAGAGGGATTGTTGAACAACACGAT
Italy1 AJ489744     CAGGGACTTCTTCTGGTAAGTTTTATGCGAGCCAAATCAGGGCAAAACCTGAGATGGATCGTATATTAAATTTGGCAGAGGGATTGTTGAATAACACGAT
France KX373899     CAGGGACTTCTTCTGGTAAGTTTTATGCGACCCAAATCAGGGCAAAACCTGAGATGGATCGTATATTAAATTTAGCAGAGGGATTGTTGAATAACACGAT
UK1 GU109335        CAGGGACTTCTTCTGGTAAGTTTTATGCGAGTCAAGTCAGGGCAAAACCTGAGATGGATCGTATATTAAATTTGGCAGAGGGATTGTTGAATAATACGAT
UK2 KJ437447        CTGGTACTTCTTCTGGTAAGTTTTACGCGAATCAAATCAGGGCAAAACCTGAAATGGACCGTGTGTTAAATTTGGCAGAAGGATTACTAAATAATACTGT
Korea1 JX875304     CAGGGACCTCTTCTGGTAAGTTTTATGCGAGCCAAATCAGGGTAAAACCTGAGATGGATCGTATATTAAATTTAGCAGAGGGATTGTTGAATAACACGAT
Korea2 JX878305     CAGGGACCTCTTCTGGTAAGTTTTATGCGAGCCAAATCAGGGCAAAACCTGAGATGGATCGTATATTAAATTTAGCAGAGGGGTTGTTGAATAACACGAT
China1 MF770715     CAGGGACTTCTTCTGGTAAGTTTTATGCGAGCCAAATCAGGGCAAAACCTGAGATGGATCGTATATTAAATTTAGCAGAGGGACTATTGAATAACACGAT
China2 MF036686     CAGGAACTTCTTCTGGTAAGTTTTATGCGAGCCAAATCAGGGCAAAACCTGAGATGGATCGTATATTAAATTTAGCAGAGGGATTGCTGAATAATACAAT
China3 MH165180     CAGGGACTTCTTCTGGTAAGTTTTATGCGAGCCAAATCAGGGCAAAACCTGAGATGGATCGTATATTAAATTTAGCAGAGGGACTATTGAATAACACGAT
DWV-NVN             CGGGTACCTCCTCTGGTAAGTTTTATGCGAACCAAATTAGGGCAAAACCTGAGATGGATCGTATATTAAACTTGGCGGAAGGACTATTGAATAATACGAT
DWV-SVN             CGGGTACCTCTTCTGGTAAGTTTTATGCGAACCAAATTAGGGCGAAACCCGAGATGGATCGTATATTAGACTTGGCGGAAGGACTATTGAATAATACGAT

                          2610      2620      2630      2640      2650      2660      2670      2680      2690      2700
                    ....|....|....|....|....|....|....|....|....|....|....|....|....|....|....|....|....|....|....|....|
Japan AB070959      TGGTGGTAACAATATGGATAATCCTTCTTATCAACAATCTCCTCGTCATTTTGTCCCGACTGGTATGCACAGTTTAGCTTTAGGTACTAATTTAGTTGAA
USA1 AY291384       TGGTGGTAATAATATGGATAATCCTTCTTATCAACAATCTCCTCGTCATTTTGTCCCGACTGGTATGCACAGCTTAGCTTTAGGTACTAATTTAGTTGAA
Chile JQ413340      TGGTGGTAATAATATGGATAATCCTTCTTATCAACAATCTCCTCGTCATTTTGTCCCGACTGGTATGCACAGCTTAGCTTTAGGTACTAATTTAGTTGAA
Italy1 AJ489744     TGGTGGTAATAATATGGATAATCCTTCTTATCAACAATCTCCTCGTCATTTTGTCCCGACTGGTATGCACAGCTTAGCTTTAGGTACTAATTTAGTTGAA
France KX373899     TGGTGGTAATAATATGGATAATCCTTCTTATCAACAATCTCCTCGTCATTTTGTCCCGACTGGTATGCACAGCTTAGCTTTAGGTACTAATTTAGTTGAA
UK1 GU109335        TGGTGGTAATAATATGGATAATCCTTCTTATCAACAATCTCCTCGTCATTTTGTCCCGACTGGTATGCACAGCTTAGCTTTAGGTACTAATTTAGTTGAA
UK2 KJ437447        AGGTGGTTGTAATATGGATAACCCGTCATATCAGCAATCTCCGCGTCATTTTGTCCCTACTGGTATGCATAGTTTAGCTTTAGGCACTAATCTAGTAGAG
Korea1 JX875304     TGGTGGTAATAATATGGATAATCCTTCTTATCAACAATCTCCTCGTCATTTTGTTCCGACTGGTATGCACAGTTTAGCTTTAGGTACTAATTTAGTTGAA
Korea2 JX878305     TGGTGGTAATAATATGGATAATCCTTCTTATCAACAATCTCCTCGTCATTTTGTGCCGACTGGTATGCACAGCTTAGCTTTAGGTACTAATTTAGTTGAA
China1 MF770715     TGGTGGTAATAATATGGATAATCCCTCTTATCAACAATCTCCTCGCCATTTTGTCCCGACTGGTATGCACAGTTTAGCTTTAGGTACTAATTTAGTTGAA
China2 MF036686     TGGTGGTAATAATATGGATAATCCTTCTTATCAACAATCTCCTCGTCATTTTGTCCCGACTGGCATGCACAGTTTAGCTTTAGGTACTAATTTAGTTGAA
China3 MH165180     TGGTGGTAATAATATGGATAATCCTTCTTATCAACAATCTCCTCGCCATTTTGTCCCGACTGGTATGCACAGTTTAGCTTTAGGTACTAATTTAGTTGAA
DWV-NVN             CGGTGGTAATAACATGGATAATCCTTCATATCAACAGTCCCCTCGCCATTTTGTCCCGACTGGTATGCACAGTTTAGCTTTAGGTACTAATTTAGTTGAA
DWV-SVN             CGGTGGTAATAACATGGATAATCCTTCATATCAACAGTCTCCTCGCCATTTTGTCCCGACTGGTATGCATAGTTTAGCTTTAGGTACTAATTTAGTTGAA

                          2710      2720      2730      2740      2750      2760      2770      2780      2790      2800
                    ....|....|....|....|....|....|....|....|....|....|....|....|....|....|....|....|....|....|....|....|
Japan AB070959      CCATTACATGCATTACGTTTGGATGCGGCTGGTACGACACAACATCCTGTAGGTTGTGCTCCTGATGAAGATATGACTGTATCATCCATAGCATCTCGAT
USA1 AY291384       CCATTACATGCATTACGTTTGGATGCAGCCGGTACGACACAACATCCTGTAGGTTGTGCTCCTGATGAAGATATGACTGTATCTTCCATTGCATCTCGAT
Chile JQ413340      CCATTACATGCATTACGTTTGGATGCAGCTGGTACGACACAACATCCTGTAGGTTGTGCTCCTGATGAAGATATGACGGTATCCTCCATTGCATCTCGAT
Italy1 AJ489744     CCATTACATGCATTACGTTTRGATGCAGCCGGTACGACACAACATCCTGTAGGTTGTGCTCCTGATGAAGATATGACTGTATCCTCCATTGCATCTCGAT
France KX373899     CCATTACATGCATTACGTTTGGATGCAGCCGGTACGACACAACATCCTGTAGGTTGTGCTCCTGATGAAGACATGACTGTATCCTCCATTGCATCTCGAT
UK1 GU109335        CCATTGCATGCATTACGTTTGGATGCAGCTGGTACGACACAACACCCTGTAGGTTGTGCTCCTGATGAAGATATGACTGTCTCCTCCATTGCGTCTCGAT
UK2 KJ437447        CCTTTGCATGCATTACGATTAGATGCATCAGGTACAACACAACATCCAGTTGGGTGTGCGCCTGATGAAGATATGACTGTATCTTCCATTGCATCACGAT
Korea1 JX875304     CCATTACATGCATTACGTCTGGATGCAGCCGGTACGACACAACATCCTGTAGGTTGTGCTCCTGATGAAGATATGACTGTATCTTCCATTGCATCTCGAT
Korea2 JX878305     CCATTACATGCATTACGTTTGGATGCAGCCGGTACGACACAACATCCTGTAGGTTGTGCTCCTGATGAAGATATGACCGTATCATCCATTGCATCTCGAT
China1 MF770715     CCATTACATGCATTACGTTTAGATGCGGCCGGTACGACACAACATCCTGTAGGTTGTGCTCCTGATGAAGATATGACTGTATCTTCCATTGCATCTCGAT
China2 MF036686     CCATTACATGCATTACGTTTGGATGCGGCTGGTACGACACAACATCCTGTAGGTTGTGCTCCTGATGAGGATATGACTGTATCCTCCATTGCATCTCGAT
China3 MH165180     CCATTACATGCATTACGTTTAGATGCGGCTGGTACGACACAACATCCTGTAGGTTGTGCTCCTGATGAAGATATGACTGTATCCTCCATTGCATCTCGAT
DWV-NVN             CCATTACATGCATTACGTTTAGACGCAGCCGGTACGACACAACATCCTGTTGGCTGTGCTCCTGATGAAGATATGACTGTATCCTCCATTGCATCTCGAT
DWV-SVN             CCATTACATGCATTACGTTTAGACGCAGCCGGTACGACACAACATCCTGTTGGTTGTGCTCCTGATGAAGATATGACTGTATCCTCCATTGCATCTCGAT
```

```
                           2810      2820      2830      2840      2850      2860      2870      2880      2890      2900
                   ....|....|....|....|....|....|....|....|....|....|....|....|....|....|....|....|....|....|....|....|
Japan AB070959     ATGGACTAATTAGACAGATACAATGGAAGAAAGATCATGCTAAAGGATCACTTTTGTTACAGTTAGATGCTGATCCATTTGTGGAGCAAAGAATTGAAGG
USA1 AY291384      ATGGACTAATTAGACGGGTACAATGGAAGAAAGATCATGCTAAAGGATCACTTTTGTTACAATTAGATGCTGATCCATTTGTGGAGCAAAGAATTGAAGG
Chile JQ413340     ATGGACTAATTAGACGGGTACAATGGAAGAAAGATCATGCTAAAGGATCACTTTTGTTACAATTAGATGCTGATCCATTTGTGGAGCAAAGAATTGAAGG
Italy1 AJ489744    ATGGACTAATTAGACGGGTACAATGGAAGAAAGATCATGCTAAAGGATCACTTTTGTTACAATTAGATGCTGATCCATTTGTGGAGCAAAGAATTGAAGG
France KX373899    ATGGACTAATTAGACGGGTACAATGGAAGAAAGATCATGCTAAAGGATCGCTTTTGTTACAATTAGATGCTGATCCATTTGTGGAGCAAAGAATTGAAGG
UK1 GU109335       ATGGACTAATTAGACGGGTCCAATGGAAGAAAGATCATGCTAAAGGATCACTTTTGTTACAATTAGATGCTGATCCATTTGTGGAGCAAAGAATTGAAGG
UK2 KJ437447       ACGGTTTAATTCGCCAAGTGCAATGGAAGAAAGACCATGCGAAAGGATCATTATTATTACAACTTGACGCTGATCCTTTCGTTGAACAGAAAATTGAGGG
Korea1 JX875304    ATGGATTAATTAGACAGGTACAATGGAAGAAAGATCATGCTAAAGGAGCACTTTTGTTACAATTGGATGCTGATCCGTTTGTGGAGCAAAGAATTGAAGG
Korea2 JX878305    ATGGACTAATTAGACAGGTACAATGGAAGAAAGATCATGCGAAAGGATCACTTTTGTTACAATTAGATGCTGATCCATTTGTGGAGCAAAGAATTGAAGG
China1 MF770715    ATGGATTAATTAGACAGGTACAATGGAAGAAAGATCATGCTAAAGGATCACTTTTGTTACAATTAGATGCTGATCCATTTGTGCAGCAAAGAATTGAAGG
China2 MF036686    ATGGATTAATTAGACAGGTACAATGGAAGAAAGATCATGCTAAAGGATCACTTTTGTTACAGTTAGATGCTGATCCGTTTGTGCAGCGAAGAATTGAAGG
China3 MH165180    ATGGACTAATTAGACAGGTGCAATGGAAGAAAGATCATGCTAAAGGATCACTTTTGTTACAATTAGATGCTGATCCATTTGTGCAGCAAAGAATTGAAGG
DWV-NVN            ATGGATTAATTAGACAGGTACAATGGAAGAAAGACCATGCTAAAGGATCACTTTTGTTACAACTTGATGCCGATCCGTTCGTGGAGCAAAAGATTGAAGG
DWV-SVN            ATGGATTAATTAGACAGGTACAATGGAAAAAAGACCATGCTAAAGGATCACTTTTGTTACAACTTGACGCCGATCCGTTCGTGGAGCAAAAGATTGAAGG

                           2910      2920      2930      2940      2950      2960      2970      2980      2990      3000
                   ....|....|....|....|....|....|....|....|....|....|....|....|....|....|....|....|....|....|....|....|
Japan AB070959     TACGAATCCGATATCTCTGTATTGGTTCGCACCCGTGGGTGTAGTATCTAGTATGTTTATGCAATGGCGTGGTTCATTAGAATATAGGTTTGATATTATA
USA1 AY291384      TACGAATCCAATATCTTTGTATTGGTTCGCGCCCGTGGGTGTAGTATCTAGTATGTTTATGCAATGGCGTGGTTCATTAGAATATAGGTTTGATATTATA
Chile JQ413340     TACGAATCCAATATCTTTGTATTGGTTCGCACCTGTGGGTGTAGTATCTAGTATGTTTATGCAATGGCGCGGTTCATTAGAATATAGGTTTGATATTATA
Italy1 AJ489744    TACGAATCCAATATCYTTGTATTGGTTCGCACCCGTGGGTGTAGTATCTAGTATGTTTATGCAATGGCGCGGTTCATTAGAATATAGGTTTGATATTATA
France KX373899    TACGGGTCCAATATCTTTGTATTGGTTTGCACCCGTGGGTGTCGTATCTAGTATGTTTATGCAATGGCGTGGTTCATTAGAATATAGGTTTGATATTATA
UK1 GU109335       TACGAATCCAATATCTTTGTATTGGTTCGCACCCGTGGGTGTAGTATCTAGTATGTTTATGCAATGGCGCGGTTCATTAGAATATAGGTTTGATATTATA
UK2 KJ437447       AACCAATCCAATTTCTTTGTATTGGTTTGCTCCGGTTGGAGTCGTATCTAGTATGTTTATGCAATGGAGAGGTTCTTTAGAATATAGATTTGACATTATA
Korea1 JX875304    TACGAATCCAATATCTTTGTATTGGTTCGCACCCGTGGGTGTAGTATCTAGTATGTTTATGCAATGGCGTGGTTCATTAGAATATAGGTTTGATATTATA
Korea2 JX878305    TACGAATCCAATATCTCTGTATTGGTTCGCACCCGTGGGTGTAGTATCTAGTATGTTTATGCAATGGCGTGGTTCATTAGAATATAGGTTTGATATTATT
China1 MF770715    TACGAATCCAATATCCTTGTATTGGTTCGCACCCGTGGGTGTAGTATCTAGTATGTTTATGCAATGGCGTGGTTCATTAGAATATAGGTTTGATATTATA
China2 MF036686    TACGAATCCAATATCTCTGTATTGGTTCGCACCCGTGGGTGTTGTATCTAGTATGTTTATGCAATGGCGTGGTTCACTAGAATATAGGTTTGATATTATA
China3 MH165180    TACGAATCCAATATCTTTGTATTGGTTCGCACCCGTGGGTGTAGTATCTAGTATGTTTATGCAATGGCGTGGTTCGTTAGAATATAGGTTTGATACTATA
DWV-NVN            TACGAATCCAATATCTTTGTATTGGTTCGCGCCCGTAGGCGTTGTGTCTAGCATGTTTATGCAATGGCGTGGTTCATTAGAATATAGGTTTGATATTATA
DWV-SVN            TACGAATCCAATATCTCTGTACTGGTTCGCGCCCGTAGGCGTTGTGTCTAGCATGTTTATGCAATGGCGTGGTTCATTAGAATATAGGTTTGATATTATA

                           3010      3020      3030      3040      3050      3060      3070      3080      3090      3100
                   ....|....|....|....|....|....|....|....|....|....|....|....|....|....|....|....|....|....|....|....|
Japan AB070959     GCATCCCAATTTCATACTGGTAGGTTAATTGTAGGTTATGTGCCTGGTTTGACAGCATCTTTACAACAGCAAATGGACTATATGAAGTTGAAGTCATCAA
USA1 AY291384      GCATCCCAATTTCATACTGGTAGGTTAATTGTAGGTTATGTGCCCGGTTTGACAGCATCTTTGCAACTTCAAATGGACTATATGAAATTGAAGTCATCGA
Chile JQ413340     GCATCCCAATTTCATACTGGTAGGTTAATTGTAGGTTATGTGCCCGGTTTGACAGCATCTTTGCAACTTCAAATGGACTATATGAAATTGAAGTCATCGA
Italy1 AJ489744    GCATCCCAATTTCATACTGGTAGGTTAATTGTAGGTTATGTGCCCGGTTTGACAGCATCTTTGCAACTTCAAATGGACTATATGAAATTGAAGTCATCGA
France KX373899    GCATCTCAATTTCATACTGGTAGGTTAATTGTAGGTTATGTGCCCGGTTTAACAGCATCTTTGCAACTTCAAATGGACTATATGAAATTGAAGTCATCGA
UK1 GU109335       GCATCCCAATTTCATACTGGTAGGTTAATTGTAGGTTATGTGCCCGGTTTGACAGCATCTTTGCAACTTCAAATGGACTATATGAAATTGAAGTCATCGA
UK2 KJ437447       GCTTCCCAATTTCATACGGGTAGGTTAATTGTAGGTTATGTTCCTGGACTGACTGCTTCTTTACAACGTCAAATGGACTATATGAAATTGAAATCATCTA
Korea1 JX875304    GCATCCCAATTTCATACTGGTAGGTTAATTGTAGGTTATGTGCCTGGTTTGACAGCATCTTTGCAACTTCAAATGGACTATATGAAGTTGAAGTCATCAA
Korea2 JX878305    GCATCCCAGTTTCATACTGGTAGGTTAATTGTAGGTTATGTGCCTGGTTTGACAGCATCTTTGCAACAGCAAATGGACTATATGAAGTTGAAGTCATCAA
China1 MF770715    GCGTCCCAATTTCATACTGGTAGGTTAATTGTAGGTTATGTGCCCGGTTTGACAGCATCTTTGCGACAACAAATGGACTATATGAAGTTGAAGTCATCGA
China2 MF036686    GCATCCCAATTTCATACTGGTAGGTTAATTGTAGGTTATGTGCCTGGTTTGACAGCATCTTTGCAACAGCAAATGGACTATATGAAGTTGAAGTCATCAA
China3 MH165180    GCATCCCAATTTCATGCTGGTAGGTTAATTGTAGGTTATGTGCCTGGTTTGACAGCATCTTTGCAACAGCAAATGGACTATATGAAGTTGAAGTCATCAA
DWV-NVN            GCATCCCAATTTCATACTGGTAGGCTGATTGTAGGTTATGTGCCTGGCTTGACGGCATCGTTGCAGCGCCAAATGGACTATATGAAATTGAAATCATCTA
DWV-SVN            GCATCCCAATTTCATACTGGTAGGTTGATTGTAGGTTATGTGCCTGGCTTGACGGCGTCGTTGCAGCGCCAAATGGACTATATGAAATTGAAATCATCTA

                           3110      3120      3130      3140      3150      3160      3170      3180      3190      3200
                   ....|....|....|....|....|....|....|....|....|....|....|....|....|....|....|....|....|....|....|....|
Japan AB070959     GTTATGTAGTATTTGATTTACAAGAAAGTAATAGCTTTACTTTTGAGGTGCCATATGTTTCATACAGACCATGGTGGGTGCGTAAGTATGGTGGCAATTA
USA1 AY291384      GTTATGTAGTATTTGATTTACAGGAAAGTAATAGCTTCACTTTTGAGGTGCCATATGTTTCATATAGACCATGGTGGGTGCGTAAATATGGTGGCAATTA
Chile JQ413340     GTTATGTAGTATTTGATTTACAAGAAAGTAATAGCTTCACTTTTGAGGTGCCATAYGTTTCATATAGACCATGGTGGGTGCGTAAATATGGTGGYAATTA
Italy1 AJ489744    GTTATGTAGTATTTGATTTACAAGAAAGTAATAGCTTCACTTTTGAGGTGCCATAYGTTTCATATAGACCATGGTGGGTGCGTAAATATGGTGGYAATTA
France KX373899    GCTATGTAGTTTTTGATTTACAAGAAAGTAATAGCTTCACCTTTGAGGTGCCATATGTTTCATATAGACCATGGTGGGTGCGTAAATATGGTGGTAATTA
UK1 GU109335       GTTATGTAGTATTTGATTTACAAGAGAGTAATAGCTTCACTTTTGAGGTGCCATATGTTTCATATAGACCATGGTGGGTGCGCAAATATGGTGGCAATTA
UK2 KJ437447       GTTATGTGGTGTTTGATTTACAGGAAAGTAATAGTTTTACGTTTGAAGTGCCCTATGTGTCATATAGACCGTGGTGGGTGCGTAAGTATGGTGGTAATTA
Korea1 JX875304    GTTATGTAGTATTTGATTTACAAGAAAGTAATAGCTTTACTTTTGAGGTACCATATGTTTCATACAGACCATGGTGGGTGCGTAAGTATGGTGGTAATTA
Korea2 JX878305    GTTATGTAGTATTCGATTTACAAGAAAGTAATAGCTTTACTTTTGAGGTACCATATGTTTCATACAGACCATGGTGGGTGCGTAAGTATGGTGGCAATTA
China1 MF770715    GTTATGTAGTATTTGATTTACAAGAAAGTAATAGCTTTACTTTTGAGGTGCCGTATGTTTCATACAGACCATGGTGGGTGCGTAAGTATGGTGGCAATTA
China2 MF036686    GTTATGTAGTGTTTGATTTACAAGAAAGTAATAGTTTTACTTTTGAGGTACCATATGTTTCATATAGACCATGGTGGGTGCGTAAGTATGGTGGCAATTA
China3 MH165180    GTTACGTGGTATTTGATTTACAAGGAAGTAATAGTTTTACTTTTGAGGTACCATATGTTTCATACAGGCCATGGTGGGTGCGTAAGTATGGTGGCAATTA
DWV-NVN            GTTATGTGGTATTTGACTTACAAGAAAGTAATAGTTTTACTTTTGAAGTACCATACGTTTCATATAGACCATGGTGGGTGCGTAAGTATGGTGGTAATTA
DWV-SVN            GTTATGTGGTATTTGATTTACAAGAAAGTAATAGTTTTACTTTTGAAGTACCATATGTTTCATATAGACCATGGTGGGTGCGTAAGTATGGTGGTAATTA
```

```
                            3210      3220      3230      3240      3250      3260      3270      3280      3290      3300
                    ....|....|....|....|....|....|....|....|....|....|....|....|....|....|....|....|....|....|....|....|
Japan AB070959      TTTACCCTCGTCAACTGACGCTCCTAGCACATTATTTATGTATGTGCAGGTTCCGTTGATACCTATGGAAGCTGTTTCAGATACTATTGATATCAATGTG
USA1 AY291384       TTTACCCTCGTCAACTGACGCTCCTAGTACATTATTTATGTATGTGCAGGTTCCGTTGATACCTATGGAAGCTGTTTCAGATACTATTGATATCAATGTG
Chile JQ413340      TTTACCCTCGTCAACTGACGCTCCTAGTACATTATTTATGTATGTKCAGGTWCCRTTGATACCTATGGAAGCTGTTTCAGATACTATTGATATCAATGTG
Italy1 AJ489744     TTTACCCTCGTCAACTGACGCTCCTAGTACATTATTTATGTATGTKCAGGTWCCRTTGATACCTATGGAAGCTGTTTCAGATACTATTGATATCAATGTG
France KX373899     TCTACCCTCGTCAACTGACGCTCCTAGTACATTATTTATGTATGTGCAGGTTCCGTTGATTCCTATGGAAGCTGTTTCAGATACTATTGATATCAATGTG
UK1 GU109335        TTTGCCCTCGTCAACTGACGCTCCTAGTACATTATTTATGTATGTGCAGGTTCCGTTGATACCTATGGAAGCTGTTTCAGATACTATTGATATCAATGTG
UK2 KJ437447        TCTGCCATCTTCTACTGATGCGCCTAGCACACTGTTTATGTATGTACAAGTACCATTGATACCTATGGAAGCTGTTTCTGATACTATAGATATCAATGTG
Korea1 JX875304     TTTACCCTCGTCAACTGACGCTCCTAGTACATTATTTATGTATGTGCAGGTTCCGTTGATACCTATGGAAGCTGTTTCAGATACTATTGATATCAATGTG
Korea2 JX878305     TTTACCCTCGTCAACTGACGCTCCTAGCACATTATTTATGTATGTGCAGGTACCGTTGATACCTATGGAAGCTGTTTCAGATACTATTGATATCAATGTG
China1 MF770715     TTTACCCTCGTCAACTGATGCTCCTAGTACATTATTTATGTATGTGCAGGTTCCGTTGATACCTATGGAAGCTGTTTCAGATACTATTGATATCAATGTG
China2 MF036686     TTTACCTTCGTCAACTGACGCTCCTAGTACATTATTCATGTATGTGCAGGTTCCGTTGATACCTATGGAAGCTGTTTCAGATACTATTGATATCAATGTG
China3 MH165180     TTTACCCTCGTCAACTGACGCTCCTAGTACATTATTTATGTATGTACAAGTTCCGTTGATACCTATGGAAGCTGTTTCAGATACTATTGATATCAATGTG
DWV-NVN             TTTGCCCTCGTCAACTGATGCTCCTAGCACATTATTTATGTATGTTCAAGTTCCGTTGATACCTATGGAAGCTGTTTCAGATACCATAGACATTAATGTG
DWV-SVN             TTTGCCCTCGTCAACTGATGCTCCTAGCACATTATTTATGTATGTTCAAGTTCCGTTGATACCTATGGAAGCTGTTTCAGACACCATAGATATTAATGTG

                            3310      3320      3330      3340      3350      3360      3370      3380      3390      3400
                    ....|....|....|....|....|....|....|....|....|....|....|....|....|....|....|....|....|....|....|....|
Japan AB070959      TACGTACGGGGCGGTAGTTCATTTGAAGTTTGTGTTCCAGTCCAACCTAGTTTAGGTTTGAATTGGAATACAGACTTTATTTTACGTAATGACGAAGAAT
USA1 AY291384       TACGTACGGGGCGGTAGTTCATTTGAAGTTTGTGTTCCAGTCCAACCTAGTTTAGGTTTGAATTGGAACACAGACTTTATTTTACGTAATGACGAAGAAT
Chile JQ413340      TACGTACGGGGCGGTAGTTCATTTGAAGTTTGTGTTCCAGTCCAACCTAGTTTAGGTTTGAATTGGAATACAGACTTTATTTTACGTAATGACGAAGAAT
Italy1 AJ489744     TACGTACGGGGCGGTAGTTCATTTGAAGTTTGTGTTCCAGTCCAACCTAGTTTAGGTTTGAATTGGAATACAGACTTTATTTTACGTAATGAYGAAGAAT
France KX373899     TACGTACGGGGCGGTAGTTCATTTGAAGTTTGTGTTCCAGTTCAACCTAGTTTAGGTTTGAACTGGAATACAGACTTTATTTTACGTAATGACGAAGAAT
UK1 GU109335        TACGTACGGGGCGGTAGTTCATTTGAAGTTTGTGTGCCAGTCCAACCTAGTTTAGGTTTGAATTGGAATACAGACTTTATTTTACGTAATGACGAAGAAT
UK2 KJ437447        TATGTGCGTGGTGGCAGTTCGTTTGAGGTTTGTGTTCCAGTCCAACCTAGTTTAGGTTTGAACTGGAATACAGATTTCATATTACGTAATGATGAGGAGT
Korea1 JX875304     TACGTACGGGGCGGTAGTTCGTTTGAAGTTTGTGTTCCAGTCCAACCTAGTTTAGGTTTGAATTGGAATACTGACTTTATTTTACGTAATGACGAAGAAT
Korea2 JX878305     TACGTACGGGGCGGTAGTTCGTTTGAAGTTTGTGTTCCAGTCCAACCTAGTTTAGGTTTGAATTGGAATACAGACTTTATTTTGCGTAATGACGAAGAAT
China1 MF770715     TACGTGCGGGGCGGTAGTTCATTTGAAGTTTGTGTTCCGGTCCAACCTAGTTTAGGTTTGAATTGGAATACAGACTTTATTTTACGTAACGACGAAGAAT
China2 MF036686     TATGTACGGGGCGGTAGTTCGTTTGAAGTTTGTGTTCCAGTCCAACCTAGTTTAGGTTTGAATTGGAATACAGACTTTATTTTACGTAATGATGAAGAAT
China3 MH165180     TCCGTACGGGGCGGTAGTTCGTTTGAAGTTTGTGTTCCAGCCCAACCTAGTTTAGGTTTGAATTGGAATACAGACTTTATTTTACGTAATGACGAGGAAT
DWV-NVN             TATGTGCGAGGAGGTAGTTCGTTTGAAGTTTGTGTCCCAGTGCAACCTAGTTTGGGTCTGAATTGGAACACGGACTTCATATTACGTAATGACGAAGAAT
DWV-SVN             TATGTGCGAGGAGGTAGTTCATTTGAAGTTTGTGTCCCAGTGCAACCTAGCTTGGGTTTGAATTGGAACACGGACTTCATATTGCGTAATGACGAAGAAT

                            3410      3420      3430      3440      3450      3460      3470      3480      3490      3500
                    ....|....|....|....|....|....|....|....|....|....|....|....|....|....|....|....|....|....|....|....|
Japan AB070959      ATAGGGCTAAGACAGGTTATGCACCATATTATGCTGGAGTATGGCATAGCTTCAATAATAGTAACTCTCTTGTTTTTAGGTGGGGATCTGCTTCTGATCA
USA1 AY291384       ACAGGGCTAAGACAGGTTATGCACCATATTATGCTGGAGTGTGGCATAGCTTCAATAATAGTAATTCTCTTGTTTTTAGGTGGGGATCTGCTTCTGATCA
Chile JQ413340      ATAGGGCTAAGACAGGTTATGCACCATATTATGCTGGAGTGTGGCATAGCTTCAATAATAGTAATTCTCTTGTTTTTAGGTGGGGATCTGCTTCTGATCA
Italy1 AJ489744     ACAGGGCTAAGACAGGTTATGCACCATATTATGCTGGAGTGTGGCATAGCTTCAATAATAGTAATTCTCTTGTTTTTAGGTGGGGATCTGYTTCCGAYCA
France KX373899     ACAGGGCTAAGACAGGTTATGCACCATATTATGCTGGAGTGTGGCATAGCTTCAATAATAGTAATTCTCTTGTTTTTAGGTGGGGATCTGCTTCTGATCA
UK1 GU109335        ACAGGGCTAAGACAGGTTATGCACCATATTATGCTGGAGTGTGGCATAGCTTCAATAATAGTAATTCTCTTGTTTTTAGGTGGGGATCTGCTTCCGATCA
UK2 KJ437447        ACCGCGCAAAGAATGGATATGCACCATATTATGCTGGTGTGTGGCATAGCTTCAATAATAGTAATTCGCTTGTTTTTAGATGGGGTTCGGCTTCAGATCA
Korea1 JX875304     ATAGGGCTAAGACGGGTTATGCACCATATTATGCAGGAGTGTGGCATAGCTTCAATAATAGTAATTCTCTTGTTTTTAGGTGGGGATCTGCTTCTGATCA
Korea2 JX878305     ATAGGGCTAAGACAGGTTATGCACCATATTATGCAGGAGTGTGGCATAGCTTCAATAATAGTAATTCTCTCGTTTTTAGGTGGGGATCTGCTTCTGATCA
China1 MF770715     ATAGGGCTAAGACAGGTTATGCACCATATTATGCTGGAGTGTGGCATAGTTTCAATAATAGTAATTCTCTTGTTTTTAGGTGGGGATCGGCTTCTGATCA
China2 MF036686     ATAGGGCTAAGACGGGCTATGCACCATATTATGCTGGAGTATGGCATAGCTTCAATAATAGTAATTCTCTTGTATTTAGGTGGGGATCTGCTTCTGATCA
China3 MH165180     ATAGGGCTAAGACGGGTTATGCGCCATATTATGCCGGAGTGTGGCATAGCTTCAATAATAGTAATTCTCTTGTTTTTAGGTGGGGATCTGCTTCTGATCA
DWV-NVN             ATCGGGCTAAGACTGGTTACGCACCATATTATGCTGGAGTGTGGCATAGCTTTAATAATAGTAATTCTCTTATTTTCAGATGGGGATCTGCTTCTGATCA
DWV-SVN             ATCGGGCTAAGACTGGTTACGCACCATATTATGCTGGAGTGTGGCATAGCTTCAATAACAGTAATTCTCTTGTTTTCAGATGGGGATCTGCTTCTGATCA

                            3510      3520      3530      3540      3550      3560      3570      3580      3590      3600
                    ....|....|....|....|....|....|....|....|....|....|....|....|....|....|....|....|....|....|....|....|
Japan AB070959      AATTGCTCAGTGGCCAACAATTTCAGTACCAAGAGGTGAGCTAGCTTTCTTACGMATTARGGATGGAAAGCRAGCTGCTGTAGGAACTCAACCTTGGCGT
USA1 AY291384       AATTGCTCAGTGGCCGACAATTTCAGTACCAAGAGGTGAGCTAGCTTTCTTACGAATTAAGGATGGAAAGCAAGCTGCTGTAGGAACTCAACCTTGGCGT
Chile JQ413340      AATTGCTCAGTGGCCGACAATTTCAGTACCAAGAGGTGAGTTAGCTTTCTTACGAATTAAGGATGGAAAGCAAGCTGCTGTAGGAACTCAACCTTGGCGT
Italy1 AJ489744     AATTGCTCAGTGGCCGACAATTTCAGTACCAAGAGGTGAGCTAGCTTTCTTACGAATTAAGGATGGAAAGCAAGCTGCTGTAGGAACYCAACCTTGGCGT
France KX373899     AATTGCTCAGTGGCCGACAATTTCAGTACCAAGAGGTGAGCTAGCTTTCTTACGAATTAGGGATGGAAAGCAAGCTGCTGTAGGAACTCAACCTTGGCGT
UK1 GU109335        AATTGCTCAGTGGCCGACAATTTCAGTACCAAGAGGTGAGCTAGCTTTCTTACGAATTAAGGATGGAAAGCAAGCTGCTGTAGGAACTCAACCTTGGCGT
UK2 KJ437447        AATTGCTCAATGGCCAACAATAACAGTGCCTCGAGGAGAGTTGGCATTCTTGCGTATCCGCGATGCTAAGCAAGCTGCTGTAGGAACGCAACCTTGGCGT
Korea1 JX875304     AATTGCTCAGTGGCCGACAATTTCAGTACCAAGAGGTGAGCTAGCTTTCTTACGAATTAGGGATGGAAAGCAAGCTGCTGTAGGAACTCAGCCTTGGCGT
Korea2 JX878305     AATTGCTCAGTGGCCGACAATTTCAGTGCCAAGAGGTGAGCTAGCTTTCTTACGAATTAGGGATGGAAAGCAAGCTGCTGTAGGAACTCAACCTTGGCGT
China1 MF770715     AATTGCTCAGTGGCCAACAATTTCAGTACCAAGAGGTGAGCTAGCTTTCTTACGAATTAGGGATGGAAAGCAAGCTGCTGTAGGAACTCAACCTTGGCGT
China2 MF036686     AATTGCTCAGTGGCCAACGATTTCAGTACCAAGAGGTGAGCTAGCTTTCTTACGAATTAGGGATGGAAAGCAAGCTGCTGTAGGAACTCAACCTTGGCGT
China3 MH165180     AATTGCTCAGTGGCTGACAATTGCAGTACCAAGAGGTGAGCTAGCTTTCTTACGAATTGGGGATGGAAAGCAAGCTGCTGTAGGAACTCAACCTTGGCGT
DWV-NVN             AATCGCTCAGTGGCCTACTATATCAGTGCCTAGGGGTGAACTTGCTTTCTTACGAATTAAAGACGGAAAGAAAGCTGCTGTTGGAACTCAACCTTGGCGT
DWV-SVN             AATTGCTCAGTGGCCTACTATATCAGTGCCTAGGGGTGAACTTGCTTTCTTACGAATTAAAGACGGAAAGAAAGCTGCTGTTGGAACTCAACCTTGGCGT
```

```
                   3610      3620      3630      3640      3650      3660      3670      3680      3690      3700
               ....|....|....|....|....|....|....|....|....|....|....|....|....|....|....|....|....|....|....|....|
Japan AB070959 ACGATGGTTGTTTGGCCTTCTGGTCATGGTTATAATATTGGTATACCTACGTATAATGCTGAACGAGCTCGCCAGCTTGCACAGCATTTATATGGTGGTG
USA1 AY291384   ACGATGGTTGTTTGGCCTTCTGGTCATGGCTATAATATTGGTATACCTACGTATAATGCTGAACGAGCTCGCCAGCTTGCACAACACTTATATGGTGGTG
Chile JQ413340 ACGATGGTTGTTTGGCCTTCTGGTCATGGTTATAATATTGGTATACCTACGTATAATGCTGAACGAGCTCGCCAGCTTGCACAACACTTATATGGTGGTG
Italy1 AJ489744 ACGATGGTTGTTTGGCCTTCTGGYCATGGTTATAATATTGGTATACCTACGTATAATGCTGAACGAGCTCGCCAGCTTGCACAACACTTATATGGTGGTG
France KX373899 ACGATGGTTGTTTGGCCTTCTGGTCATGGTTATAATATTGGTATACCTACGTATAATGCTGAACGAGCTCGCCAGCTTGCACAACACTTATATGGTGGTG
UK1 GU109335    ACGATGGTCGTTTGGCCTTCTGGTCATGGTTATAATATTGGTATACCTACGTATAATGCTGAGCGAGCTCGTCAGCTTGCACAACACTTATATGGTGGTG
UK2 KJ437447    ACTATGGTCGTTTGGCCTTCAGGTCATGGATATAATATTGGAATACCAACTTATAATGCTGAACGAGCAAGACAACTTGCTCAGCATTTGTATGGTGGTG
Korea1 JX875304 ACGATGGTTGTTTGGCCTTCTGGTCATGGTTATAATATTGGTATACCTACGTATAATGCTGACGGAGCTCGCCAGCTTGCACAGCATTTATATGGTGGTG
Korea2 JX878305 ACAATGGTTGTTTGGCCTTCTGGTCATGGTTATAATATTGGCATACCTACGTATAATGCTGAACGAGCTCGCCAGCTTGCACAACACTTATATGGTGGCG
China1 MF770715 ACGATGGTTGTTTGGCCTTCTGGTCATGGTTATAATATTGGTATACCTACGTATAATGCTGAACGAGCTCGCCAGCTTGCACAACACTTATATGGCGGTG
China2 MF036686 ACAATGGTTGTTTGGCCTTCTGGTCATGGTTATAATATTGGTATACCTACGTATAATGCTGAACGAGCTCGCCAGCTTGCACAGCATTTATATGGTGGTG
China3 MH165180 ACGATGGTTGTTTGGCCTTCTGGTCATGGTTATAATATTGGTATACCTACGTATAATGCTGAACGAGCTCGCCAGCTTGCACAACATTTATATGGTGGTG
DWV-NVN        ACAATGGTTGTTTGGCCTTCTGGTCATGGTTATAATATTGGTATACCAACATATAATGCTGAGCGAGCACGTCAGCTTGCGCAGCATTTGTATGGTGGTG
DWV-SVN        ACAATGGTTGTTTGGCCTTCTGGTCATGGTTATAATATTGGTATACCAACATATAATGCTGAGCGAGCACGTCAGCTTGCGCAGCATTTGTATGGTGGCG

                   3710      3720      3730      3740      3750      3760      3770      3780      3790      3800
               ....|....|....|....|....|....|....|....|....|....|....|....|....|....|....|....|....|....|....|
Japan AB070959 GATCATTAACTGACGAGAAGGCCAAACAATTATTTGTTCCTGCTAATCAACAAGGACCTGGTACTGTAAGTAATGGAAATCCAGTATGGGAAGTCATGCG
USA1 AY291384   GATCATTAACTGATGAGAAGGCCAAACAATTATTTGTTCCTGCTAATCAACAAGGACCTGGTAAGGTAAGTAATGGAAATCCGGTATGGGAAGTCATGCG
Chile JQ413340 GATCATTAACTGATGAGAAGGCCAAACAATTATTTGTTCCTGCTAATCAACAAGGACCTGGTAAGGTAAGTAATGGAAATCCGGTATGGGAAGTTATGCG
Italy1 AJ489744 GATCATTAACTGATGAGAAGGCCAAACAATTATTTGTTCCTGCTAATCAACAAGGACCTGGTAAGGTAAGTAATGGAAATCCGGTATGGGAAGTCATGCG
France KX373899 GATCATTAACTGATGAGAAGGCCAAACAATTATTTGTTCCTGCTAATCAACAAGGACCTGGTAAAGTAAGTAATGGAAATCCGGTGTGGGAAGTCATGCG
UK1 GU109335    GATCATTAACTGATGAGAAGGCCAAACAATTATTTGTTCCTGCTAATCAACAAGGACCTGGTAAAGTAAGTAATGGAAATCCGGTATGGGAAGTCATGCG
UK2 KJ437447    GGTCTTTGACAGATGAAAAGGCTAAGCAATTGTTTGTGCCTGCTAACCAGCAAGGACCCGGCAAAGTAAGTAACGGTAACCCTGTCTGGGAAGTAATGCG
Korea1 JX875304 GATCATTAACCGATGAGAAGGCCAAACAATTATTTGTTCCTGCTAATCAACAAGGACCTGGTAAGGTAAGTAATGGAAATCCGGTATGGGAAGTCATGCG
Korea2 JX878305 GGTCATTAACTGATGAGAAGGCCAAACAATTATTTGTTCCTGCTAATCAACAAGGACCTGGTAAGGTAAGCAATGGGAATCCAGTATGGGAAGTTATGCG
China1 MF770715 GATCATTAACTGATGAGAAGGCCAAACAATTATTTGTTCCTGCTAATCAACAAGGACCTGGTAAGGTAAGTAATGGAAATCCGGTATGGGAAGTCATGCG
China2 MF036686 GATCATTAACTGATGAGAAGGCCAAACAATTATTTGTTCCTGCTAATCAACAAGGACCTGGTAAGGTAAGTAACGGAAATCCAGTATGGGAAGTCATGCG
China3 MH165180 GATCATTAACTGATGAGAAGGCCAAACAATTATTTGTTCCTGCTAATCAACAAGGACCTGGTAAGGTAAGTAATGGAAATCCGGTATGGGAAGTCATGCG
DWV-NVN        GATCATTAACTGATGAGAAGGCCAAGCAATTGTTTGTTCCTGCTAATCAACAAGGACCCGGCACTGCAAGTAACGGAAATCCAGTATGGGAGGTCATGCG
DWV-SVN        GATCATTAACTGATGAGAAGGCTAAGCAATTATTTGTTCCTGCTAATCAACAAGGACCCGGCACTGCAAGTAACGGAAATCCAGTATGGGAGGTCATGCG

                   3810      3820      3830      3840      3850      3860      3870      3880      3890      3900
               ....|....|....|....|....|....|....|....|....|....|....|....|....|....|....|....|....|....|....|
Japan AB070959 TGCACCATTGGCAACACAACGTGCGCATGTTCAAGATTTTGAATTTATTGAAGCTATTCCAGAAGGAGAGGAGTCTCGTAATACTACAGTCTTGGATACG
USA1 AY291384   TGCACCATTGGCAACACAGCGTGCGCATATTCAAGATTTTGAATTTATTGAGGCTATTCCAGAAGGAGAGGAGTCTCGTAATACTACAGTCTTGGATACG
Chile JQ413340 TGCACCATTGGCAACACAGCGTGCGCATATTCAAGATTTTGAATTTATTGAAGCTATTCCAGAAGGAGAGGAGTCTCGTAACACCACAGTCTTGGATACG
Italy1 AJ489744 TGCACCATTGGCAACACAGCGTGCGCATATTCAAGATTTTGAATTTATTGAAGCTATTCCAGAAGGAGAGGAGTCTCGTAATACTACAGTCTTGGATACG
France KX373899 CGCACCATTGGCAACACAGCGTGCGCATATTCAAGATTTTGAATTTATTGAAGCTATTCCAGAAGGAGAGGAGTCTCGCAATACTACAGTCTTGGATACG
UK1 GU109335    CGCACCATTGGCAACACAGCGTGCGCATATTCAAGATTTTGAATTTATTGAAGCTATTCCAGAAGGAGAGGAGTCTCGTAATACTACAGTCTTGGATACG
UK2 KJ437447    CGCGCCTCTTGCAACTCAGCAAGCGCATATACAAGATTTTGAATTTGTTGAAGCTGTTCCAGAAGGCGAAGAATCACGCAACACTACGGTGCTAGATACG
Korea1 JX875304 TGCACCATTGGCAACACAGCGTGCGCATGTTCAAGATTTTGAATTTATTGAAGCTATTCCAGAAGGAGAGGAGTCTCGTAATACTACAGTCTTGGATACG
Korea2 JX878305 CGCGCCATTGGCAACACAGCGTGCGCATGTTCAAGATTTTGAGTTTATTGAAGCTATTCCAGAAGGAGAGGAGTCTCGTAATACTACAATCTTGGACACG
China1 MF770715 TGCACCATTGGCAACACAGCGTGCGCATGTTCAAGATTTTGAATTTATTGACGCTATTCCAGAAGGAGAGGAATCTCGTAATACTACAGTCTTGGATACG
China2 MF036686 TGCACCATTGGCAACACAGCGTGCGCATGTTCAAGATTTTGAATTTATTGAAGCTATTCCAGAAGGAGAAGAGTCTCGTAATACTACAGTCTTGGATACG
China3 MH165180 TGCACCATTGGCAACACAGCGTGCGCATGTTCAAGATTTTGAATTTATTGAAGCTATTCCAGAAGGAGAGGAATCTCGTAATACTACAGTCTTGGATACG
DWV-NVN        TGCACCATTGGCAACACAGCGTGCGCATGTTCAAGATTTTGAATTTATCGAAGCTATGCCAGAAGGAGAGGAATCTCGCAATACTACAGTCTTGGATACG
DWV-SVN        TGCACCATTGGCAACACAGCGTGCGCATGTTCAAGATTTTGAGTTTATCGAAGCTATGCCAGAAGGAGAGGAGTCTCGCAATACTACAGTCTTGGATACG

                   3910      3920      3930      3940      3950      3960      3970      3980      3990      4000
               ....|....|....|....|....|....|....|....|....|....|....|....|....|....|....|....|....|....|....|
Japan AB070959 ACCACTACTTTACAGTCGAGCGGATTTGGTCGCGCCTTCTTTGGAGAAGCTTTTAATGACCTTAAAACGTTAATGCGACGATATCAATTATATGGTCAAT
USA1 AY291384   ACCACTACTTTACAGTCGAGTGGATTTGGTCGCGCCTTCTTTGGAGAAGCTTTTAATGACCTTAAAACGTTAATGCGACGATATCAATTATATGGTCAAT
Chile JQ413340 ACCACTACTTTACAGTCGAGTGGATTTGGTCGCGCCTTCTTTGGAGAAGCTTTTAATGACCTTAAAACGTTAATGCGGCGATATCAATTATATGGTCAAT
Italy1 AJ489744 ACCACTACTTTACAGTCGAGTGGATTTGGTCGCGCYTTCTTTGGAGAAGCTTTTAATGAYCTTAAAACGTTAATGCGACGATATCAATTATATGGTCAAT
France KX373899 ACCACTACTTTACAGTCGAGTGGATTTGGTCGCGCTTTCTTTGGAGAAGCTTTTAATGACCTTAAAACGTTAATGCGACGATATCAACTATATGGTCAAT
UK1 GU109335    ACCACTACTTTACAGTCGAGTGGATTTGGTCGCGCCTTCTTTGGAGAAGCTTTTAATGATCTTAAAACGTTAATGCGACGATATCAACTATATGGTCAAT
UK2 KJ437447    ACAACAACGTTACAGTCTAGCGGATTTGGTCGCGCTTTCTTCGGTGAGGCATTTAACGATCTTAAGACGTTAATGCGCCGATACCAATTATATGGTCAAT
Korea1 JX875304 ACCACTACTTTACAGTCGAGTGGATTTGGTCGCGCCTTCTTTGGAGAAGCTTTTAATGACCTTAAAACGTTAATGCGACGATATCAATTATATGGTCAAT
Korea2 JX878305 ACCACTACTTTACAGTCGAGCGGATTTGGTCGCGCATTCTTTGGAGAAGCTTTTAATGACCTTAAAACGTTAATGCGACGATATCAATTATATGGTCAAT
China1 MF770715 ACCACTATTTTACAGTCGAGCGGATTTGGTCGCGCATTCTTTGGAGAAGCTTTCAATGACCTTAAAACGTTAATGCGACGATATCAATTATATGGTCAAT
China2 MF036686 ACCACTACTTTACAGTCGAGTGGATTTGGTCGCGCATTTTTTGGAGAAGCTTTTAATGATCTTAAAACGTTAATGCGACGATACCAATTATATGGCCAAT
China3 MH165180 ACCACTACTTTACAGTCGAGCGGATTTGGTCGCGCATTCTTTGGAGAAGCTTTCAATGACCTTAAAACGTTAATGCGACGATATCAATTATATGGTCAAT
DWV-NVN        ACGACTACTTTACAGTCGAGTGGATTTGGTCGCGCCTTTTTTGGAGAAGCTTTTAATGATCTTAAGACGTTAATGCGGCGATATCAATTATATGGTCAAT
DWV-SVN        ACGACTACTTTACAGTCGAGTGGATTTGGTCGCGCCTTTTTTGGAGAAGCTTTTAATGATCTTAAGACGTTAATGCGGCGATATCAATTATATGGTCAAT
```

```
                         4010      4020      4030      4040      4050      4060      4070      4080      4090      4100
                    ....|....|....|....|....|....|....|....|....|....|....|....|....|....|....|....|....|....|....|....|
Japan AB070959      TATTATTGTCCGTTACTACGGATAAGGATATTGATCATTGTATGTTTACCTTCCCTTGTTTACCACAAGGGTTAGCGTTAGACATTGGTTCTGCTGGCTC
USA1 AY291384       TATTATTGTCCGTTACTACGGATAAGGATATTGATCATTGTATGTTTACCTTCCCTTGTTTACCACAAGGGTTAGCGTTAGACATTGGTTCTGCTGGCTC
Chile JQ413340      TATTATTGTCCGTTACTACGGATAAGGATATTGATCATTGTATGTTTACCTTCCCTTGTTTACCACAAGGGTTAGCGTTAGACATTGGTTCTGCTGGCTC
Italy1 AJ489744     TATTATTGTCCGTTACTACGGATAAGGATATTGATCATTGTATGTTTACCTTCCCTTGTTTACCACAAGGGTTAGCGTTAGACATTGGTTCTGCTGGCTC
France KX373899     TATTATTGTCCGTTACTACGGATAAGGATATTGATCATTGTATGTTTACCTTCCCTTGTTTACCACAAGGGTTAGCGTTAGACATTGGTTCTGCTGGCTC
UK1 GU109335        TACTATTGTCCGTTACTACGGATAAGGATATTGATCATTGTATGTTTACCTTCCCTTGTTTACCACAAGGGTTAGCGTTAGACATTGGTTCTGCTGGCTC
UK2 KJ437447        TATTGTTATCCGTTACTACGGATAAGGATATTGATCATTGTATGTTTACCTTCCCTTGTTTACCTCAAGGGTTAGCGTTAGATATAGGTTCGGCTGGATC
Korea1 JX875304     TATTATTGTCCGTTACTACGGATAAGGATATTGATCATTGTATGTTTACCTTCCCTTGTTTACCACAAGGGTTAGCGTTAGACATTGGTTCAGCTGGCTC
Korea2 JX878305     TATTATTGTCCGTTACTACGGATAAGGATATTGATCATTGTATGTTTACCTTCCCTTGTTTACCACAAGGGTTGGCGTTAGATATTGGTTCTGCTGGCTC
China1 MF770715     TATTATTGTCCGTTACTACGGATAAGAATATTGATCATTGTATGTTTACCTTCCCTTGTTTACCACAAGGGCTAGCGTTAGATATCGGTTCTGCTGGCTC
China2 MF036686     TGTTATTGTCCGTTACTACGGATAAGGATATTGATCATTGTATGTTTACCTTCCCTTGTTTACCACAAGGGTTAGCGTTAGACATTGGTTCTGCTGGCTC
China3 MH165180     TATTATTGTCCGTTACTACGGATAAGGATATTGATCATTGTATGTTTACTTTCCCTTGTTTACCACAAGGGCTAGCGTTAGATATTGGTTCTGCTGGCTC
DWV-NVN             TATTATTGTCTGTTACTACAGATAAAGATATTGATCATTGTATGTTTACTTTCCCTTGTTTACCACAAGGGTTGGCATTAGACATAGGGTCTGCCGGAGC
DWV-SVN             TATTATTGTCTGTTACTACAGATAAAGATATTGATCATTGTATGTTTACTTTCCCTTGTTTACCACAAGGGTTAGCATTAGACATAGGGTCTGCCGGAGC
```

```
                         4110      4120      4130      4140      4150      4160      4170      4180      4190      4200
                    ....|....|....|....|....|....|....|....|....|....|....|....|....|....|....|....|....|....|....|....|
Japan AB070959      TCCACATGAAATCTTTAATAGATGTCGTGATGGTATTATACCATTAATTGCATCTGGATATAGATTTTATAGAGGAGAYTTGCGTTATAAGATTGTTTTT
USA1 AY291384       TCCACATGAAATCTTTAATAGATGTCGTGATGGTATTATACCATTAATTGCATCTGGATATAGATTTTATAGAGGAGATTTGCGTTATAAGATTGTTTTT
Chile JQ413340      TCCACATGAAATTTTTAATAGATGCCGTGATGGTATTATACCATTAATTGCATCTGGATATAGATTTTATAGAGGAGATTTGCGTTATAAGATTGTTTTT
Italy1 AJ489744     TCCACATGAAATCTTTAATAGATGTCGTGATGGTATTATACCATTAATTGCATCTGGATATAGATTTTATAGAGGAGATTTGCGTTATAAGATTGTTTTT
France KX373899     TCCACATGAAATCTTTAATAGATGTCGTGATGGTATTATACCATTAATTGCATCTGGATATAGATTTTATAGAGGAGATTTGCGTTATAAGATCGTTTTT
UK1 GU109335        TCCACATGAAATCTTTAATAGATGTCGTGATGGTATTATACCATTAATTGCATCTGGATATAGATTTTATAGAGGAGATTTGCGTTATAAGATTGTTTTT
UK2 KJ437447        TCCTCATGAAATATTCAATCGCTGCCGTGATGGTATCATTCCACTGATAGCGTCAGGGTATCGGTTTTATCGAGGCGATTTACGGTTCAAAATTGTTTTC
Korea1 JX875304     CCCACATGAAATCTTTAATAGATGTCGTGATGGTATTATACCATTGATTGCGTCTGGATATAGATTTTATAGAGGAGATTTGCGTTATAAGATTGTTTTT
Korea2 JX878305     TCCACATGAAATCTTTAATAGATGTCGTGATGGCATTATACCATTAATTGCATCTGGATATAGATTTTATAGAGGAGATTTGCGTTATAAGATTGTTTTT
China1 MF770715     TCCACATGAAATCTTTAATAGATGTCGTGATGGTATTATACCATTAATTGCATCTGGATATAGATTTTATAGAGGAGATTTACGTTATAAAATTGTTTTT
China2 MF036686     TCCACATGAAATCTTTAATAGATGTCGTGATGGTATTATACCATTAATTGCATCTGGATATAGATTTTATAGAGGAGATTTGCGTTATAAGATCGTTTTT
China3 MH165180     TCCACATGAAATCTTTAATAGATGTCGTGATGGTATTATACCATTAATTGCATCTGGATATAGATTTTATAGAGGAGATTTACGTTATAAAATTGTTTTT
DWV-NVN             GCCTCATGAGATCTTTAACAGATGCCGAGATGGTATTATACCATTAATTGCGTCTGGATATAGATTTTATAGAGGCGATTTACGTTATAAGATTGTTTTC
DWV-SVN             GCCTCATGAGATCTTTAACAGATGCCGAGACGGTATTATACCATTAATTGCGTCTGGTTATAGATTTTATAGAGGCGATTTACGTTATAAGATCGTTTTC
```

```
                         4210      4220      4230      4240      4250      4260      4270      4280      4290      4300
                    ....|....|....|....|....|....|....|....|....|....|....|....|....|....|....|....|....|....|....|....|
Japan AB070959      CCAAGTAATGTTAATAGCAACATTTGGGTACAACATCGACCGGATCGTAGRCTGGAAGGATGGTCTGCGGCTAAGATTGTAAATTGTGATGCTGTGTCTA
USA1 AY291384       CCAAGTAATGTTAATAGCAACATTTGGGTACAACATCGACCGGATCGTAGACTGGAAGGATGGTCCGCGGCTAAGATTGTAAATTGTGATGCTGTGTCTA
Chile JQ413340      CCAAGTAATGTTAATAGCAACATTTGGGTACAACATCGACCGGATCGTAGACTGGAAGGATGGTCCGCGGCTAAGATTGTAAATTGTGATGCTGTATCTA
Italy1 AJ489744     CCAAGTAATGTTAATAGCAACATTTGGGTACAACATCGACCGGATCGTAGACTGGAAGGATGGTCCGCGGCTAAGATTGTAAATTGTGATGCTGTGTCTA
France KX373899     CCAAGTAATGTTAATAGCAATATTTGGGTACAACATCGACCGGATCGTAGATTGGAAGGATGGTCCGCGGCTAAGATTGTAAATTGTGATGCTGTGACTA
UK1 GU109335        CCAAGTAATGTTAATAGCAATATTTGGGTACAACATCGACCGGATCGTAGACTGGAAGGATGGTCCGCGGCTAAGATTGTAAATTGTGATGCTGTGTCTA
UK2 KJ437447        CCAAGTAATGTTAATAGCAATATTTGGGTACAACACCGACCAGATCGTAGACTGAAAGGATGGTCTGAAGCGAAAATAGTAAACTGTGATGCTGTATCTA
Korea1 JX875304     CCAAGTAATGTTAATAGCAATATTTGGGTACAACATCGACCGGATCGTAGATTGGAAGGATGGTCTGCGGCCAAGATTGTAAATTGTGATGCTGTGTCTA
Korea2 JX878305     CCAAGTAATGTTAACAGCAACATTTGGGTACAACATCGACCGGATCGTAGATTGGAAGGATGGTCTGCGGCTAAGATTGTAAATTGCGATGCTGTGTCTA
China1 MF770715     CCAAGTAATGTTAATAGCAATATTTGGGTGCAACATCGACCGGATCGTAGACTGGAAGGATGGTCTGCGGCTAAGATTATAAATTGCGATGCTGTGTCTA
China2 MF036686     CCAAGTAACGTTAATAGCAATATTTGGGTACAACATCGACCGGATCGTAGACTAGAAGGATGGTCTGCGGCTAAGATTGTAAATTGTGATGCTGTGTCTA
China3 MH165180     CCAAGTAATGTTAATAGCAACATTTGGGTACAACATCGACCGGATCGTAGACTGGAAGGATGGTCTGCGGCTAAGATTATAAATTGTGATGCTGTGTCTA
DWV-NVN             CCAAGTAATGTTAATAGCAATATTTGGGTACAACACCGACCGGATCGTAGACTGACAGGATGGTCTGCGGCTAAGATTGTTAATTGCGATGCTGTGTCTA
DWV-SVN             CCAAGTAATGTTAATAGCAATATTTGGGTACAACATCGACCGGATCGCAGACTAACAGGATGGTCTGCGGCTAAGATTGTTAATTGCGATGCTGTGTCTA
```

```
                         4310      4320      4330      4340      4350      4360      4370      4380      4390      4400
                    ....|....|....|....|....|....|....|....|....|....|....|....|....|....|....|....|....|....|....|....|
Japan AB070959      CTGGTCAAGGGGTGTATAATCATGGTTATGCTAGTCACATTCAAATCACGCGTGTAAATAATGTTATAGAATTGGAAGTCCCATTTTATAATGCTACTTG
USA1 AY291384       CTGGTCAAGGGGTGTATAATCATGGTTATGCTAGTCACATTCAAATCACGCGTGTAAATAATGTTATAGAATTGGAAGTTCCATTTTATAATGCTACTTG
Chile JQ413340      CTGGTCAAGGGGTGTATAATCATGGTTATGCTAGTCACATTCAAATCACGCGTGTAAATAATGTTATAGAATTGGAAGTTCCATTTTATAATGCTACTTG
Italy1 AJ489744     CTGGTCAAGGGGTGTATAATCATGGTTATGCTAGTCACATTCAAATCACGCGTGTAAATAATGTTATAGAATTGGAAGTTCCATTTTATAATGCTACTTG
France KX373899     CTGGTCAAGGGGTGTATAATCATGGTTATGCTAGTCACATACAAATCACGCGTGTAAATAATGTTATAGAATTGGAAGTTCCATTTTATAATGCGACTTG
UK1 GU109335        CTGGTCAAGGGGTGTATAATCATGGTTATGCTAGTCACATTCAAATCACGCGTGTAAATAATGTTATAGAATTGGAAGTTCCATTTTATAATGCTACTTG
UK2 KJ437447        CTGGACAAGGCGTTTATAATCATGGATATGCTAGTCATATTCAGATTACGCGTGTAAATAATGTTATAGAATTGGAAGTCCCGTTTTATAACGCTACGTG
Korea1 JX875304     CTGGTCAAGGGGTGTATAATCATGGTTATGCTAGTCACATTCAAATCACGCGTGTAAATAATGTTATAGAATTGGAAGTTCCATTTTATAATGCTACTTG
Korea2 JX878305     CTGGTCAAGGGGTGTATAATCATGGTTATGCTAGTCACATTCAAATCACGCGTGTAAATAATGTTATAGAATTGGAAGTTCCATTTTATAATGCTACTTG
China1 MF770715     CTGGTCAAGGGGTGTATAATCATGGTTATGCTAGTCACATTCAAATTACGCGTGTAAATAATGTTATAGAATTGGAAGTTCCATTTTATAATGCTACTTG
China2 MF036686     CTGGTCAAGGGGTGTATAATCATGGTTATGCTAGTCACATTCAAATTACGCGTGTAAATAATGTTATAGAATTGGAAGTTCCATTTTATAATGCTACTTG
China3 MH165180     CTGGTCAAGGGGTGTATAATCATGGTTATGCTAGTCACATTCAAATTACGCGTGTAAATAATGTTATAGAATTGGAAGTTCCATTCTATAATGCTACTTG
DWV-NVN             CTGGTCAAGGAGTATACAATCATGGTTACGCTAGTCACATTCAAATTACGCGTGTAAATAATGTTATAGAATTAGAAGTTCCATTTTATAATGCAACTTG
DWV-SVN             CTGGTCAAGGAGTATACAATCATGGTTACGCTAGTCACATTCAAATTACGCGTGTAAATAATGTTATAGAATTAGAAGTTCCATTTTATAATGCAACTTG
```

```
                           4410      4420      4430      4440      4450      4460      4470      4480      4490      4500
                   ....|....|....|....|....|....|....|....|....|....|....|....|....|....|....|....|....|....|....|....|
Japan AB070959     TTACAATTATTTACAGGCGTTTAATGCGTCTAGCGCTGCATCTAGTTATGCAGTATCTTTAGGAGAAATATCGGTTGGTTTTCAAGCTACAAGTGATGAT
USA1 AY291384      TTATAATTATTTACAGGCGTTTAATGCGTCTAGCGCTGCATCTAGTTATGCAGTATCTTTAGGAGAAATATCGGTTGGTTTTCAAGCTACAAGTGATGAT
Chile JQ413340     TTATAATTATTTACAGGCGTTTAATGCGTCTAGCGCTGCATCTAGTTATGCAGTATCTTTAGGAGAAATATCGGTTGGTTTTCAAGCTACAAGTGATGAT
Italy1 AJ489744    TTATAATTATTTACAGGCGTTTAATGCGTCTAGCGCTGCATCTAGTTATGCAGTATCTTTAGGAGAAATATCGGTTGGTTTTCAAGCTACAAGTGATGAT
France KX373899    TTATAATTATTTACAGGCGTTTAATGCGTCTAGTGCTGCATCTAGCTATGCAGTATCTTTAGGAGAAATATCGGTTGGTTTTCAAGCTACAAGTGATGAT
UK1 GU109335       TTATAATTATTTACAGGCGTTTAATGCGTCTAGCGCTGCATCTAGTTATGCAGTATCTTTAGGAGAAATATCGGTTGGTTTTCAAGCTACAAGTGATGAT
UK2 KJ437447       CTATAATTATTTGCAAGCGTTTAACCCATCTAGTGCAGCGTCGAGTTATGCCGTTTCGCTCGGAGAGATTTCGGTTGGTTTTCAAGCTACTAGTGATGAC
Korea1 JX875304    TTATAATTATTTGCAGGCGTTTAATGCGTCTAGCGCTGCATCTAGTTATGCAGTATCTTTAGGAGAAATATCGGTTGGTTTCCAGGCTACAAGTGATGAT
Korea2 JX878305    TTATAATTATTTACAGGCGTTTAATGCGTCTAGCGCTGCATCTAGTTATGCAGTATCTTTAGGAGAAATATCGGTTGGTTTTCAAGCTACAAGTGATGAT
China1 MF770715    TTATACTTATTTACAGGCGTTTAATGCGTCTAGCGCTGCATCTAGTTATGCAGTATCTTTAGGAGAAATATCTGTTGGTTTTCAAGCTACAAGTGATGAT
China2 MF036686    TTATAATTATTTACAGGCGTTTAATGCGTCTAGCGCTGCATCTAGTTATGCAGTATCTTTAGGAGAAATATCGGTTGGTTTTCAAGCTACAAGTGATGAT
China3 MH165180    TTATAATTATTTACAGGCGTTTAATGCGTCTAGCGCTGCATCTAGTTATGCAGTATCTTTAGGAGAAATATCTGTTGGTTTTCAAGCTACAAGTGATGAT
DWV-NVN            CTATAATTATTTGCAGGCGTTTAATGCGTCTAGTGCAGCGTCTAGCTATGCAGTGTCTCTAGGAGAAATATCGGTTGGTTTTCAAGCCACAAGTGATGAT
DWV-SVN            TTATAATTATTTGCAGGCGTTTAACGCGTCTAGTGCAGCGTCTAGCTATGCAGTGTCTCTAGGAGAAATATCGGTTGGTTTTCAAGCCACAAGTGATGAT

                           4510      4520      4530      4540      4550      4560      4570      4580      4590      4600
                   ....|....|....|....|....|....|....|....|....|....|....|....|....|....|....|....|....|....|....|....|
Japan AB070959     ATTGCATCTATTGTYAACAAACCTGTCACTATTTATTATAGTATAGGAGATGGTATGCAATTTTCTCAGTGGGTTGGATATCAACCGATGATGATCCTAG
USA1 AY291384      ATTGCATCTATTGTTAACAAACCTGTTACTATTTATTATAGTATTGGAGATGGTATGCAATTTTCTCAGTGGGTTGGATATCAACCGATGATGATCCTAG
Chile JQ413340     ATTGCATCTATTGTTAACAAACCTGTTACTATTTATTATAGTATTGGAGATGGTATGCAATTTTCCCAGTGGGTTGGATATCAACCGATGATGATTCTAG
Italy1 AJ489744    ATTGCATCTATTGTTAACAAACCTGTTACTATTTATTATAGTATTGGAGATGGTATGCAATTTTCTCAGTGGGTTGGATATCAACCGATGATGATCCTAG
France KX373899    ATTGCATCTATTGTTAACAAACCTGTTACTATTTATTATAGTATTGGAGATGGTATGCAATTTTCTCAGTGGGTTGGATATCAACCGATGATGATCCTAG
UK1 GU109335       ATTGCATCTATTGTTAACAAGCCTGTTACTATTTATTATAGTATTGGAGATGGTATGCAATTTTCTCAGTGGGTTGGATATCAACCGATGATGATCCTAG
UK2 KJ437447       ATTGCAGCCATAGTTAATAAACCTGTAACTATATATTACAGTATTGGCGATGGTATGCAGTTTTCGCAGTGGGTTGGTTATCAACCAATGATGATTCTAG
Korea1 JX875304    ATTGCATCTATTGTTAATAAACCTGTCACTATTTATTATAGTATAGGAGATGGTATGCAATTTTCTCAGTGGGTTGGATATCAACCGATGATGATCCTAG
Korea2 JX878305    ATTGCATCTATTGTTAATAAACCTGTTACTATTTATTATAGTATAGGAGATGGTATGCAATTTTCTCAGTGGGTTGGATATCAACCGATGATGATCCTAG
China1 MF770715    ATTGCAACTATTGTTAACAAACCTGTTACTATTTATTATAGTATAGGAGATGGTATGCAATTTTCTCAGTGGGTTGGATATCAACCGATGATGATCCTAG
China2 MF036686    ATTGCATCCATTGTTAACAAACCTGTTTCTATTTATTATAGTATAGGAGATGGTATGCAATTTTCTCAGTGGGTTGGATATCAACCGATGATGATTCTAG
China3 MH165180    ATTGCATCTATTGTTAACAAACCTGTTACTATTTATTATAGTATAGGAGATGGTATGCAATTTTCTCAGTGGGTTGGGTATCAACCGATGATGATCCTAG
DWV-NVN            ATTGCGTCTATTGTCAATAAGCCTGTGTCTATTTATTATAGTATAGGTGATGGTATGCAATTCTCACAGTGGGTTGGATATCAGCCGATGATGATCCTAG
DWV-SVN            ATTGCGTCTATTGTTAATAAGCCTGTTTCTATTTATTATAGTATAGGTGATGGTATGCAATTCTCACAGTGGGTTGGATATCAGCCGATGATGATCTTAG

                           4610      4620      4630      4640      4650      4660      4670      4680      4690      4700
                   ....|....|....|....|....|....|....|....|....|....|....|....|....|....|....|....|....|....|....|....|
Japan AB070959     ACCAGCTTCCTGCACCAGTAGTAAGGGCCGTGCCTGAGGGCCCTATTGCGAAGATTAAAAACTTCTTCCATCAAACAGCTGACGAGGTTAGAGAAGCTCA
USA1 AY291384      ATCAGCTTCCTGCACCAGTAGTAAGGGCCGTGCCTGAGGGCCCTATTGCGAAGATTAAAAACTTCTTCCATCAAACAGCCGACGAAGTTAGAGAAGCTCA
Chile JQ413340     ACCAGCTTCCCGCACCAGTAGTAAGGGCCGTGCCTGAGGGCCCTATTGCGAAGATTAAAAACTTCTTCCATCAAACAGCCGATGAAGTAAGAGAAGCTCA
Italy1 AJ489744    ATCAGCTYCCTGCACCAGTAGTAAGGGCCGTGCCTGAGGGCCCTATTGCGAAGATTAAAAACTTCTTCCATCAAACAGCCGAYGAAGTTAGAGAAGCTCA
France KX373899    ACCAGCTTCCTGCACCAGTAGTAAAGGCCGTGCCTGAGGGCCCTATTGCGAAGATTAAAAACTTCTTCCATCAAACAGCTGACGAAGTTAGAGAAGTTCA
UK1 GU109335       ACCAGCTTCCTGCACCAGTCGTAAGGGCCGTGCCTGAGGGCCCTATTGCGAAGATTAAAAACTTCTTCCATCAAACAGCCGATGAAGTTAGAGAAGCTCA
UK2 KJ437447       ATCAATTGCCAGCACCAGTAGTTAGGGCAGTGCCTGAGGGCCCTATAGCGAAGATAAAGAACTTTTTCCACCAAACGGCAGATGAAGTTCGAGAAGCTCA
Korea1 JX875304    ACCAGCTTCCTGCACCAGTAGTAAGGGCCGTGCCTGAGGGCCCTATTGCGAAGATTAAAAACTTCTTCCATCAAACAGCTGATGAAGTTAGAGAAGCTCA
Korea2 JX878305    ACCAGCTTCCTGCACCAGTAGTAAGGGCCGTGCCTGAGGGCCCTATTGCGAAGATTAAAAACTTCTTCCATCAAACAGCTGACGAAGTTAGAGAAGCTCA
China1 MF770715    ATCAGCTCCCTGCACCAGTAGTAAGGGCCGTGCCTGAGGGCCCTATTGCGAAGATTAAAAACTTCTTCCATCAAACAGCTGATGAAGTTAGAGAAGTTCA
China2 MF036686    ACCAGCTCCCTGCACCAGTAGTAAGGGCCGTGCCTGAGGGCCCTATTGCGAAGATTAAAAACTTCTTCCATCAAACAGCTGATGAAGTTAGAGAAGCTCA
China3 MH165180    ACCGGCTCCCTGCACCAGTAGTAAGGGCCGTGCCTGAGGGCCCTATTGCGAAGATTAAGAACTTCTTCCATCAAACAGCTGATGAAGTTAGAGAAGCTCA
DWV-NVN            ACCAGCTCCCTGCACCAGTGGTAAGGGCCATGCCTGAGGGCCCTATTGCGAAGATTAAAAACTTCTTCCATCAAACAGCTGATGAAGTTAGAGAAGCTCA
DWV-SVN            ATCAGCTCCCTGCACCAGTGGTAAGGGCCATGCCTGAGGGCCCTATTGCGAAGATTAAAAACTTCTTCCATCAAACAGCTGATGAAGTTAGAGAAGCTCA

                           4710      4720      4730      4740      4750      4760      4770      4780      4790      4800
                   ....|....|....|....|....|....|....|....|....|....|....|....|....|....|....|....|....|....|....|....|
Japan AB070959     GGCAGCAAAGATGCGTGAAGATATGGGTATGGTTGTCCAAGATGTTATTGGAGAACTTAGCCAGGCCATACCGGATCTTCAACAACCAGAGGTTCAAGCA
USA1 AY291384      GGCAGCAAAGATGCGTGAAGATATGGGTATGGTTGTCCAAGATGTTATTGGAGAACTTAGCCAGGCCATACCGGATCTTCAACAACCGGAGGTTCAAGCA
Chile JQ413340     GGCAGCAAAGATGCGTGAAGATATGGGTATGGTTGTCCAAGATGTTATTGGAGAACTTAGCCAGGCCATACCGGATCTTCAACAACCAGAGGTTCAAGCA
Italy1 AJ489744    GGCAGCAAAGATGCGTGAAGATATGGGTATGGTTGTCCAAGATGTTATTGGAGAACTTAGCCAGGCYATACCGGATCTTCAACAACCGGAGGTTCAAGCA
France KX373899    GGCAGCAAAGATGCGTGAAGATATGGGTATGGTTGTCCAAGATGTTATTGGAGAACTTAGCCAAGCCATACCGGATCTTCAACAACCGGAGGTTCAAGCA
UK1 GU109335       GGCAGCAAAGATGCGTGAAGACATGGGTATGGTTGTCCAAGATGTTATTGGAGAACTTAGCCAGGCCATACCGGATCTTCAACAACCGGAGGTTCAAGCA
UK2 KJ437447       GGCCGCAAAGATGCGTGAAGATATGGGTATAGTAGTTCAAGACGTTATAGGAGAGTTAAGTCAGGCTATACCCGATCTTCAACAACCGGAAGTTCAAGCG
Korea1 JX875304    GGCAGCAAAGATGCGTGAGGATATGGGTATGGTTGTCCAAGATGTTATTGGAGAACTTAGCCAGGCCATACCAGATCTTCAACAACCAGAGGTTCAAGCA
Korea2 JX878305    GGCAGCAAAGATGCGTGAAGATATGGGTATGGTTGTCCAAGATGTTATTGGAGAACTTAGCCAGGCCATACCGGATCTTCAACAACCAGAGGTTCAAGCA
China1 MF770715    GGCAGCAAAGATGCGTGAAGATATGGGTATAGTTGTCCAAGATGTTATTGGAGAACTTAGCCAGGCCATACCGGATCTTCAACAACCAGAGGTTCAAGCA
China2 MF036686    GGCAGCAAAGATGCGTGAAGATATGGGTATAGTTGTCCAAGATGTTATTGGAGAACTTAGCCAGGCCATACCGGATCTTCAACAACCAGAGGTTCAAGCA
China3 MH165180    GGCAGCAAAGATGCGTGAAGATATGGGTATAGTTGTCCAAGATGTTATTGGAGAACTTAGCCAGGCCATACCGGATCTTCAACAACCAGAGGTTCAAGCA
DWV-NVN            GGCAGCAAAGATGCGTGAAGATATGGGTATGGTTGTCCAAGATGTTATTGGAGAACTTAGCCAGGCCATACCGGATCTTCAACAACCAGAAGTTCAAGCG
DWV-SVN            GGCAGCAAAGATGCGTGAAGATATGGGTATGGTTGTCCAAGATGTTATTGGAGAACTTAGCCAGGCCATACCGGATCTTCAACAACCAGAAGTTCAAGCA
```

```
                      4810      4820      4830      4840      4850      4860      4870      4880      4890      4900
                 ....|....|....|....|....|....|....|....|....|....|....|....|....|....|....|....|....|....|....|....|
Japan AB070959   AATGTCTTCTCACTGGTGTCTCAGTTAGTGCATGCTATTATAGGTACTAGTTTAAAGACAGTTGCTTGGGCGATTGTTTCGATTTTTGTGACTTTAGGAT
USA1 AY291384    AATGTCTTCTCACTGGTGTCTCAGTTAGTGCATGCTATTATAGGTACTAGTTTGAAGACAGTAGCTTGGGCGATTGTTTCGATTTTTGTGACTTTAGGAT
Chile JQ413340   AATGTCTTCTCACTGGTGTCTCAGTTAGTGCATGCTATTATAGGTACTAGTTTGAAGACAGTTGCTTGGGCGATTGTTTCGATTTTTGTGACTTTAGGAT
Italy1 AJ489744  AATGTCTTCTCACTGGTGTCTCAGTTAGTGCATGCTATTATAGGTACTAGTTTGAAGACAGTTGCTTGGGCGATTGTTTCGATTTTTGTGACTTTAGGAT
France KX373899  AATGTCTTCTCACTGGTGTCTCAGTTAGTGCATGCTATTATAGGTACTAGTTTGAAGACAGTTGCTTGGGCGATTGTTTCGATTTTTGTGACTTTAGGAT
UK1 GU109335     AATGTCTTCTCACTGGTGTCTCAGTTAGTGCATGCTATTATAGGTACTAGTTTGAAGACAGTTGCTTGGGCGATTGTTTCGATTTTTGTGACTTTAGGAT
UK2 KJ437447     AATGTTTTTTCTCTGGTGTCACAGTTAGTGCATGCTATCATCGGTACTAGTCTTAAGACAGTTGCTTGGGCGATTGTTTCGATTTTTGTAACTTTAGGTT
Korea1 JX875304  AATGTCTTCTCACTGGTGTCTCAGTTAGTGCATGCTATTATAGGTACTAGTTTGAAGACAGTTGCCTGGGCGATTGTTTCGATTTTTGTGACTTTAGGAT
Korea2 JX878305  AATGTCTTTTCACTGGTGTCTCAGTTAGTGCATGCTATTATAGGTACTAGTTTGAAGACAGTTGCTTGGGCGATTGTTTCGATTTTTGTGACTTTAGGAT
China1 MF770715  AATGTCTTCTCACTGGTGTCGCAGTTAGTGCATGCTATTATAGGTACTAGTTTGAAGACAGTTGCTTGGGCGATTGTTTCGATTTTTGTGACTTTAGGAT
China2 MF036686  AATGTCTTCTCACTGGTATCTCAGTTAGTGCATGCTATTATAGGTACTAGTTTGAAGACAGTTGCTTGGGCGATTGTTTCGATTTTTGTGACTTTAGGAT
China3 MH165180  AATGTCTTCTCACTGGTGTCTCAGTTAGTGCATGCTATTATAGGTACTAGTTTGAAGACAGTTGCTTGGGCGATTGTTTCGATTTTTGTGACTTTAGGAT
DWV-NVN          AATGTCTTTTCACTGGTATCTCAGTTAGTGCATGCTATTATAGGTACCAGTTTGAAGACAGTTGCTTGGGCAATTGTTTCGATTTTTGTGACTTTAGGAT
DWV-SVN          AATGTCTTTTCGCTGGTATCTCAGTTAGTGCATGCTATTATAGGTACCAGTTTGAAGACAGTCGCTTGGGCAATTGTTTCGATTTTTGTGACCTTAGGAT
```

```
                      4910      4920      4930      4940      4950      4960      4970      4980      4990      5000
                 ....|....|....|....|....|....|....|....|....|....|....|....|....|....|....|....|....|....|....|....|
Japan AB070959   TAATTGGACGCGAAATGATGCATTCAGTTATTACTGTAGTCAAGCGGTTACTTGAGAAATATCACTTGGCGACGCAACCCCAGGAATCCGCCAGTTCAAG
USA1 AY291384    TAATTGGACGCGAAATGATGCATTCAGTTATTACTGTAGTCAAGCGGTTACTTGAGAAATATCACTTGGCGACGCAACCCCAGGAATCCGCCAGTTCAAG
Chile JQ413340   TAATTGGACGCGAAATGATGCATTCAGTTATTACCGTAGTCAAGCGGTTACTTGAGAAATATCACTTGGCGACGCAACCCCAGGAATCCGCCAGTTCAAG
Italy1 AJ489744  TAATTGGACGCGAAATGATGCATTCAGTTATTACTGTAGTCAAGCGGTTACTTGAGAAATATCACTTGGCGACGCAACCCCAGGAATCCGCCAGTTCAAG
France KX373899  TAATTGGACGCGAAATGATGCATTCAGTTATTACTGTAGTCAAGCGGTTACTTGAGAAATATCACTTGGCGACGCAACCCCAGGAATCCGCCAGTTCAAG
UK1 GU109335     TAATTGGACGCGAAATGATGCATTCAGTTATTACTGTAGTCAAGCGGTTACTTGAGAAATATCACTTGGCGACGCAACCCCAGGAATCCGCCAGTTCAAG
UK2 KJ437447     TGATTGGACGTGAAATGATGCATTCAGTCATAACTGTAGTTAAGCGGTTATTAGAAAAATATCACTTGGCGACGCAACCCCAGGAATCCGCCAATTCAGG
Korea1 JX875304  TAATTGGACGCGAAATGATGCATTCAGTTATTACTGTAGTCAAGCGGTTACTTGAGAAATATCACTTGGCGACGCAACCCCAGGAATCCGCCAGTTCAGG
Korea2 JX878305  TAATTGGACGCGAAATGATGCATTCAGTTATTACTGTAGTCATGCGGTTACTTGAGAAATATCACTTGGCGACGCAACCCCAGGAATCCGCCAATTCAAG
China1 MF770715  TAATTGGACGCGAAATGATGCATTCAGTCATTACTGTAGTCAAGCGGTTACTTGAGAAATATCACTTGGCGACGCAACCCCAGGAATCCGCCAGTTCAGG
China2 MF036686  TAATTGGACGCGAAATGATGCATTCAGTTATTACTGTAGTCAAGCGGTTACTTGAAAAATATCACTTGGCGACGCAACCCCAGGAATCCGCCAGTTCAGG
China3 MH165180  TAATTGGACGCGAAATGATGCATTCAGTTATTACTGTAGTCAAGCGGTTACTTGAGAAATATCACTTGGCGACGCAACCCCAGGAATCCGCCAGTTCAGG
DWV-NVN          TAATTGGACGTGAAATGATGCATTCAGTTATTACTGTGGTCAAGCAGTTACTTGAGAAATATCACTTGGCGACGCAACCCCAGGAATCCGCCAGTTCAGG
DWV-SVN          TAATTGGACGTGAAATGATGCATTCAGTTATTACTGTGGTCAAGCGGTTACTTGAGAAATATCACTTGGCGACGCAACCCCAGGAATCCGCCAGTTCAGG
```

```
                      5010      5020      5030      5040      5050      5060      5070      5080      5090      5100
                 ....|....|....|....|....|....|....|....|....|....|....|....|....|....|....|....|....|....|....|....|
Japan AB070959   TACTGTTATATCTGCCGTTCCAGAAGCTCCCAATGCTGAAGCGGAGGAGGCAAGTGCTTGGGTATCCATTATTTATAATGGTGTGTGTAATATGCTTAAT
USA1 AY291384    TACTGTTATATCTGCCGTTCCAGAAGCTCCCAATGCTGAAGCGGAGGAGGCAAGTGCTTGGGTATCCATTATTTATAATGGTGTGTGTAATATGCTTAAT
Chile JQ413340   TGCTGTTATATCTGCCGTTCCAGAAGCTCCCAATGCTGAAGCGGAGGAGGCAAGTGCTTGGGTATCCATTATTTATAATGGTGTGTGTAATATGCTTAAT
Italy1 AJ489744  TACTGTTATATCTGCCGTTCCAGAAGCTCCCAATGCTGAAGCGGAGGAGGCAAGTGCTTGGGTATCCATTATTTATAATGGTGTGTGTAATATGCTTAAT
France KX373899  TACTATTATATCAGCCGTTCCAGAAGCTCCCAATGCTGAAGCGGAGGAGGCAAGTGCTTGGGTATCCATTATTTATAATGGTGTGTGTAATATGCTTAAT
UK1 GU109335     TACTGTTATACCTGCCGTTCCAGAAGCTCCCAATGCTGAAGCGGAGGAGGCAAGTGCTTGGGTATCCATTATTTATAATGGTGTGTGTAATATGCTTAAT
UK2 KJ437447     TACGGTTATTTCCGCTGTTCCAGAAGCACCTAATGCTGAAGCAGAGGAGGCTAGTGCCTGGGTATCCATTATTTATAATGGTGTGTGTAATATGTTGAAT
Korea1 JX875304  TACTGTTATATCCGCCGTTCCAGAAGCTCCCAATGCTGAAGCGGAGGAGGCAAGTGCTTGGGTATCCATTATTTATAATGGTGTGTGTAATATGCTTAAT
Korea2 JX878305  TACTGTTATATCTGCCGTTCCAGAAGCTCCCAATGCTGAAGCGGAGGAGGCAAGTGCTTGGGTATCCATTATTTATAATGGTGTGTGTAATATGCTTAAT
China1 MF770715  TACTGTTATATCTGCCGTTCCAGAAGCTCCCAATGCTGAAGCGGAGGAGGCAAGTGCTTGGGTATCCATTATTTATAATGGTGTGTGTAATATGCTTAAT
China2 MF036686  TACTGTTATATCTGCCGTTCCAGAAGCTCCCAATGCTGAAGCGGAGGAGGCAAGTGCTTGGGTATCCATTATTTATAATGGTGTGTGTAATATGCTTAAT
China3 MH165180  TACTGTTGTATCTGCCGTTCCAGAAGCTCCCAATGCTGAAGCGGAGGAGGCAAGTGCTTGGGTATCCATTATTTATAATGGTGTGTGTAATATGCTTAAT
DWV-NVN          TACTGTTATATCTGCCGTTCCAGAAGCTCCCAATGCTGAAGCGGAGGAGGCAAGTGCTTGGGTATCCATTATTTATAATGGTGTGTGTAATATGCTCAAT
DWV-SVN          TACTGTTATATCTGCCGTTCCAGAAGCTCCCAATGCTGAAGCGGAGGAGGCAAGTGCTTGGGTATCCATTATTTATAACGGTGTGTGTAATATGCTCAAT
```

```
                      5110      5120      5130      5140      5150      5160      5170      5180      5190      5200
                 ....|....|....|....|....|....|....|....|....|....|....|....|....|....|....|....|....|....|....|....|
Japan AB070959   GTGGCTGCTCAAAAACCGAAACAATTTAAAGATTGGGTAAAATTAGCTACTGTAGATTTTAGTAATAATTGTAGAGGTAGTAATCAGGTATTTGTATTTT
USA1 AY291384    GTGGCTGCTCAAAAACCGAAACAATTTAAAGATTGGGTAAAATTAGCTACTGTAGATTTTAGTAATAATTGTAGAGGTAGTAATCAGGTATTTGTATTTT
Chile JQ413340   GTGGCTGCTCAAAAACCGAAACAATTTAAAGATTGGGTAAAATTAGCTACTGTAGATTTTAGTAATAATTGTAGAGGTAGTAACCAGGTATTTGTATTTT
Italy1 AJ489744  GTRGCTGCTCAAAAACCGAAACAATTTAAAGATTGGGTAAAATTAGCTACTGTAGATTTTAGTAATAATTGTAGAGGTAGTAACCAGGTATTTGTATTTT
France KX373899  GTGGCTGCTCAAAAACCGAAACAATTTAAAGATTGGGTAAAATTAGCTACTGTAGATTTTAGTAATAATTGTAGAGGTAGTAATCAGGTATTTGTATTTT
UK1 GU109335     GTGGCCGCTCAAAAACCGAAACAATTTAAAGATTGGGTAAAATTAGCTACCGTAGATTTTAGTAATAATTGTAGAGGTAGTAACCAGGTATTTGTATTTT
UK2 KJ437447     GTAGCCGCTCAAAAACCGAAACAATTTAAAGATTGGGTAAAATTAGCTACTGTAGATTTTAGTAATAATTGTAGAGGTAGTAACCAGGTATTTGTATTTT
Korea1 JX875304  GTGGCTGCTCAAAAACCGAAACAATTTAAAGATTGGGTAAAATTAGCTACTGTAGATTTTAGTAATACTTGTAGAGGTAGTAACCAGGTATTTGTATTTT
Korea2 JX878305  GTGGCTGCTCAAAAACCGAAACAATTTAAAGATTGGGTAAAATTAGCTACTGTAGATTTTAGTAATAATTGTAGAGGTAGTAACCAGGTATTTGTATTTT
China1 MF770715  GTGGCTGCTCAAAAACCGAAACAATTTAAAGATTGGGTAAAATTGGCTACTGTAGATTTTAGTAATAATTGTAGAGGTAGTAACCAGGTATTCGTATTTT
China2 MF036686  GTGGCTGCTCAAAAACCGAAACAATTTAAAGATTGGGTAAAATTAGCTACTGTAGATTTTAGTAATAATTGTAGAGGTAGTAACCAGGTATTCGTATTTT
China3 MH165180  GTGGCTGCTCAAAAACCGAAACAATTTAAAGATTGGGTAAAATTGGCTACTGTAGATTTTAGTAATAATTGTAGAGGTAGTAACCAGGTATTCGTATTTT
DWV-NVN          GTGGCTGCTCAAAAACCGAAACAATTTAAAGATTGGGTAAAATTAGCTACTGTAGATTTTAGTAATAATTGTAGAGGTAGCAATCAGGTATTTGTATTTT
DWV-SVN          GTGGCTGCTCAAAAACCGAAACAATTTAAAGATTGGGTAAAATTAGCTACTGTAGATTTTAGTAATAGTTGTAGAGGTAGCAATCAGGTATTTGTATTTT
```

```
                           5210      5220      5230      5240      5250      5260      5270      5280      5290      5300
                      ....|....|....|....|....|....|....|....|....|....|....|....|....|....|....|....|....|....|....|....|
Japan AB070959        TCAAGAATACATTTGAAGTGTTGAAGAAAATGTGGGGTTATGTATTTTGTCAGAGTAATCCTGCAGCGCGTTTGTTGAAAGCTGTGAATGACGAGCCTGA
USA1 AY291384         TCAAGAATACATTTGAAGTGTTGAAGAAAATGTGGGGTTATGTATTTTGTCAGAGTAATCCTGCAGCGCGTTTGTTGAAAGCTGTGAATGACGAGCCTGA
Chile JQ413340        TCAAGAATACATTTGAAGTGTTGAAGAAAATGTGGGGTTATGTTTTTTGTCAGAGTAATCCTGCAGCGCGTTTGTTGAAAGCTGTGAATGACGAGCCTGA
Italy1 AJ489744       TCAAGAATACATTTGAAGTGTTGAAGAAAATGTGGGGTTATGTATTTTGTCAGAGTAATCCTGCAGCGCGTTTGTTGAAAGCTGTGAATGACGAGCCTGA
France KX373899       TCAAGAATACATTTGAAGTGTTGAAGAAAATGTGGGGTTATGTATTTTGTCAGAGTAATCCTGCAGCGCGTTTGTTGAAAGCTGTGAATGACGAGCCTGA
UK1 GU109335          TCAAGAATACATTTGAAGTGTTGAAGAAAATGTGGGGTTATGTATTTTGTCAGAGTAATCCTGCAGCGCGTTTGTTGAAAGCTGTGAATGACGAGCCTGA
UK2 KJ437447          TCAAGAATACATTTGAAGTGTTGAAGAAAATGTGGGGTTATGTGTTTTGTCAGAGTAATCCTGCAGCGCGTTTGTTGAAAGCTGTGAATGACGAGCCTGA
Korea1 JX875304       TCAAAAATACATTTGAAGTGTTGAAGAAAATGTGGGGTTATGTATTTTGTCAGAGTAATCCTGCAGCGCGTTTGTTGAAAGCTGTGAATGACGAGCCTGA
Korea2 JX878305       TTAAGAATACATTTGAAGTGTTGAAGAAAATGTGGGGTTATGTGTTTTGTCAGAGTAATCCTGCAGCGCGTTTGTTGAAAGCTGTGAATGATGAGCCTGA
China1 MF770715       TCAAGAATACATTTGAAGTCTTGAAGAAAATGTGGGGTTATATATTTTGTCAGAGTAATCCTGCAGCGCGTTTGTTGAAAGCTGTGAATGACGAGCCTGA
China2 MF036686       TCAAGAATACATTTGAAGTCTTGAAGAAAATGTGGGGTTATATATTTTGTCAGAGTAATCCTGCAGCGCGTTTGTTGAAAGCTGTGAATGACGAGCCTGA
China3 MH165180       TTAAGAATACATTTGAAGTCTTGAAGAAAATGTGGGGTTATATATTTTGTCAGAGTAATCCTGCAGCGCGTTTGTTGAAAGCTGTGAATGACGAGCCTGA
DWV-NVN               TCAAGAATACGTTTGAAGTGTTGAAGAAAATGTGGGGTTACGTATTTTGTCAAAGTAATCCTGCAGCGCGTTTGTTGAAAGCTGTGAATGATGAGCCTGA
DWV-SVN               TCAAGAATACGTTTGAAGTGTTGAAGAAAATGTGGGGTTACGTATTCTGTCAAAGTAATCCTGCAGCGCGTTTGTTGAAAGCTGTGAATGATGAGCCTGA

                           5310      5320      5330      5340      5350      5360      5370      5380      5390      5400
                      ....|....|....|....|....|....|....|....|....|....|....|....|....|....|....|....|....|....|....|....|
Japan AB070959        GATTTTGAAAGCATGGGTGAAGGAATGTCTGTATTTGGATGATCCCAAATTTAGAATGCGTCGAGCGCATGATCAAGAGTATATCGAGAGAGTGTTTGCG
USA1 AY291384         GATTTTGAAAGCATGGGTGAAGGAATGTCTGTATTTGGATGATCCCAAATTTAGAATGCGTCGAGCGCATGATCAAGAGTATATCGAGAGAGTGTTTGCG
Chile JQ413340        AATTTTGAAAGCATGGGTAAAGGAATGTCTGTATTTGGATGATCCCAAATTCAGAATGCGTCGAGCGCATGATCAAGAGTATATCGAGAGAGTGTTTGCG
Italy1 AJ489744       GATTTTGAAAGCATGGGTGAAGGAATGTCTGTATTTGGATGATCCCAAATTCAGAATGCGTCGAGCGCATGATCAAGAGTATATCGAGAGAGTGTTTGCG
France KX373899       GATTTTGAAAGCATGGGTGAAGGAATGTCTGTATTTGGATGATCCCAAATTCAGAATGCGTCGAGCGCATGATCAAGAGTATATCGAGAGAGTGTTTGCG
UK1 GU109335          GATTTTGAAAGCATGGGTGAAGGAATGTCTGTATTTGGATGATCCTAAATTCAGAATGCGTCGAGCGCATGATCAAGAGTATATCGAGAGAGTGTTTGCG
UK2 KJ437447          GATTTTGAAAGCATGGGTGAAGGAATGTCTGTATTTGGATGATCCCAAATTCAGAATGCGTCGAGCGCATGATCAAGAGTACATCGAGAGAGTGTTTGCG
Korea1 JX875304       GATTTTGAAAGCATGGGTGAAGGAATGTCTGTATTTGGACGATCCCAAATTCAGAATGCGTCGAGCGCATGATCAAGAGTATATTGAGAGAGTGTTTGCG
Korea2 JX878305       GATTTTGAAAGCATGGGTGAAGGAATGTCTGTATTTGGACGATCCCAAATTCAGAATGCGTCGAGCGCATGATCAAGAGTATATCGAGAGAGTGTTTGCG
China1 MF770715       AATTTTGAAAGCATGGGTGAAGGAATGTCTGTATTTGGACGATCCCAAATTCAGAATGCGTCGAGCGCATGATCAAGAGTATATCGAGAGAGTGTTTGCG
China2 MF036686       GATTTTGAAAGCATGGGTGAAGGAATGTCTGTATTTGGACGATCCCAAATTCAGAATGCGTCGAGCGCATGATCAAGAGTATATCGAGAGAGTGTTTGCG
China3 MH165180       GATTTTGAAAGCATGGGTGAAGGAATGTCTGTATTTGGACGATCCCAAGTTCAGAATGCGTCGAGCGCATGATCAAGAGTATATCGAGAGAGTGTTTGCG
DWV-NVN               GATTTTGAAAGCATGGGTGAAAGAATGTCTGTATTTGGATGATCCCAAATTTAGAATGCGTCGAGCGCATGATCAAGAATATATTGAGAGAGTGTTTGCG
DWV-SVN               GATTTTGAAAGCATGGGTGAAAGAATGTCTGTATTTGGATGATCCCAAATTTAGAATGCGTCGAGCGCATGATCAAGAATATATTGAGAGAGTGTTTGCG

                           5410      5420      5430      5440      5450      5460      5470      5480      5490      5500
                      ....|....|....|....|....|....|....|....|....|....|....|....|....|....|....|....|....|....|....|....|
Japan AB070959        GCACATTCATATGGACAAATTTTGCTACATGATTTAACTGCTGAAATGAATCAATCACGAAATTTGAGTGTGTTTACACGTGTGTATGATCAAATTTCAA
USA1 AY291384         GCACATTCATATGGACAAATTTTGCTACATGATTTAACTGCTGAAATGAATCAATCACGAAATTTGAGTGTGTTTACACGTGTGTATGATCAAATTTCAA
Chile JQ413340        GCACATTCATATGGACAAATTTTGCTACATGATTTAACTGCTGAAATGAATCAATCACGAAATTTGAGTGTGTTTACACGTGTGTATGATCAAATTTCAA
Italy1 AJ489744       GCACATTCATATGGACAAATTTTGCTACATGATTTAACTGCTGAAATGAATCAATCACGAAATTTGAGTGTGTTTACACGTGTGTATGATCAAATTTCAA
France KX373899       GCACATTCTTATGGACAAATTTTGCTACATGATTTAACTGCTGAAATGAATCAATCACGAAATTTGAGTGTGTTTACACGTGTGTATGATCAAATTTCAA
UK1 GU109335          GCACATTCATATGGACAAATATTGCTACATGATTTAACTGCTGAAATGAATCAATCACGAAATTTGAGTGTGTTTACACGTGTGTATGATCAAATTTCAA
UK2 KJ437447          GCACATTCTTATGGACAAATTTTGCTACATGATTTAACTGCTGAAATGAATCAATCACGAAATTTGAGTGTGTTTACACGTGTGTATGATCAAATTTCAA
Korea1 JX875304       GCACATTCATATGGACAAATTTTGCTACATGATTTAACTGCTGAAATGAATCAATCACGAAATTTGAGTGTGTTTACACGTGTGTATGATCAAATTTCAA
Korea2 JX878305       GCACATTCATATGGACAAATTTTGCTACATGATTTAACTGCTGAAATGAATCAATCACGAAATTTGACTGTGTTTACACGTGTGTATGATCAAATTTCAA
China1 MF770715       GCACATTCATATGGACAAATTTTGCTACATGATTTAACTGCTGAAATGAATCAATCACGAAATTTGAGTGTGTTTACACGTGTGTATGATCAGATTTCAA
China2 MF036686       GCACATTCATATGGACAAATTTTGCTACATGATTTAACTGCTGAAATGAATCAATCACGAAATTTGAGTGTGTTTACACGTGTGTATGATCAAATTTCAA
China3 MH165180       GCACATTCATATGGACAAATTTTGCTCCATGATTTAACTGCTGAAATGAATCAATCACGAAATTTGAGCGTGTTTACACGTGTGTATGATCAGATTTCAA
DWV-NVN               GCGCATTCGTATGGGCAGATTTTGCTACACGATTTGACTGCTGAAATGAATCAATCACGGAATTTGAGTGTGTTCACACGTGTGTATGATCAAATTTCAA
DWV-SVN               GCGCATTCGTATGGGCAGATTTTGCTACACGATTTGACTGCTGAAATGAATCAATCACGGAATTTGAGTGTGTTCACACGTGTGTATGATCAAATTTCAA

                           5510      5520      5530      5540      5550      5560      5570      5580      5590      5600
                      ....|....|....|....|....|....|....|....|....|....|....|....|....|....|....|....|....|....|....|....|
Japan AB070959        AATTGAAGACTGATCTTATGGAAATGGGATCGAATCCATATATAAGGCGTGAATGTTTTACGATATGCATGTGTGGTGCATCTGGAATTGGAAAATCATA
USA1 AY291384         AATTGAAGACTGATCTTATGGAAATGGGATCGAATCCATATATAAGGCGTGAATGTTTTACGATATGCATGTGTGGTGCATCTGGAATTGGAAAATCATA
Chile JQ413340        AATTGAAAACCGATCTTATGGAAATGGGATCGAATCCATATATAAGGCGAGAATGTTTTACGATATGCATGTGTGGTGCATCTGGAATTGGAAAATCATA
Italy1 AJ489744       AATTGAAGACCGATCTTATGGAAATGGGATCGAATCCATATATAAGGCGTGAATGTTTTACGATATGCATGTGTGGTGCATCTGGAATTGGAAAATCATA
France KX373899       AATTGAAGACCGATCTTATGGAAATGGGATCGAATCCATATATAAGGCGTGAATGTTTTACGATATGCATGTGTGGTGCATCTGGAATTGGAAAATCATA
UK1 GU109335          AATTGAAGACCGATCTTATGGAAATGGGATCGAATCCATATATAAGGCGTGAATGTTTTACGATATGCATGTGTGGTGCATCTGGAATTGGAAAATCATA
UK2 KJ437447          AATTGAAGACCGATCTTATGGAAATGGGATCGAATCCATATATAAGGCGTGAATGTTTTACGATATGTATGTGTGGTGCATCTGGAATTGGAAAATCATA
Korea1 JX875304       AATTGAAGACTGATCTCATGGAAATGGGATCGAATCCTTATATAAGGCGTGATTGTTTTACCATATGCATGTGTGGTGCATCTGGAATTGGAAAATCATA
Korea2 JX878305       AATTGAAGACTGATCTCATGGAAATGGGATCGAATCCATATATAAGGCGTGAATGTTTTACGATATGCATGTGTGGTGCATCTGGAATTGGAAAATCATA
China1 MF770715       AATTGAAAACCGATCTTATGGAAATGGGATCGAATCCATATATAAGGCGTGAATGTTTTACGATATGCATGTGTGGTGCATCTGGAATTGGAAAATCATA
China2 MF036686       AATTGAAGACTGATCTTATGGAAATGGGATCGAATCCATATATAAGGCGTGAATGTTTTACGATTTGCATGTGTGGTGCATCTGGAATTGGAAAATCATA
China3 MH165180       AATTGAAAACTGATCCTATGGAAATGGGATCGAATCCATATATAAGGCGTGAATGTTTTACGATATGCATGTGTGGTGCATCTGGAATTGGTAAATCATA
DWV-NVN               AATTGAAAACTGATCTCATGGAAATGGGATCGAATCCATACATAAGGCGTGAGTGTTTTACGATATGCATGTGTGGTGCATCTGGAATTGGAAAATCATA
DWV-SVN               AATTGAAAACTGATCTCATGGAAATGGGATCAAATCCATACATAAGGCGTGAGTGTTTTACGATATGCATGTGTGGTGCATCTGGAATTGGAAAATCATA
```

```
                           5610      5620      5630      5640      5650      5660      5670      5680      5690      5700
                   ....|....|....|....|....|....|....|....|....|....|....|....|....|....|....|....|....|....|....|....|
Japan AB070959     TTTGACCGATTCTTTATGCAGCGAGCTCTTACGTGCGAGTCGTACTCCTGTGACAACAGGCATAAAATGTGTTGTTAATCCATTATCTGATTATTGGGAT
USA1 AY291384      TTTGACCGATTCTTTATGCAGCGAGCTCTTACGTGCGAGTCGTACTCCTGTGACAACAGGCATAAAATGTGTTGTTAATCCATTATCTGATTATTGGGAT
Chile JQ413340     TTTGACCGATTCTTTATGCAGCGAGCTCTTACGTGCGAGTCGTACTCCTGTGACAACAGGCATAAAATGTGTTGTTAATCCATTATCTGATTATTGGGAT
Italy1 AJ489744    TTTGACCGATTCTTTATGCAGCGAGCTCTTACGTGCGAGTCGTACTCCTGTGACAACAGGCATAAAATGTGTTGTTAATCCATTATCTGATTATTGGGAT
France KX373899    TTTGACCGATTCTTTATGCAGCGAGCTCTTACGTGCGAGTCGTACTCCTGTGACAACAGGCATAAAATGTGTTGTTAATCCATTATCTGATTATTGGGAT
UK1 GU109335       TTTGACCGATTCTTTATGCAGCGAGCTCTTACGTGCGAGTCGTTCTCCTGTGACAACAGGCATAAAATGTGTTGTTAATCCATTATCTGATTATTGGGAT
UK2 KJ437447       TTTGACCGATTCTTTATGCAGCGAGCTCTTACGTGCGAGTCGTACTCCTGTGACAACAGGCATAAAATGTGTTGTTAATCCATTATCTGATTATTGGGAT
Korea1 JX875304    TTTTACCGATTCCTTATGCAGGGAGCTCTTACGTGCGAGTCGTACTCCTGTGACAACGGGCATAAAATGTGTTGTTAATCCATTATCCGATTATTGGGAT
Korea2 JX878305    TTTGACCGATTCTTTATGCAGCGAGCTCTTACGTGCGAGTCGTACTCCTGTGACAACAGGCATAAAATGTGTTGTTAATCCATTATCTGATTATTGGGAT
China1 MF770715    TTTGACCGATTCTTTATGCAGCGAGCTCTTACGTGCGAGTCGAACTCCTGTGACTACAGGCATAAAATGTGTTGTTAATCCATTATCTGATTATTGGGAT
China2 MF036686    TTTGACCGATTCTTTATGCAGCGAGCTCTTACGTGCGAGTCGTACTCCTGTGACAACAGGCATAAAATGTGTTGTTAATCCATTATCTGATTATTGGGAT
China3 MH165180    TTTGACCGATTCTTTATGCAGCGAGCTCTTACGTGCGAGTCGTACTCCTGTGACTACAGGCATAAAATGTGTTGTTAATCCATTATCTGATTATTGGGAT
DWV-NVN            TTTAACCGATTCCTTATGCAGCGAGCTCTTACGTGCGAGTCGAACTCCTGTGACGACGGGCATAAAGTGTGTTGTGAATCCATTATCTGATTATTGGGAT
DWV-SVN            TTTAACCGATTCCTTATGCAGCGAGCTCTTACGTGCGAGTCGAACTCCTGTGACGACGGGCATAAAGTGTGTTGTGAATCCATTATCTGATTATTGGGAT

                           5710      5720      5730      5740      5750      5760      5770      5780      5790      5800
                   ....|....|....|....|....|....|....|....|....|....|....|....|....|....|....|....|....|....|....|
Japan AB070959     CARTGTGATTTTCAGCCTGTTTTGTGCGTTGACGATATGTGGAGTGTTGAAACATCTACTACGCTYGATAAGCAGTTGAAYATGCTTTTYCAGGTCCATT
USA1 AY291384      CAGTGTGATTTTCAGCCTGTTTTGTGCGTTGACGATATGTGGAGTGTTGAAACATCTACTACGCTCGATAAGCAGTTGAACATGCTTTTCCAGGTCCATT
Chile JQ413340     CAATGTGATTTTCAACCTGTTTTGTGCGTTGATGATATGTGGAGTGTTGAAACATCTACTACGCTCGATAAGCAGTTGAATATGCTTTTCCAGGTCCATT
Italy1 AJ489744    CAATGTGATTTTCAGCCTGTTTTGTGCGTTGACGATATGTGGAGTGTTGAAACATCTACTACGCTYGATAAGCAGTTGAATATGCTTTTCCAGGTCCATT
France KX373899    CAATGTGATTTTCAGCCTGTTTTGTGCGTTGACGACATGTGGAGTGTTGAAACATCTACTACGCTCGATAAGCAGTTGAATATGCTTTTCCAGGTCCATT
UK1 GU109335       CAATGTGATTTTCAGCCTGTTTTGTGCGTTGATGATATGTGGAGTGTTGAAACATCTACTACGCTCGATAAGCAGTTGAATATGCTTTTCCAGGTCCATT
UK2 KJ437447       CAATGTGATTTTCAGCCTGTTTTGTGCGTTGACGATATGTGGAGTGTTGAAACATCTACTACGCTCGATAAGCAGTTGAATATGCTTTTCCAGGTCCATT
Korea1 JX875304    CAATGTGATTTTCAGCCTGTTTTGTGGGTTGATGATATGTGGAGTGTTGAAACATCTACTACACTCGATAAGCAGTTGAATATGCTGTTCCAGGTCCATT
Korea2 JX878305    CAATGTGATTTTCAGCCTGTTTTGTGCGTTGACGATATGTGGAGTGTTGAAACATCTACTACGCTCGATAAGCAGTTGAATATGCTTTTCCACGTCCATT
China1 MF770715    CAATGTGATTTTCAGCCTGTTTTGTGCGTTGATGATATGTGGAGTGTTGAAACATCTACTACGCTCGATAAGCAGTTGAATATGCTTTTCCAGGTTCATT
China2 MF036686    CAATGTGATTTTCAGCCTGTTTTGTGCGTTGACGATATGTGGAGTGTTGAAACATCTACCACGCTCGATAAGCAGTTGAATATGCTTTTCCAGGTACATT
China3 MH165180    CAATGTGATTTTCAGCCTGTTTTGTGCGTTGACGATATGTGGAGTGTTGAAACATCTACTACGCTCGATAAGCAGTTGAATATGCTTTTCCAGGTTCATT
DWV-NVN            CAGTGTGATTTTCAACCTGTTTTATGTGTTGACGACATGTGGAGCGTTGAAACATCTACTACGCTTGATAAGCAGTTGAACATGCTTTTCCAGGTTCATT
DWV-SVN            CAGTGTGATTTTCAGCCTGTTTTATGTGTTGACGACATGTGGAGTGTTGAAACATCTACTACGCTTGATAAGCAGTTGAATATGCTTTTCCAGGTTCATT

                           5810      5820      5830      5840      5850      5860      5870      5880      5890      5900
                   ....|....|....|....|....|....|....|....|....|....|....|....|....|....|....|....|....|....|....|
Japan AB070959     CCCCTATCGTGCTTTCTCCTCCTAAAGCTGATTTAGAAGGCAAGAAAATGCGATATAACCCGGAAATATTCATATACAATACGAATAAACCTTTCCCGAG
USA1 AY291384      CCCCTATCGTGCTTTCTCCTCCTAAAGCTGATTTAGAAGGTAAGAAAATGCGATATAACCCGGAAATATTCATATACAATACGAATAAGCCTTTCCCGAG
Chile JQ413340     CTCCTATCGTGCTTTCTCCTCCTAAAGCTGATTTAGAAGGTAAGAAAATGCGATATAACCCGGAAATATTCATATACAATACGAATAAACCTTTCCCGAG
Italy1 AJ489744    CCCCTATCGTGCTTTCTCCTCCTAAAGCTGATTTAGAAGGTAAGAAAATGCGATATAACCCGGAAATATTCATATACAATACGAATAAACCTTTCCCGAG
France KX373899    CCCCTATCGTGCTTTCTCCTCCTAAAGCTGATTTAGAAGGTAAGAAAATGCGCTATAACCCGGAAATATTCATATACAATACGAATAAACCTTTCCCGAG
UK1 GU109335       CCCCTATCGTGCTTTCCCCTCCTAAAGCTGATTTAGAAGGTAAGAAAATGCGATATAACCCGGAAATATTCATATACAATACGAATAAACCTTTCCCGAG
UK2 KJ437447       CCCCTATCGTGCTTTCCCCTCCTAAAGCTGATTTAGAAGGTAAGAAAATGCGATATAACCCGGAAATATTCATATACAATACGAATAAACCTTTCCCGAG
Korea1 JX875304    CCCCTATCGTGCTTTCTCCTCCTAAGGCTGATTTAGAAGGTAAGAAAATGCGATATAACCCGGAAATATTCATATACAATACGAATAAACCTTTCCCGAG
Korea2 JX878305    CCCCTATCGTGCTTTCTCCTCCTAATGCTGATTTGGAGGGTAAGAAAATGCGATATAACCCGGAAATATTCATATACAATACGAATAAACCTTTCCCGAG
China1 MF770715    CCCCTATCGTGCTCTCGCCTCCTAAAGCTGATTTGGAAGGTAAGAAAATGCGATATAACCCGGAAATATTCATATACAATACGAATAAACCTTTCCCGAG
China2 MF036686    CTCCTATCGTGCTTTCTCCTCCTAAAGCTGATTTAGAAGGTAAGAAAATGCGATATAACCCGGAAATATTCATATACAATACGAATAAACCTTTCCCGAG
China3 MH165180    CCCCTATCGTGCTTTCGCCTCCTAAAGCTGATTTGGAAGGTAAGAAAATGCGATATAACCCGGAAATATTCATATACAATACGAATAAACCTTTCCCGAG
DWV-NVN            CTCCTATCGTGCTTTCTCCTCCTAAGGCTGACTTAGAAGGCAAGAAAATGCGGTATAATCCGGAGATATTTATATACAATACGAACAAGCCTTTCCCGAG
DWV-SVN            CTCCTATCGTACTTTCTCCCCCTAAGGCTGACTTAGAAGGCAAGAAAATGCGTTATAATCCGGAGATATTTATATACAATACGAACAAGCCTTTTCCGAG

                           5910      5920      5930      5940      5950      5960      5970      5980      5990      6000
                   ....|....|....|....|....|....|....|....|....|....|....|....|....|....|....|....|....|....|....|
Japan AB070959     GTTTGATCGTATTGCTATGGAAGCTATTTATCGGCGTAGAAATGTTTTGATTGAATGCAAAGCGAGTGAAGAGAAGAAGCGAGGGTGCAAGCATTGTGAG
USA1 AY291384      GTTTGATCGTATTGCTATGGAAGCTATTTATCGGCGTAGAAATGTTTTGATTGAATGTAAAGCGAGTGAAGAGAAGAAGCGAGGATGCAAGCATTGTGAG
Chile JQ413340     GTTTGATCGGATTGCTATGGAAGCTATTTATCGGCGTAGAAATGTTTTGATTGAATGTAAAGCGAGTGAAGAGAAGAAGCGAGGATGTAAGCATTGTGAG
Italy1 AJ489744    GTTTGATCGTATTGCTATGGAAGCTATTTATCGGCGTAGAAATGTTTTGATTGAATGTAAAGCGAGTGAAGAGAAGAAGCGAGGATGTAAGCATTGTGAG
France KX373899    GTTTGATCGTATTGCTATGGAAGCTATTTATCGGCGTAGAAATGTTTTGATTGAATGTAAAGCGAGTGAAGAGAAGAAGCGAGGATGTAAGCATTGTGAG
UK1 GU109335       GTTTGATCGTATTGCTATGGAAGCTATTTATCGGCGTAGAAATGTTTTGATTGAATGTAAAGCGAGTGAAGAGAAGAAGCGAGGGTGTAAGCATTGTGAG
UK2 KJ437447       GTTTGATCGTATTGCTATGGAAGCTATTTATCGGCGTAGAAATGTTTTGATTGAATGTAAAGCGAGTGAAGAGAAGAAGCGAGGATGTAAGCATTGTGAG
Korea1 JX875304    GTTTGATCGTATCGCTATGGAAGCTATTTATCGGCGTAGAAATGTTTTGATTGAATGTAAAGCGAATGAGGAGAAGAAGCGAGGATGCAAGCATTGTGAG
Korea2 JX878305    GTTTGATCGTATTGCTATGGAAGCCATTTATCGGCGTAGAAATGTTTTGATTGAATGTAAAGCGAGTGAAGAGAAGAAGCGAGGATGCAAGCATTGTGAG
China1 MF770715    GTTTGATCGCATTGCTATGGAAGCTATTTATCGGCGTAGAAATGTTTTGATTGAATGTAGAGCGAGTGAAGAGAAGAAGCGAGGATGCAAGCATTGTGAG
China2 MF036686    GTTTGATCGTATTGCTATGGAAGCTATTTATCGGCGTAGAAATGTTTTGATTGAATGTAAAGCGAGTGAAGAGAAGAAGCGAGGATGCAAGCATTGTGAG
China3 MH165180    GTTTGATCGTATTGCTATGGAAGCTATTTATCGGCGTAGAAATGTTTTGATTGATTGTAAAGCGAGTGAAGAGAAGAAGCGAGGATGCAAGCATTGTGAG
DWV-NVN            GTTTGATCGTATTGCCATGGAAGCTATTTATCGGCGTAGAAATGTTTTGATTGAATGTAAGGCGAGTGAAGAGAAGAAGCGAGGATGTAAGCATTGTGAG
DWV-SVN            GTTTGATCGTATTGCCATGGAAGCTATTTATCGGCGTAGAAATGTTTTGATTGAATGTAAGGCGAGTGAAGAGAAGAAGCGAGGATGTAAGCATTGTGAG
```

```
                        6010      6020      6030      6040      6050      6060      6070      6080      6090      6100
                    ....|....|....|....|....|....|....|....|....|....|....|....|....|....|....|....|....|....|....|....|
Japan AB070959      AATGATATTCCTATTGCTGAATGTAGTCCTAAGATGTTGAAAGATTTTCATCATATTAAGTTTAGGTATGCACATGATGTATGTAATTCCGAGACCACAT
USA1 AY291384       AATGATATTCCTATTGCTGAATGTAGTCCTAAGATGTTGAAAGATTTCCATCATATCAAATTTAGGTATGCACATGATGTATGTAATTCCGAGACCACAT
Chile JQ413340      AATGATATTCCTATTGCTGAATGTAGTCCTAAGATGTTGAAAGATTTTCATCATATTAAATTTAGGTATGCACATGATGTATGTAATTCCGAGACCACAT
Italy1 AJ489744     AATGATATTCCTATTGCTGAATGTAGTCCTAAGATGTTGAAAGATTTTCATCATATTAAATTTAGGTATGCGCATGATGTATGTAATTCCGAGACCACAT
France KX373899     AATGATATTCCTATTGCTGAATGTAGTCCTAAGATGTTGAAAGATTTTCATCATATTAAATTTAGGTATGCACATGATGTATGTAATTCCGAGACTACTT
UK1 GU109335        AATGATATTCCTATTGCTGAATGCAGTCCTAAGATGTTGAAAGATTTTCATCATATTAAATTTAGGTATGCACATGACGTATGTAATTCCGAGACCACAT
UK2 KJ437447        AATGATATCCCTATTGCTGAATGTAGTCCTAAAATGTTGAAAGATTTTCATCATATTAAATTTAGGTATGCACATGATGTGTGTAATTCCGAAACCACAT
Korea1 JX875304     AATGATATTCCTATTGCTGAATGTAGTCCTAAGATGTTGAAAGATTTCCATCATATTAAATTTAGGTATGCACATGATGTATGTAATTCCGAGACCACAT
Korea2 JX878305     AATGATATTCCTATTGCTGAATGTAGTCCTAAGATGCTGAAAGATTTTCACCATATCAAATTTAGGTATGCACATGATGTATGTAATTCCGAGACCACAT
China1 MF770715     AATGATATTCCTATTGCTGAGTGTAGTCCTAAGATGTTGAAAGATTTTCATCATATTAAATTTCGGTATGCACACGATGTATGTAATTCCGAGACCACAT
China2 MF036686     AATGATATTCCTATTGCTGAATGTAGTCCTAAGATGTTGAAAGATTTTCATCATATTAAATTTAGGTATGCACATGATGTATGTAATTCCGAGACCACAT
China3 MH165180     AATGATATTCCTATTGCTGAATGTAGCCCTAAGATGTTGAAAGATTTTCATCATATTAAATTTCGGTATGCACACGATGTATGTAATTCCGAGACCACAT
DWV-NVN             GATGATATTCCTATTGCTGAGTGTAGTCCCAAAATGTTGAAAGATTTTCACCACATTAAATTTAGGTATGCACATGATGTATGCAATTCTGAGACTACGT
DWV-SVN             AATGATATTCCCATTGCTGAGTGTAGTCCCAAAATGTTGAAAGATTTTCACCACATTAAATTCAGGTATGCACATGATGTATGCAATTCTGAGACTACGT

                        6110      6120      6130      6140      6150      6160      6170      6180      6190      6200
                    ....|....|....|....|....|....|....|....|....|....|....|....|....|....|....|....|....|....|....|....|
Japan AB070959      GGTCTGAATGGATGACGTATAATGAATTTCTTGAATGGATAACTCCTGTGTATATGGCTAATCGTCGTAAGGCGAATGAATCATTTAAGATGCGTGTGGA
USA1 AY291384       GGTCTGAATGGATGACGTATAATGAATTTCTTGAATGGATAACTCCTGTGTATATGGCTAACCGTCGTAAGGCGAATGAATCGTTTAAGATGCGTGTAGA
Chile JQ413340      GGTCTGAATGGATGACGTATAATGAATTTCTTGAATGGATAACTCCTGTGTATATGGCTAATCGTCGTAAGGCGAATGAATCGTTTAAGATGCGTGTGGA
Italy1 AJ489744     GGTCTGAATGGATGACGTATAATGAATTTCTTGAATGGATAACTCCTGTGTATATGGCTAACCGTCGTAAGGCGAATGAATCGTTTAAGATGCGTGTGGA
France KX373899     GGTCTGAATGGATGACGTATAATGAATTTCTTGAATGGATAACTCCTGTGTATATGGCTAACCGTCGTAAGGCGAATGAATCGTTTAAGATGCGTGTGGA
UK1 GU109335        GGTCTGAATGGATGACGTATAATGAATTTCTTGAATGGATAACTCCTGTGTATATGGCTAACCGTCGTAAGGCGAATGAATCGTTTAAGATGCGTGTGGA
UK2 KJ437447        GGTCTGAATGGATGACGTATAATGAATTTCTTGAATGGATAACTCCTGTGTATATGGCTAACCGTCGTAAGGCGAATGAATCGTTTAAGATGCGTGTGGA
Korea1 JX875304     GGTCTGAATGGATGACGTATAATGAATTCCTTGAAAGGATAACTCCTGTGTATATGGCTAACCGTCGTAAGGCGAATGAATCGTTTAGAATGCGTGTGGA
Korea2 JX878305     GGTCTGAATGGATGACGTATAATGAATTTCTTGAATGGATAACACCTGTGTATATGGCTAACCGTCGTAAGGCGAATGAATCGTTTAAGATGCGTGTGGA
China1 MF770715     GGTCTGAATGGATGACGTATAATGAATTTCTTGAATGGATAACTCCGGTGTATATGGCTAATCGTCGTAAGGCGAATGAGTCGTTTAAGATGCGTGTGGA
China2 MF036686     GGTCTGAATGGATGACATATAATGAATTTCTTGAATGGATAACTCCTGTGTATATGGCTAACCGTCGTAAGGCGAATGAATCGTTTAAGATGCGTGTGGA
China3 MH165180     GGTCTGAATGGATGACGTATAATGAATTTCTTGAATGGATAACTCCGGTGTATATGGCTAATCGTCGTAAGGCGAATGAATCGTTTAAGATGCGTGTGGA
DWV-NVN             GGTCTGAGTGGATGACATATAATGAATTTCTTGAATGGATAACTCCTGTATATATGGCTAATCGACGTAAGGCGAATGAATCGTTTAAAATGCGTGTGGA
DWV-SVN             GGTCTGAGTGGATGACATATAATGAATTTCTTGAATGGATAACTCCTGTATATATGGCTAATCGACGTAAGGCGAATGAATCGTTTAAAATGCGTGTGGA

                        6210      6220      6230      6240      6250      6260      6270      6280      6290      6300
                    ....|....|....|....|....|....|....|....|....|....|....|....|....|....|....|....|....|....|....|....|
Japan AB070959      TGAAATGCAAATGTTGCGTATGGATGAACCATTGGAAGGTGATAATATTCTCAATAAGTACGTTGAAGTCAATCAGCGCTTAGTGGAGGAAATGAAAGCA
USA1 AY291384       TGAAATGCAAATGTTACGTATGGATGAACCATTGGAAGGTGATAACATTCTCAATAAGTATGTTGAAGTTAATCAGCGCTTAGTGGAGGAAATGAAGGCA
Chile JQ413340      TGAAATGCAAATGTTACGTATGGATGAACCATTGGAAGGTGATAATATTCTCAATAAGTATGTTGAAGTTAATCAGCGTTTAGTGGAGGAAATGAAGGCA
Italy1 AJ489744     TGAAATGCAAATGTTACGTATGGATGAACCATTGGAAGGTGATAATATTCTCAATAAGTATGTTGAAGTTAATCAGCGCTTAGTGGAGGAAATGAAGGCA
France KX373899     CGAAATGCAAATGTTACGTATGGATGAACCATTGGAAGGTGATAATATCCTCAATAAGTATGTTGAAGTTAATCAGCGTTTAGTGGAGGAAATGAAGGCA
UK1 GU109335        TGAAATGCAAATGTTACGTATGGATGAACCATTGGAAGGTGATAATATTCTCAATAAGTATGTTGAAGTTAATCAGCGCTTAGTGGAGGAAATGAAGGCA
UK2 KJ437447        TGAAATGCAAATGCTACGTATGGATGAGCCATTGGAAGGTGATAATATCCTCAATAAGTATGTTGAAGTTAATCAGCGCTTAGTGGAGGAAATGAAGGCA
Korea1 JX875304     TGAAATGCAAATGTTACGTATGGATGAACCATTGGAAGGTGATAATATTCTCAATAAGTATGTTGAAGTTAATCAGCGCTTAGTGGAGGAAATGAAGGCG
Korea2 JX878305     TGAAATGCAAATGTTACGTATGGATGAACCATTGGAAGGTGATAATATTCTCAATAAGTATGTTGAAGTTAATCAGCGCTTAGTGGAGGAAATGAAGGCA
China1 MF770715     TGAAATGCAAATGTTACGTATGGATGAACCATTGGAAGGTGATAATATTCTCAATAAGTATGTTGAAGTTAATCAGCGCTTAGTGGAGGAAATGAAGGCA
China2 MF036686     TGAAATGCAAATGTTACGTATGGATGAACCATTGGAGGGTGATAATATTCTCAATAAGTATGTTGAAGTTAATCAGCGTTTAGTGGAGGAAATGAAGGCA
China3 MH165180     TGAAATGCAAATGTTACGTATGGATGAACCATTGGAAGGTGATAATATTCTTAATAAGTATGTTGAAGTTAATCAGCGCTTAGTGGAGGAAATGAAGGCA
DWV-NVN             TGAGATGCAAATGTTACGTATGGATGAACCATTGGAAGGTGATAATATCCTTAATAAGTATGTTGAAGTTAACCAGCGTTTAGTAGAGGAAATGAAGGCA
DWV-SVN             TGAGATGCAAATGTTACGTATGGATGAACCATTGGAAGGCGATAATATCCTTAATAAGTATGTTGAAGTTAACCAGCGTTTAGTAGAGGAAATGAAGGCA

                        6310      6320      6330      6340      6350      6360      6370      6380      6390      6400
                    ....|....|....|....|....|....|....|....|....|....|....|....|....|....|....|....|....|....|....|....|
Japan AB070959      TTTAAGGAGCGTACACTATGGTCAGATTTACATCGCGTAGGTGCGGAAATTAGTGCGTCAGTTAAGAAAGCTTTACCAACCATTTCCATAACTGAAAAAC
USA1 AY291384       TTTAAGGAGCGTACACTATGGTCAGATTTACATCGCTTAGGTGCGGAAATTAGTGCGTCAGTTAAGAAAGCTTTACCAACTATTTCCATAACCGAAAAAC
Chile JQ413340      TTTAAGGAGCGTACACTATGGTCAGATTTACATCGCGTAGGTGCGGAAATTAGTGCGTCAGTTAAGAAAGCTTTACCAACCATTTCCATAACTGAAAAAC
Italy1 AJ489744     TTTAAGGAGCGTACACTATGGTCAGATTTACATCGCGTAGGTGCGGAAATTAGTGCGTCAGTTAAGAAAGCTTTACCAACCATTTCCATAACCGAAAAAY
France KX373899     TTTAAGGAACGTACACTATGGTCAGATTTACATCGCGTAGGTGCGGAAATTAGTGCGTCAGTTAAGAAAGCTTTACCAACCATTTCCATAACCGAAAAAC
UK1 GU109335        TTTAAGGAGCGTACACTATGGTCAGATTTACATCGCGTAGGTGCGGAAATTAGTGCGTCAGTTAAGAAAGCTTTACCAACCATTTCCATAACCGAAAAAT
UK2 KJ437447        TTTAAGGAGCGTACACTATGGTCAGATTTACATCGCGTAGGTGCGGAAATTAGTGCGTCAGTTAAGAAAGCTTTACCAACCATTTCCATAACCGAAAAAT
Korea1 JX875304     TTTAAAGAGCGCACACTATGGTCAGATTTACATCGCGTAGGTGCGGAAATTAGTGCGTCAGTTAAGAAAGCTTTACCAACCATTTCCATAACTGAAAAAC
Korea2 JX878305     TTTAAGGATCGTACACTATGGTCAGATTTACATCGCGTAGGTGCGGAAATTAGTGCGTCAGTTAAGAAAGCTTTGCCGACTATTTCCATAACCGAAAAAC
China1 MF770715     TTTAAGGAACGTACACTATGGTCAGATTTACATCGCGTAGGTGCGGAAATTAGTGCGTCAGTTAAGAAAGCTTTACCAACCATTTCCATAACCGAAAAAT
China2 MF036686     TTTAAGGAGCGTACGCTATGGTCAGATTTACATCGTGTAGGTGCGGAAATTAGTGCGTCAGTTAAGAAAGCTTTACCAACCATTTCCATAACCGAAAAAT
China3 MH165180     TTTAAGGAGCGTACACTATGGTCAGATTTACATCGCGTAGGTGCGGAGATTAGTGCGTCAGTTAAGAAAGCTTTACCAACCATTTCCATAACTGAAAAAC
DWV-NVN             TTTAAGGAACGCACACTATGGTCAGACTTACATCGTGTAGGTGCGGAAATTAGTGCTTCAGTTAAGAAAGCTTTACCAACTATTTCTATAACTGAAAAAC
DWV-SVN             TTTAAGGAACGCACACTATGGTCAGATTTACATCGTGTAGGTGCGGAAATTAGTGCTTCAGTTAAGAAAGCTTTACCAACTATTTCTATAACGGAAAAAT
```

```
                            6410      6420      6430      6440      6450      6460      6470      6480      6490      6500
                    ....|....|....|....|....|....|....|....|....|....|....|....|....|....|....|....|....|....|....|....|
Japan AB070959      TACCACATTGGACTGTTCAATGTGGTATTGCTAAACCTGAGATGGATCATGCTTATGAGGTTATGAGTTCGTATGCAGCTGGAATGAATGCAGAGATTGA
USA1 AY291384       TACCACATTGGACTGTTCAATGTGGTATTGCTAAACCTGAGATGGATCATGCTTATGAGGTTATGAGTTCGTATGCAGCTGGAATGAATGCAGAGATTGA
Chile JQ413340      TACCGCATTGGACTGTTCAATGTGGTATTGCTAAACCTGAGATGGATCATGCTTATGAGGTTATGAGTTCATATGCAGCCGGAATGAATGCAGAGATTGA
Italy1 AJ489744     TACCACATTGGACTGTTCAATGTGGTATTGCTAAACCTGARATGGACCATGCTTATGAGGTTATGAGTTCGTATGCAGCTGGAATGAATGCAGAGATTGA
France KX373899     TACCACATTGGACTGTTCAATGTGGTATTGCTAAACCTGAGATGGACCATGCTTATGAGGTTATGAGTTCGTATGCAGCTGGAATGAATGCAGAGATTGA
UK1 GU109335        TACCACATTGGACTGTTCAATGTGGTATTGCTAAACCTGAGATGGACCATGCTTATGAGGTTATGAGTTCGTATGCAGCTGGAATGAATGCAGAGATTGA
UK2 KJ437447        TACCACATTGGACTGTTCAATGTGGTATTGCTAAACCTGAGATGGACCATGCTTATGAGGTTATGAGTTCGTATGCAGCTGGAATGAATGCAGAGATTGA
Korea1 JX875304     TACCACATTGGACTGTTCAATGTGGCATTGCTAAACCTGAAATGGATCATGCTTATGAGGTTATGAGTTCGTATGCAGCTGGAATGAATGCAGAGATTGA
Korea2 JX878305     TACCACATTGGACTGTTCAATGTGGTATTGCTAAACCTGAGATGGATCATGCCTATGAGGTTATGAGTTCGTATGCAGCCGGAATGAATGCAGAGATTGA
China1 MF770715     TACCACATTGGACTGTTCAGTGTGGTATTGCTAAACCTGAGATGGATCATGCTTATGAGGTTATGAGTTCGTATGCAGCTGGAATGAATGCAGAGATCGA
China2 MF036686     TACCACATTGGACTGTTCAATGTGGTATTGCTAAACCTGAGATGGATCATGCTTATGAGGTTATGAGTTCGTATGCAGCTGGAATGAATGCAGAGATTGA
China3 MH165180     TACCACATTGGACTGTTCAATGTGGTATCGCTAAACCTGAGATGGATCATGCTTATGAGGTTATGAGTTCGTATGCAGCTGGAATGAATGCAGAGATTGA
DWV-NVN             TACCACATTGGACTGTTCAATGTGGTATTGCTAAACCGGAGATGGATCATGCTTATGAGGTCATGAGTTCGTATGCAGCTGGAATGAATGCAGAGATCGA
DWV-SVN             TACCACATTGGACTATTCAATGTGGTATTGCTAAACCGGAGATGGATCATGCTTATGAGGTTATGAGTTCGTATGCAGCTGGAATGAATGCAGAGATTGA
```

```
                            6510      6520      6530      6540      6550      6560      6570      6580      6590      6600
                    ....|....|....|....|....|....|....|....|....|....|....|....|....|....|....|....|....|....|....|....|
Japan AB070959      AGCGCATGAACAAGTTCGGCGTTCATCAGTGGAATGTCAATATGCAGAGCCTCAAGCTCCAAGAAACCCTGATGACGAAGGACCAACTATAGATGAAGAA
USA1 AY291384       AGCGCATGAACAAGTTCGGCGTTCATCAGTGGAATGTCAATATGCAGAGCCTCAAGCTCCAAGAAATCCTGATGATGAAGGACCAACCATAGATGAAGAA
Chile JQ413340      AGCGCATGAACAAGTTCGGCGTTCATCAATGGAATGTCAATATGCAGAGCCTCAAGCTCCAAGAAATCCTGATGATGAAGGGCCAACCATAGATGAAGAA
Italy1 AJ489744     AGCGCATGAACAAGTTCGGCGTTCRTCAGTGGAATGTCAATTTGCAGAGCCTCAAGCTYCAAGAAAYCCTGATGATGAAGGACCAACCATAGATGAAGAA
France KX373899     AGCGCATGAACAAGTTCGGCGTTCATCAGTGGAATGTCAATATACAGAGCCTCAAGCTCCAAGAAATCCTGATGATGAAGGACCAACCATAGATGAAGAA
UK1 GU109335        AGCGCATGAACAAGTTCGGCGTTCATCAGTGGAATGTCAATATGTAGAGCCTCAAGCTCCAAGAAATCCTGATGATGAAGGACCTACCATAGATGAAGAA
UK2 KJ437447        AGCGCATGAACAAGTTCGGCGTTCATCAGTGGAATGTCAATATGCAGAGCCTCAAGCTCCAAGAAATCCTGATGATGAAGGACCAACCATAGATGAAGAA
Korea1 JX875304     AGCGCATGAACAAGTTCGGCGTTCATCAGTGGAATGTCAATATGCAGAGCCTCAAGCTCCAAGAAATCCTGACGATGAGGGACCAACCATAGATGAAGAA
Korea2 JX878305     AGCCCATGAACAAGTTCGGCGTTCATCAGTGGAATGTCAATATGCAGAGCCTCAAGCTCCAAGAAATCCTGATGATGAAGGACCAACCATAGATGAAGAA
China1 MF770715     AGCGCATGAACAAGTTCGGYGTTCATCAGTGGAATGCCAATATGCAGAGCCTCAAGGTTTAAGAAGGCTTGATGATGAAGGACCAACTATAGATGAAGAA
China2 MF036686     AGCGCATGAACAAGTTCGGCGTTCATCAGTGGAATGTCAATATGCAGAGCCTCAAGGTCTAAGAAAGCTTGATGATGAAGGACCAACTATAGATGAAGAA
China3 MH165180     AGCGCATGAACAAGTTCGGCGTTCATCAGTGGAATGTCAATATGCAGAGCCTCAAGGTCTAAGAAAGCTTGATGATGAAGGACCAACTATAGATGAAGAA
DWV-NVN             AGCACATGAGCAAGTTCGGCGTTCATCAGTGGAATGTCAATATGCAGAGCTTCAAGCTTTGAGAAAGCTTGATGATGAAGGACCAACCATAGATGAAGAA
DWV-SVN             AGCACATGAGCAAGTTCGGCGTTCATCAGTGGAATGTGAGTATGCAGAGCCTCAAGGCCTAAGAAAGCTTGATGATGAAGGACCAACCATAGATGAAGAA
```

```
                            6610      6620      6630      6640      6650      6660      6670      6680      6690      6700
                    ....|....|....|....|....|....|....|....|....|....|....|....|....|....|....|....|....|....|....|....|
Japan AB070959      CTTATGGGAGACACTGAATTTACATCACAGGCTCTAGAACGTCTTGTGGATGAAGGTTATATAACTGGAAAACAGAAGAAATACATAGCCACGTGGTGTA
USA1 AY291384       CTTATGGGCGACACTGAATTCACATCACAGGCTCTAGAACGTCTTGTGGATGAAGGTTATATAACTGGAAAACAGAAGAAATATATAGCTACGTGGTGTA
Chile JQ413340      CTTATGGGCGACACTGAATTTACATCACAGGCTCTAGAACGTCTTGTGGATGAAGGTTATATAACTGGAAAACAGAAGAAATATATAGCTACGTGGTGTA
Italy1 AJ489744     CTTATGGGCGACACTGAATTCACATCACAGGCTCTAGAACGTCTTGTGGATGAAGGTTATATAACTGGAAAACAGAAGAAATATATAGCTATGTGGTGTA
France KX373899     CTTATGGGCGACACTGAATTCACATCACAGGCTCTAGAACGACTTGTGGATGAAGGTTATATAACTGGAAAACAGAAGCAGTATATAGCTATGTGGTGTA
UK1 GU109335        CTTATGGGCGACACTGAATTCACATCACAGGCCCTAGAACGTCTTGTGGATGAAGGTTATATAACTGGAAAACAGAAGAAATATATAGCTACGTGGTGTA
UK2 KJ437447        CTTATGGGCGACACTGAATTCACATCACAGGCTCTAGAACGTCTTGTGGATGAAGGTTATATAACTGGAAAACAGAAGAAATATATAGCTACGTGGTGTA
Korea1 JX875304     CTTATGGGCGACACTGAATTTGCATCACAGGCTTTAGAACGTCTTGTGGATGAAGGTTATATAACTGGAAAACAGAAGAAGTATATAGCTACGTGGTGTA
Korea2 JX878305     CTTATGGGCGACACTGAATTTGCATCACAGGCTCTAGAACGTCTTGTGGATGAAGGTTATATAACTGGAAAACAGAAGAAGTATATAGCTACGTGGTGTA
China1 MF770715     CTTATGGGCGACACTGAATTTGCATCACAGGCTATAGAACGTCTTGTGGATGAAGGTTATATAACTGGAGAACAGAAGAAATATATAGCTACGTGGTGTA
China2 MF036686     CTTATGGGCGACGCTGAATTTACATCACTGGCTCTAGAACGTCTTGTGGATGAAGGTTATATAACTGGAGAACAGAAGAAATATATAGCTACGTGGTGTA
China3 MH165180     CTTATGGGCGACACTGAATTTACATCACTGGCTCTAGAACGTCTTGTGGATGAAGGTTATATAACTGGAGAACAGAAGAAATATATAGCTACGTGGTGTA
DWV-NVN             CTTATGGGTGACACTGAGTTTACATCACAAGCTTTAGAACGTCTTGTGGATGAAGGTTATATAACTGGAAAACAGAAGAAATATATAGCCACGTGGTGTA
DWV-SVN             CTTATGGGTGATACTGAATTTACATCACAAGCTCTAGAACGTCTTGTGGATGAAGGTTATATAACTGGAAAACAGAAGAAATATATAGCCACGTGGTGTA
```

```
                            6710      6720      6730      6740      6750      6760      6770      6780      6790      6800
                    ....|....|....|....|....|....|....|....|....|....|....|....|....|....|....|....|....|....|....|....|
Japan AB070959      GTAAGCGTCGTGAACATACTGCTGACTTTGATCTTGTGTGGACTGATAATTTGCGTGTGTTAAGTGCGTATGTGCATGAACGTTCAGCTTCAACTCGGCT
USA1 AY291384       GTAAGCGTCGTGAACATACTGCTGACTTTGATCTTGTGTGGACTGATAATTTGCGTGTGTTAAGCGCGTATGTGCATGAACGTTCATCTTCAACTCGGCT
Chile JQ413340      GTAAGCGTCGTGAACATACTGCTGACTTTGATCTTGTGTGGACTGATAATTTGCGTGTCTTGAGCGCGTATGTGCATGAACGTTCATCTTCAACTCGGCT
Italy1 AJ489744     GTAAGCGTCGTGAACATACTGCTGACTTTGATCTTGTGTGGACTGATAATTTGCGTGTGTTAAGTGCGTATGTGCATGAACGTTCATCTTCAACTCGGCT
France KX373899     GTAAGCGTCGTGAACATACAGCTGACTTTGATCTTGTGTGGACTGATAATTTGCGTGTGTTAAGTGCGTATGTGCATGATCGTTCATCTTCAACTCGGCT
UK1 GU109335        GTAAGCGTCGTGAACATACTGCTGACTTCGATCTTGTGTGGACTGATAATTTGCGTGTGTTAAGCGCGTATGTGCATGAACGTTCATCTTCAACTCGGCT
UK2 KJ437447        GTAAGCGTCGTGAACATACTGCTGACTTTGATCTTGTGTGGACTGATAATTTGCGTGTGTTAAGCGCGTATGTGCATGAACGTTCATCTTCAACTCGGCT
Korea1 JX875304     GTAAGCGTCGTGAACATTCTGCTGACTTTGATCTTGTGTGGACTGACAACCTGCGTGTGTTAAGTGCGTATGTGCTCGAACGTTCATCTTCAACTCGGCT
Korea2 JX878305     GTAAGCGTCGTGAACATGCTGCTGACTTTGATCTTGTGTGGACTGATAATTTGCGTGTGTTAAGTGCGTATGTGCACGAACGTTCATCTTCAACTCGGCT
China1 MF770715     GTAAGCGTCGCGAACATATTGCTGACTTTGATCTTGTGTGGACTGATAATTTGCGTGTGTTAAGTGCGTATGTGCATGAACGTTCATCTTCAACTCGGCT
China2 MF036686     GTAAGCGCCGTGAACATATTGCTGACTTTGATCTTGTGTGGACTGATAATTTGCGTGTGTTAAGTGCGTATGTGCATGAACGTTCATCTTCAACTCGGCT
China3 MH165180     GTAAGCGCCGTGAACATATTGCTGACTTTGATCTTGTGTGGACTGATAATTTGCGTGTGTTAAGTGCGTATGTGCATGAACGTTCATCTTCAACTCGGCT
DWV-NVN             GTAAGCGTCATGAGCATACTGCTGATTTTGATCTCGTGTGGACTGATAATTTGCGTGTGTTAAGTGCGTATGCGCATGAACGTTCATCTTCAACTCGGCT
DWV-SVN             GTAAGCGCCATGAGCATATTGCTGATTTCGACCTCGTGTGGACTGATAATTTGCGTGTGTTGAGTGCGTATGCGCATGAACGTTCATCTTCAACTCGGCT
```

```
                      6810      6820      6830      6840      6850      6860      6870      6880      6890      6900
                  ....|....|....|....|....|....|....|....|....|....|....|....|....|....|....|....|....|....|....|....|
Japan AB070959    TTCTACGGATGACGTCAAGTTATATAAAACAATTAGTATGTTGCATCAAAAGTATGATACCACAGAGTGTGCTAAGTGTCAACATTGGTATGCTCCGTTG
USA1 AY291384     TTCTACGGATGACGTCAAGTTATATAAAACGATTAGCATGTTACATCAAAAGTATGATACCACAGAGTGTGCTAAATGTCAACATTGGTATGCTCCGTTG
Chile JQ413340    TTCTACGGATGACGTCAAGTTATATAAAACGATTAGCATGTTACATCAAAAGTATGATACCACAGAGTGTGCTAAATGTCAACATTGGTATGCTCCGTTG
Italy1 AJ489744   TTCTACGGATGACGTCAAGTTATATAAAACGATTAGCATGTTACATCAAAAGTATGATACCACAGAGTGTGCTAAATGTCAACATTGGTATGCTCCGTTG
France KX373899   TTCTACGGATGACGTCAAATTATATAAAACGATTAGCATGTTACATCAAAAGTATGATACCACTGAGTGTGCTAAATGTCAACATTGGTATGCTCCGTTG
UK1 GU109335      TTCTACGGACGACGTTAAGTTATATAAAACGATTAGCATGTTACATCAAAAGTATGATACCACAGAGTGTGCTAAATGTCAACATTGGTATGCTCCGTTG
UK2 KJ437447      TTCTACGGACGACGTTAAGTTATATAAAACGATTAGCATGTTACATCAAAAGTATGATACCACAGAGTGTGCTAAATGTCAACATTGGTATGCTCCGTTG
Korea1 JX875304   TTCTACGGATGACGTCAAGTTATATAAAACAATTACCATGTTGCATCAAAAGTATGATACCTCAGAGTGTGCTAAATGTCAACATTGGTATGCTCCGTTG
Korea2 JX878305   TTCTACGGATGACGTCAAGTTATATAAAACAATTAGCATGTTGCATCAAAAGTATGATACCTCAGAGTGTGCCAAATGTCAACATTGGTATGCTCCGTTG
China1 MF770715   TTCTACGGATGATGTCAAGTTATATAAAACAATTAGCATGTTACATCAAAAGTATGATACCACAGAGTGTACTAAATGTCAACATTGGTATGCTCCGTTG
China2 MF036686   TTCTACGGATGACGCCAAATTATATAAAACAATTAGCATGTTACATCAAAAGTACGATACTACAGAGTGTGCTAAATGTCAACATTGGTATGCTCCGTTG
China3 MH165180   TTCTACGGATGACGTCAAATTATATAAAACAATTAGCATGTTACATCAAAAGTACGATACCACAGAGTGTGCTAAATGTCAACATTGGTATGCTCCGTTG
DWV-NVN           TTCTACGGATGACGTTAAGTTGTACAAAACAATTAGTATGTTACATCAAAAATATGATACTACAGAGTGTGCGAAATGTCAACACTGGTATGCTCCACTA
DWV-SVN           TTCTACGGATGACGTTAAGTTGTATAAAACAATTAGTATGTTACATCAAAAATATGATACTACAGAGTGCGCGAAATGTCAACACTGGTACGCTCCACTA

                      6910      6920      6930      6940      6950      6960      6970      6980      6990      7000
                  ....|....|....|....|....|....|....|....|....|....|....|....|....|....|....|....|....|....|....|....|
Japan AB070959    ACTGATATCTATGTTGATGACAAGAAATTGTTTTGGTGTCAGAAAGAGAAAAAGACACTTATTGATGTCCGAAAATTGTCGAAAGAAGATGTGACTGTTC
USA1 AY291384     ACTGATATCTATGTTGATGACAAGAAATTGTTCTGGTGTCAGAAAGAGAAAAAGACACTTATTGATGTCCGAAAATTGTCGAAAGAAGATGTGACTGTTC
Chile JQ413340    ACTGATATCTATGTTGATGACAAGAAATTGTTCTGGTGTCAGAAAGAGAAAAAGACACTTATTGATGTCCGAAAATTGTCGAAAGAAGATGTGACTGTTC
Italy1 AJ489744   ACTGATATCTATGTTGATGACAAGAAATTGTTYTGGTGTCAGAAAGAGAAAAAGACACTTATTGATGTCCGAAAATTGTCGAAAGAAGATGTGACTGTTC
France KX373899   ACTGATATCTACGTTGATGACAAGAAATTGTTCTGGTGTCAGAAAGAGAAAAAGACACTTATTGATGTCCGTAAATTGTCGAAAGAAGATGTGACTGTTC
UK1 GU109335      ACTGATATCTATGTTGATGACAAAAAATTGTTTTGGTGTCAGAAAGAGAAAAAGACACTTATTGATGTCCGAAAATTGTCGAAAGAAGATGTGACTGTTC
UK2 KJ437447      ACTGATATCTATGTTGATGACAAAAAATTGTTCTGGTGTCAGAAAGAGAAAAAGACACTTATTGATGTCCGAAAATTGTCGAAAGAAGATGTGACTGTTC
Korea1 JX875304   ACTGATGTCTATGTTGATGACAAGAAATTGTTTTGGTGTCAGAAAGAGAAAAAGACACTTATTGATGTCCGAAAGTTGTCGAAAGAAGATGTGACTGTTC
Korea2 JX878305   ACTGATATCTACGTTGATGACAAGAAATTGTTTTGGTGTCAGAAAGAGAAAAAGACACTTATTGATGTCCGAAAGTTGTCGAAAGAAGATGTGACTGTTC
China1 MF770715   ACTGATATCTATGTTGATGACAAGAAACTGTTTTGGAATCAGAAGGAGAAAAAGACACTTATTGATGTTCGAAAATTGTCGAAAGAAGATGTGACTGTTC
China2 MF036686   ACTGACATCTATGTTGATGATAAGAAATTGTTTTGGTGTCAGAAAGAGAAAAAGACACTTATTGATGTCCGAAAATTGTCGAAAGAAGATGTAACTGTTC
China3 MH165180   ACAGATATCTATGTTGATGACAAGAAATTGTTTTGGTGTCAGAAAGAGAAAAAGACACTTATTGATGTCCGAAAATTGTCGAAAGAAGATGTAACTGTTC
DWV-NVN           ACAGCTATTTATGTAGATGATAAGAAATTATTTTGGTGTCGAAAAGAGACGAAGACACTTATCGATGTCAGTAAATTGTCGAAAGAAGATGTGACTGTTC
DWV-SVN           ACAGCTATTTATGTAGATGACAAGAAATTATTTTGGTGTCGGAAAGAGAAGAAGACACTTATCGATGTCAGTAAATTGTCGAAAGAAGATGTCACTGTTC

                      7010      7020      7030      7040      7050      7060      7070      7080      7090      7100
                  ....|....|....|....|....|....|....|....|....|....|....|....|....|....|....|....|....|....|....|....|
Japan AB070959    AATCAAAATTGATTAATTTGTCTGTTCCATGTGGTGAAGTGTGTATGTTACATTCAAAATATTTCAATTATCTTTTCCATAAAGCATGGTTGTTTGAGAA
USA1 AY291384     AATCGAAATTGATTAATTTATCTGTTCCTTGTGGTGACGTATGTATGTTACATTCAAAATATTTCAATTATCTTTTCCATAAAGCATGGTTGTTCGAGAA
Chile JQ413340    AATCGAAATTGATTAATTTATCTGTTCCTTGTGGTGAAGTATGTATGTTACATTCAAAATATTTCAATTATCTTTTCCATAAAGCATGGTTGTTCGAGAA
Italy1 AJ489744   AATCRAAATTGRTTAATTTATCTGTTCCTTGTGGTGAAGTKTGTATGTTACATTCAAAATATTTYAATTATCTTTTCCATAAAGCATGGTTGTTTGAGAA
France KX373899   AATCGAAATTGATTAATTTATCTGTTCCTTGTGGTGAAGTATGCATGTTACATTCAAAATATTTCAATTATCTTTTTCATAAAGCATGGTTGTTTGAAAA
UK1 GU109335      AATCAAAATTGATTAATTTATCTGTTCCTTGTGGTGAAGTGTGTATGTTACATTCAAAATATTTCAATTATCTTTTCCATAAAGCATGGTTGTTTGAGAA
UK2 KJ437447      AATCAAAATTAATTAATTTATCTGTTCCTTGTGGTGAAGTGTGTATGTTACATTCAAAATATTTCAATTATCTTTTCCATAAAGCATGGTTGTTTGAGAA
Korea1 JX875304   AATCAAAATTGATTAATTTATCTGTTCCTTGTGGTGAAGTGTGTGTGTTACATTCAAAATATTTCAATTATCTTTTCCATAAAGCATGGTTGTGTGAGAA
Korea2 JX878305   AATCAAAATTGATTAATTTATCTGTGCCTTGTGGCGAAGTGTGTGTGTTACATTCAAAATATTTCAATTATCTTTTCCATAAAGCATGGTTGTGTGAGAA
China1 MF770715   AATCAAAATTGATTAACTTATCTGTTCCTTGTGGTGAAGTGTGTGTGTTACATTCAAAATATTTCAATTATCTTTTCCATAAAGCATGGTTATTAGAGAA
China2 MF036686   AATCAAAATTGATTAACTTATCTGTTCCTTGTGGTGAAGTGTGTGTGTTACATTCAAAATATTTCAATTATCTTTTCCATAAAGCATGGTTGTGTGAGAA
China3 MH165180   AATCAAAATTGATTAACTTATCTGTTCCTTGTGGTGAAGTGTGTGTGTTACATTCAAAATATTTCAATTATCTTTTCCATAAAGCATGGTTGTGTGAGAA
DWV-NVN           AGTCAAAATTGATTAACTTGTCTGTTCCTTGTGGTGAAGTGTGTATGTTACATTCAAAATATTTCAATTATCTTTTTCATAAAGCATGGTTGTTTGAGAA
DWV-SVN           AGTCAAAATTGATTAACTTGTCTGTTCCTTGTGGTGAAGTGTGTATGTTACATTCAAAATATTTCAATTATCTTTTTCATAAAGCATGGTTGTTTGAGAA

                      7110      7120      7130      7140      7150      7160      7170      7180      7190      7200
                  ....|....|....|....|....|....|....|....|....|....|....|....|....|....|....|....|....|....|....|....|
Japan AB070959    CCCAACTTGGCGCCTAATATATAATGGTACTAAGAAGGGTATGCCCGAGTACTTTATGAATTGTGTGGATGAAATTTCATTAGATTCCAAATTTGGTAAA
USA1 AY291384     CCCAACTTGGCGCCTAATATATAATGGTACCAAGAAGGGTATGCCTGAGTACTTTATGAATTGTGTGGATGAAATATCATTAGATTCCAAATTTGGTAAA
Chile JQ413340    CCCAACTTGGCGCCTAATATATAATGGTACCAAGAAGGGTATGCCTGAGTACTTTATGAATTGTGTGGATGAGATATCATTAGATTCTAAATTTTGTAAA
Italy1 AJ489744   CCCAACTTGGCGCCTAATATATAATGGTACCAAGAAGGGTATGCCCGAGTACTTTATGAATTGTGTGGATGAAATTTCATTAGATTCCAAATTTGGTAAA
France KX373899   CCCAACTTGGCGCCTAATATATAATGGTACCAAGAAGGGTATGCCCGAGTACTTTATGAATTGTGTGGATGAAATTTCATTAGATTCCAAATTTGGTAAA
UK1 GU109335      CCCAACTTGGCGCCTAATATATAATGGTACCAAGAAGGGTATGCCCGAGTACTTTATGAATTGTGTGGATGAAATTTCATTAGATTCTAAATTCGGTAAA
UK2 KJ437447      CCCAACTTGGCGCCTAATATATAATGGTACCAAGAAGGGTATGCCTGAGTACTTTATGAATTGTGTGGATGAAATTTCATTAGATTCCAAATTCGGTAAA
Korea1 JX875304   CCCAACTTGGCGCCTAATATATAATGGTACCAAGAAGGGTATGCCCGAGTACTTTATGAATTGTGTGGATGAGATTTCATTAGATTCCAAATTTGGTAAA
Korea2 JX878305   CCCAACTTGGCGCCTAATATATAATGGTACCAAGAAGGGTATGCCCGAGTACTTTATGAATTGTGTGGATGAAATTTCATTAGATTCTAAATTTGGTAAA
China1 MF770715   CCCAACATGGCGCCTAATATATAATGGTTCCAAGAAGGGTATGCCCGAGTACTTTATGAATTGTGTGGATGAAATTTCATTAGATTCTAAATTTGGTAAA
China2 MF036686   CCCGACTTGGCGCCTAATATATAATGGTACCAAGAAGGGTATGCCCGAGTACTTTATGAATTGTGTGGATGAAATTTCATTAGATTCTAAATTTGGTAAA
China3 MH165180   CCCAACTTGGCGCCTAATATATAATGGTACCAAGAAGGGTATGCCCGAGTACTTTATGAATTGTGTGGATGAAATTTCATTGGATTCTAAATTTGGTAAA
DWV-NVN           CCCGACTTGGCGTTTAATATATAATGGTACTAAGAAAGGTATGCCTGAATATTTTATGAATTGCGTGGATGAAATTTCATTAGATTCTAAGTTTGGTAAA
DWV-SVN           CCCGACTTGGCGTTTAATATATAATGGTACTAAGAAAGGTATGCCTGAATATTTTATGAATTGTGTGGATGAAATTTCATTAGATTCTAAGTTTGGTAAA
```

```
                             7210      7220      7230      7240      7250      7260      7270      7280      7290      7300
                         ....|....|....|....|....|....|....|....|....|....|....|....|....|....|....|....|....|....|....|....|
Japan AB070959           GTGAAAGTGTGGTTGCAAGCGATCATTGATAAGTATTTAACCCGTCCCGTGAAAATGATTCGTGATTTTCTTTTCAAGTGGTGGCCGCAAGTTGCGTATG
USA1 AY291384            GTGAAAGTATGGTTGCAAGCGATCATTGATAAGTATTTAACTCGTCCCGTGAAAATGATTCGTGATTTTCTTTTCAAGTGGTGGCCGCAAGTTGCGTATG
Chile JQ413340           ATGAAAGTATGGTTGCAAGCGATCATTGATAAGTATTTAACTCGTCCCGTGAAAATGATTCGTGATTTTCTTTTCAAGTGGTGGCCGCAAGTTGCGTATG
Italy1 AJ489744          GTGAAAGTGTGGTTGCAAGCGATCATTGATAAGTATTTAACTCGTCCCGTGAAAATGATTCGTGATTTTCTTTTCAAGTGGTGGCCGCAAGTTGCGTATG
France KX373899          GTGAAAGTGTGGTTGCAAGCGATCATCGATAAGTATTTAACTCGTCCCGTGAAAATGATTCGTGATTTTCTTTTCAAGTGGTGGCCGCAAGTTGCGTATG
UK1 GU109335             GTGAAAGTGTGGTTGCAAGCGATCATTGATAAGTATTTAACTCGTCCTGTGAAAATGATTCGTGATTTTCTTTTCAAGTGGTGGCCGCAAGTTGCGTATG
UK2 KJ437447             GTGAAAGTATGGTTGCAAGCGATCATTGATAAGTATTTAACTCGTCCCGTGAAAATGATTCGTGATTTTCTTTTCAAGTGGTGGCCGCAAGTTGCGTATG
Korea1 JX875304          GTGAGAGTGTGGTTGCAAGCGATCATTGATAAGTATTTAACTCGTCCCGGGAAAATGATTCGTGATTTTCTTTTCAAGTGGTGGCCGCAAGTTGCGTATG
Korea2 JX878305          GTGAAAGTGTGGTTGCAAGCGATCATCGATAAGTATTTAACTCGTCCCGTGAAAATGATTCGTGATTTTCTTTTCAAGTGGTGGCCGCAAGTTGCGTACG
China1 MF770715          GTGAAAGTGTGGTTGCAAGCGATCATTGATAAGTATTTAACTCGTCCCGTGAAAATGATTCGTGATTTTCTTTTCAAGTGGTGGCCGCAAGTTGCGTATG
China2 MF036686          GTGAAAGTGTGGTTGCAAGCGATTATTGATAAGTATTTAACTCGTCCCGTGAAGATGATTCGTGATTTTCTTTTCAAGTGGTGGCCGCAAGTTGCGTATG
China3 MH165180          GTGAAAGTGTGGTTGCAAGCGATTATTGATAAGTATTTAACTCGTCCTGTGAAAATGATTCGTGATTTTCTTTTCAAGTGGTGGCCGCAAGTTGCGTATG
DWV-NVN                  GTGAAAGTGTGGCTGCAAGCAATCATCGATAAATATTTGACTCGTCCTGTGAAAATGATTCGTGATTTTCTTTTCAAGTGGTGGCCGCAAGTCGCGTATG
DWV-SVN                  GTGAAGGTGTGGCTGCAAGCAATCATCGATAAATATTTGACTCGTCCTGTGAAAATGATTCGTGATTTTCTTTTCAAGTGGTGGCCGCAAGTTGCGTATG

                             7310      7320      7330      7340      7350      7360      7370      7380      7390      7400
                         ....|....|....|....|....|....|....|....|....|....|....|....|....|....|....|....|....|....|....|....|
Japan AB070959           TGTTGAGCTTGCTAGGTATAATTGGTATAACAGCGTATGAAATGAGAAATCCGAAACCAACTTCTGAGCAATTAGCTGATCATTATGTAAATAGGCATTG
USA1 AY291384            TGTTGAGCTTGCTAGGTATAATTGGTATAACTGCGTATGAAATGAGAAATCCGAAACCAACTTCTGAGGAATTAGCTGATCATTATGTGAATAGGCATTG
Chile JQ413340           TGTTGAGCTTGCTAGGTATAATCGGTATAACTGCGTATGAAATGAGAAATCCGAAACCAACTTCTGAGGAATTAGCTGATCATTATGTGAATAGGCATTG
Italy1 AJ489744          TGTTGAGCTTGCTAGGTATAATYGGTATAACTGCGTATGAAATGAGAAATCCGAAACCAACTTCTGAGGAATTAGCTGATCATTATGTGAATAGGCATTG
France KX373899          TGTTGAGCTTGCTAGGTATAATCGGTATAACTGCGTATGAAATGAGAAATCCGAAACCAACTTCTGAGGAATTAGCTGATCATTATGTGAATAGGCATTG
UK1 GU109335             TGTTGAGCTTGTTAGGCATAATCGGTATAACTGCGTATGAAATGAGAAATCCGAAACCAACTTCTGAGGAATTAGCTGATCATTATGTGAATAGGCATTG
UK2 KJ437447             TGTTGAGCTTGCTAGGTATAATTGGTATAACTGCGTATGAAATGAGAAATCCGAAACCAACGTCTGAGGAATTAGCTGATCATTATGTGAATAGGCATTG
Korea1 JX875304          TGTTGAGCTTGCTAGGTATAATTGGTATAACTGCGTATGAAATGAGAAATCCGAAACCCACTTCTGAGGAGTTAGCTGATCACCGTGTGAATAGGCATTG
Korea2 JX878305          TGTTGAGCTTGCTAGGTATAATTGGTATAACTGCGTATGAAATGAGAAATCCCAAACCAACTTCTGAGGAATTAGCTGATCATTATGTGAATAGGCATTG
China1 MF770715          TGTTGAGCTTGTTAGGTATAATTGGTATAACTGCGTATGAAATGAGAAATCCGAAACCAACTTCTGAGGAACTGGCTGATCATTATGTGAATAGGCATTG
China2 MF036686          TGTTGAGCTTGTTAGGTATAATTGGTATAACTGCGTATGAAATGAGAAATCCGAAACCAACTTCTGAGGAACTGGCTGATCATTATGTGAATAGGCATTG
China3 MH165180          TGTTGAGCTTGTTAGGCATAATTGGTACAACTGCGTATGAAATGAGAAATCCGAAACCAGCTTCCGAGGAACTGACTGATCATTATGTGAATAGGCATTG
DWV-NVN                  TGTTAAGCTTGTTAGGTATAATTGGTATAACTGCGTATGAAATGAGAAATCCGAAACCGACTTCTGAAGAATTGGCTGATCATTATGTGAATAGACATTG
DWV-SVN                  TGTTAAGTTTGTTAGGTATAATTGGTATAACTGCGTATGAAATGAGAAATCCGAGACCGACTTCTGAAGAATTGGCTGATCATTATGTGAATAGACATTG

                             7410      7420      7430      7440      7450      7460      7470      7480      7490      7500
                         ....|....|....|....|....|....|....|....|....|....|....|....|....|....|....|....|....|....|....|....|
Japan AB070959           TAGCTCTGATTTTTGGTCACCAGGGTTGGCATCACCTCAAGGATTGAAATATAGTGAAGCAGTAACAGCAAAGGCACCTAGAATTCATAGATTGCCAGTT
USA1 AY291384            TAGCTCTGATTTTTGGTCACCAGGACTGGCATCACCTCAAGGATTGAAATATAGTGAAGCAGTAACAGTAAAGGCACCTAGAATCCATAGATTGCCAGTT
Chile JQ413340           TAGCTCTGATTTTTGGTCACCAGGACTGGCATCACCTCAAGGATTGAAATATAGTGAAGCAGTAACAGTAAAGGCGCCAAGAATCCATAGATTGCCAGTT
Italy1 AJ489744          TAGCTCTGATTTTTGGTCACCAGGACTGGCATCACCTCAAGGATTGAAATATAGTGAAGCAGTAACAGTAAAGGCACCTAGAATCCATAGATTGCCAGTT
France KX373899          TAGCTCTGATTTTTGGTCACCAGGACTGGCATCACCTCARGGATTGAAGTATAGTGAAGCAGTGACAGTAAAGGCACCTAGAATTCATAGATTGCCAGTT
UK1 GU109335             TAGCTCTGATTTTTGGTCACCAGGACTGGCATCACCTCAAGGATTGAAGTATAGTGAAGCAGTAACAGTAAAGGCACCTAGAATCCATAGATTGCCAGTT
UK2 KJ437447             TAGCTCTGATTTTTGGTCACCAGGACTGGCATCACCTCAAGGATTGAAATATAGTGAAGCAGTAACAGTAAAGGCACCTAGAATCCATAGATTGCCAGTT
Korea1 JX875304          TACCTCTGATTTTTGCTCACCAGGACAGGCATCACCTCAAGGATTGAAATATCGTGTAGCAGTAACAGACAAGGCACCTAGAATTCATAGATTGCCAGTC
Korea2 JX878305          TAGCTCTGATTTTTGGTCACCAGGACTGGCATCACCTCAAGGATTGAAATATAGTGAAGCAGTAACAGCAAAGGCACCTAGAATCCATAGATTGCCAGTT
China1 MF770715          TAGCCCTGATTTCTGGTCACCAGGAATGGCAACACCTCAAGGACTGAAATATAGTGAAGCAGTAACAGCAAGGGCACCTAGAATTCATAGATTGCCAGTT
China2 MF036686          TAGCTCTGATTTTTGGTCACCAGGAATGGCAACACCTCAAGGACTGAAATATAGTGAAGCAGTAACAGCAAAGGCGCCTAGAATCCATAGATTGCCAGTT
China3 MH165180          TAGCTCTGATTTTTGGTCACCAGGAATGGCAACACCTCAAGGACTGAAATATAGTGAAGCAGTAACAGCAAAGGCACCTAGAATCCATAGATTGCCAGTT
DWV-NVN                  TAGCTCTGATTTTTGGTCTCCAGGACTGGCATCACCCCAGGGATTGAAATATAGTGAAGCAGTAACAGCAAAAGCACCTAGAATTCATAGATTGCCGGTT
DWV-SVN                  TAGCTCTGATTTTTGGTCTCCAGGACTGGCATCACCCCAGGGATTGAAATATAGTGAAGCAGTAACAGCAAAAGCACCTAGAATCCATAGATTGCCGGTT

                             7510      7520      7530      7540      7550      7560      7570      7580      7590      7600
                         ....|....|....|....|....|....|....|....|....|....|....|....|....|....|....|....|....|....|....|....|
Japan AB070959           ACTACTAAGCCTCAGGGATCAACACAACAAGTAGATGCTGCTGTGAATAAAATTTTACAAAACATGGTTTACATTGGTGTTGTTTTCCCAAAAGTGCCTG
USA1 AY291384            ACTACTAAGCCTCAGGGATCAACACAACAAGTAGACGCTGCTGTGAATAAAATTCTACAGAACATGGTTTATATTGGTGTTGTTTTCCCAAAAGTGCCTG
Chile JQ413340           ACTACTAAGCCTCAAGGATCAACACAACAAGTAGACGCTGCTGTGAATAAGATTCTACAGAACATGGTTTATATTGGTGTTGTTTTCCCAAAAGTGCCTG
Italy1 AJ489744          ACTACTAAGCCTCAGGGATCAACACAACAAGTAGACGCTGCTGTGAATAAAATTTTACAGAAYATGGTTTACATTGGTGTTGTTTTCCCAAAAGTGCCTG
France KX373899          ACTACTAAGCCTCAGGGATCAACACAACAAGTAGACGCTGCTGTGAATAAAATTCTACAGAACATGGTTTATATTGGTGTTGTTTTCCCAAAAGTGCCTG
UK1 GU109335             ACTACTAAGCCTCAGGGATCAACACAACAAGTAGACGCTGCTGTGAATAAAATTTTGCAGAACATGGTTTACATTGGTGTTGTTTTTCCGAAAGTGCCTG
UK2 KJ437447             ACTACTAAGCCTCAGGGATCAACACAACAAGTAGACGCTGCTGTGAATAAAATTCTACAGAATATGGTTTATATTGGTGTTGTTTTCCCAAAAGTGCCTG
Korea1 JX875304          ACTACTAGGCCTCAGGGATCAACACAACAAGTAGACGCTGCTGTGAATAAAATTCTACAGAATATGGTTTACATTGGTGTTGTTTTCCCAAAAGTGCCTG
Korea2 JX878305          ACTACTAAGCCTCAGGGATCAACACAACAAGTAGACGCTGCTGTGAATAAAATTTTACAGAATATGGTCTACATTGGTGTTGTTTTCCCAAAAGTGCCTG
China1 MF770715          ACTACTAAGCCTCAGGGATCAACACAACAAGTAGACGCTGCTGTGAATAAAATTTTACAGAACATGGTTTATATTGGTGTTGTTTTCCCGAAAGTGCCTG
China2 MF036686          ACTACTAGGCCTCAGGGCTCAACGCAACAAGTAGACGCTGCTGTGAATAAAATTTTACAGAACATGGTTTACATTGGTGTTGTTTTCCCAAAAGTGCCTG
China3 MH165180          ACTACTAAGCCTCAGGGATCAACACAACAAGTAGACGCTGCTGTGCATAAAATTCTACAGAACATGGTTTACATTGGTGTTGTCTTCCCAAAAGTGCCTG
DWV-NVN                  ACTACTAAGCCTCAAGGATCAACGCAACAAGTAGATGCAGCTGTGAACAAAATTTTACAGAATATGGTCTATATTGGTGTTGTTTTTCCGAAAGTACCTG
DWV-SVN                  ACTACTAAGCCTCAAGGATCAACGCAACAAGTAGATGCAGCTGTGAACAAAATCTTACAGAATATGGTCTATATTGGCGTTGTTTTTCCGAAAGTACCTG
```

```
                        7610      7620      7630      7640      7650      7660      7670      7680      7690      7700
                ....|....|....|....|....|....|....|....|....|....|....|....|....|....|....|....|....|....|....|....|
Japan AB070959  GTAGTAAGTGGCGAGATATTAATTTTAGGTGTCTTATGCTTCATAATAGGCAATGTTTAATGTTGAGGCATTATATTGAGTCAACTGCCGCCTTTCCTGA
USA1 AY291384   GTAGTAAGTGGCGAGATATTAATTTTAGGTGTCTTATGCTTCATAATAGGCAATGTTTAATGTTGAGGCATTATATTGAGTCAACTGCCGCCTTTCCTGA
Chile JQ413340  GTAGTAAGTGGCGAGATATTAATTTTAGGTGTCTTATGCTTCATAATAGGCAATGTTTAATGTTGAGGCATTATATTGAGTCAACTGCTGCCTTTCCTGA
Italy1 AJ489744 GTAGTAAGTGGCGAGATATTAATTTTAGGTGTCTTATGCTTCATAATAGGCAATGTTTAATGTTGAGGCATTATATTGAGTCRACTGCTGCCTTTCCTGA
France KX373899 GTAGTAAGTGGCGAGATATTAATTTTAGGTGTCTTATGCTTCATAATAGGCAATGTTTAATGTTGAGGCATTATATTGAGTCAACTGCCGCCTTTCCTGA
UK1 GU109335    GTAGTAAGTGGCGAGATATTAATTTTAGGTGTCTTATGCTTCATAATAGGCAATGTTTAATGTTGAGGCATTATATTGAGTCAACTGCCGCCTTTCCTGA
UK2 KJ437447    GTAGTAAGTGGCGAGATATTAATTTTAGGTGTCTTATGCTTCATAATAGGCAATGTTTAATGTTGAGGCATTATATTGAGTCAACAGCTGCCTTTCCTGA
Korea1 JX875304 GTAGTAAGTGGCGAGATATTAATTTTAGATGTCTTATGCTTCATAACAGGCAATGTTTAATGTTGAGGCATTATATTGAGTCAACTGCCGCCTTTCCTGA
Korea2 JX878305 GTAGTAAGTGGCGAGATATTAATTTTCGGTGTCTTATGCTCCATAATAGGCAATGTTTGATGTTGAGGCATTATATTGAGTCAACTGCTGCCTTTCCCGA
China1 MF770715 GTAGTAAGTGGCGAGATATTAATTTTAGGTGTCTTATGCTTCATAATAGGCAATGTTTAATGTTGAGGCATTATCTTGAGTCAACTGCCGCCTTTCCTGA
China2 MF036686 GTAGTAAGTGGCGAGATATTAATTTTAGGTGTCTTATGCTTCATAATAGGCAATGTTTAATGTTGAGGCATTATATTGAGTCAACTGCCGCCTTTCCTGA
China3 MH165180 GTAGTAAGTGGCGAGATATTAATTTTAGGTGTCTTATGCTTCATAATAGGCAATGTTTAATGTTGAGGCATTATCTCGAGTCAACTGCCTCCTTTCCTGA
DWV-NVN         GTAGTAAGTGGCGAGATATTAATTTTAGATGTCTTATGCTTCACAATAGGCAATGTTTGATGTTGAGGCATTATATTGAGTCAACTGCCGCTTTTCCTGA
DWV-SVN         GTAGTAAGTGGCGAGATATTAATTTTAGATGTCTTATGCTTCACAATAGGCAATGTTTGATGTTGAGGCATTATATTGAGTCAACTGCCGCTTTTCCCGA

                        7710      7720      7730      7740      7750      7760      7770      7780      7790      7800
                ....|....|....|....|....|....|....|....|....|....|....|....|....|....|....|....|....|....|....|
Japan AB070959  GGGAACCAAGTACTATTTTAAGTATATTCATAATCAAGAGACTAGAATGTCTGGTGATATTTCTGGTATTGAAATTGATTTGTTGAATTTGCCTAGATTG
USA1 AY291384   GGGAACCAAGTACTATTTTAAGTATATTCATAATCAAGAGACTAGAATGTCTGGTGATATTTCTGGTATTGAAATTGATTTGTTGAATTTACCTAGATTG
Chile JQ413340  GGGAACCAAGTACTATTTTAAGTATATTCATAATCAAGAGACTAGAATGTCTGGTGATATTTCTGGTATTGAAATTGATTTGTTGAATTTACCTAGATTG
Italy1 AJ489744 GGGAACCAAGTACTATTTYAAGTATATTCATAATCAAGAGACTAGAATGTCTGGTGATATTTCTGGTATTGAAATTGATTTGTTGAATTTACCTAGATTG
France KX373899 GGGAACCAAGTACTATTTTAAGTATATTCATAATCAAGAGACTAGAATGTCTGGTGACATTTCTGGTATTGAAATTGATTTGTTGAATTTACCTAGATTG
UK1 GU109335    GGGAACCAAGTACTATTTTAAGTATATTCATAATCAAGAGACTAGAATGTCTGGTGATATTTCTGGTATTGAAATTGATTTGTTGAATTTACCTAGATTG
UK2 KJ437447    GGGAACCAAGTACTATTTTAAGTATATTCATAATCAAGAGACTAGAATGTCTGGTGATATTTCTGGTATTGAAATTGATTTGTTGAATTTACCCAGATTG
Korea1 JX875304 GGGAACCAAGTACTATTTTAAGTATATTCATAATCAAGAGACTAGAATGTCTGGTGATATTTCTGGTATTGAAATTGATTTGTTGAATTTGCCTAGATTG
Korea2 JX878305 GGGAACCAAGTACTATTTTAAGTATATTCATAATCAAGAGACTAGAATGTCCGGTGATATTTCTGGTATTGAAATTGATTTGTTGAATTTACCTAGATTG
China1 MF770715 GGGAACCAAGTACTATTTTAAGTATATTCATAATCAAGAGACTAGAATGTCTGGTGATATTTCTGGTATTGAAATTGATTTGTTGAATTTACCTAGATTG
China2 MF036686 GGGAACCAAGTATTATTTTAAGTATATTCATAATCAAGAGACTAGAATGTCTGGTGATATTTCTGGTATTGAAATTGATTTATTGAATTTGCCTAGATTG
China3 MH165180 GGGAACCAAGTACTATTTTAAGTATATTCATAATCAAGAGACTAGAATGTCTGGTGATATTTCTGGTATTGAAATCGATTTGTTGAATTTACCTAGATTG
DWV-NVN         GGGCACCAAATACTATTTCAAGTATATTCATAATCAAGAAACCAGAATGTCAGGTGATATATCTGGGATTGAAATCGATTTATTGAATTTGCCAAGATTG
DWV-SVN         GGGCACCAAATACTATTTTAAGTATATTCATAATCAAGAAACCAGAATGTCAGGTGATATATCTGGGATTGAAATTGATTTATTGAATTTGCCAAGATTG

                        7810      7820      7830      7840      7850      7860      7870      7880      7890      7900
                ....|....|....|....|....|....|....|....|....|....|....|....|....|....|....|....|....|....|....|
Japan AB070959  TATTATGGTGGTCTCGCGGGAGAGGAGTCATTTGATAGCAATATYGTGCTTGTGACTATGCCTAATCGTATTCCTGAGTGTAAGAGCATTATTAAATTTA
USA1 AY291384   TATTATGGTGGTCTCGCGGGAGAGGAGTCGTTTGATAGTAATATCGTGCTTGTGACTATGCCTAATCGTATTCCTGAGTGTAAGAGCATTATTAAATTTA
Chile JQ413340  TATTATGGTGGTCTCGCGGGAGAGGAGTCGTTTGATAGTAATATCGTGCTTGTGACTATGCCTAATCGTATTCCTGAGTGTAAGAGCATTATTAAATTTA
Italy1 AJ489744 TATTATGGTGGTCTCGCGGGAGAGGAGTCATTTGATAGCAATATCGTGCTTGTGACTATGCCTAATCGTATTCCTGAGTGTAAGAGCATTATTAAATTTA
France KX373899 TATTATGGTGGTCTCGCGGGAGAGGAGTCATTTGATAGCAATATCGTGCTTGTGACTATGCCTAATCGTATTCCTGAGTGCAAGAGCATTATTAAATTTA
UK1 GU109335    TATTATGGTGGTCTCGCGGGAGAGGAGTCATTTGATAGCAATATTGTGCTTGTGACTATGCCTAATCGTATTCCTGAGTGTAAGAGCATTATTAAATTTA
UK2 KJ437447    TATTATGGTGGTCTTGCGGGAGAGGAGTCATTTGATAGCAATATCGTGCTTGTGACTATGCCTAATCGTATTCCTGAGTGTAAGAGCATTGTTAAATTTA
Korea1 JX875304 TATTATGGTGGTCTCGCGGGAGAGGAGTCATTTGATAGCAATATCGTGCTTGTGACTATGCCTAATCGTATTCCTGAGTGTAAGAGCATTATTAAATTTA
Korea2 JX878305 TATTATGGTGGTCTCGCGGGAGAGGAGTCATTTGATAGCAATATCGTGCTTGTGACTATGCCTAATCGTATTCCTGAGTGTAAGAGCATTATTAAATTTA
China1 MF770715 TACTATGGTGGTCTCGCGGGAGAGGAGTCATTTGATAGCAATATCGTGCTTGTGACTATGCCTAATCGTATTCCTGAGTGTAAGAGCATTATTAAATTTA
China2 MF036686 TATTATGGTGGTCTCGCGGGAGAGGAGTCATTTGATAGCAATATCGTGCTTGTGACTATGCCTAATCGTATTCCTGAGTGTAAGAGCATTATTAAATTTA
China3 MH165180 TATTATGGTGGTCTCGCGGGAGAGGAGTCATTTGATAGCAATATCGTGCTTGTGACTATGCCTAATCGTATTCCTGAGTGTAAGAGCATTGTTAAATTTA
DWV-NVN         TATTATGGTGGTCTTGCAGGAGAAGAGTCGTTTGATAGCAATATTGTGCTTGTGACTATGCCTAATCGTATACCTGAGTGTAAGAGCATTATAAAGTTCA
DWV-SVN         TATTATGGTGGTCTTGCAGGAGAAGAGTCGTTTGATAGCAATATTGTGCTTGTGACTATGCCTAATCGTATACCTGAGTGTAAGAGCATTATAAAGTTCA

                        7910      7920      7930      7940      7950      7960      7970      7980      7990      8000
                ....|....|....|....|....|....|....|....|....|....|....|....|....|....|....|....|....|....|....|
Japan AB070959  TAGCGTCACATAATGAACATATACGTGCTCAGAATGATGGAGTGTTAGTAACTGGCGACCATACTCAGCTGTTGGCTTTCGAGAATAATAATAAAACTCC
USA1 AY291384   TAGCGTCACATAATGAACATATACGTGCTCAGAATGATGGAGTGTTAGTAACTGGCGACCATACTCAGCTATTGGCTTTCGAGAATAATAATAAAACTCC
Chile JQ413340  TAGCGTCACATAATGAGCATATACGTGCTCAGAATGATGGAGTGTTAGTAACTGGCGACCATACTCAGCTATTGGCTTTCGAGAATAATAATAAAACTCC
Italy1 AJ489744 TAGCGTCACATAATGAACATATACGTGCTCAGAATGATGGAGTGTTAGTAACTGGCGACCATACTCAGTTATTGGCTTTCGAGAATAATAATAAGACCCC
France KX373899 TAGCGTCACATAATGAACATATGCGTGCTCAGAATGACGGAGTGTTAGTAACTGGCGACCATACTCAGCTATTGGCTTTCGAGAATAATAATAAGACTCC
UK1 GU109335    TAGCGTCACATAATGAACATATACGTGCTCAGAATGATGGAGTGTTAGTAACTGGCGACCATACTCAGCTATTGGCTTTCGAGAATAATAATAAGACTCC
UK2 KJ437447    TAGCGTCACATAATGAACATATACGTGCTCAGAATGATGGAGTGTTAGTAACTGGCGACCATACTCAGCTGTTGGCTTTCGAGAATAATAATAAGACTCC
Korea1 JX875304 TAGCGTCACATAATGAACATATACGTGCTCAGAATGATGGAGTGTTAGTAACTGGCGATCATACTCAGCTATTGGCTTTCGAGAATAATAATAAAACTCC
Korea2 JX878305 TAGCGTCACATAATGAACATATGCGTGCTCAGAATGATGGAGTGTTAGTAACTGGCGACCATACTCAGCTATTGGGTTTTGAGAATAATAATAAAACTCC
China1 MF770715 TAGCGTCACATAATGAGCATATGCGTGCTCAGAATGATGGAGTGTTAGTAACTGGCGACCATACTCAGCTGTTGGCTTTCGAGAATAACAATAAAACTCC
China2 MF036686 TAGCGTCACATAATGAACATATGCGTGCTCAGAATGATGGAGTGTTAGTAACTGGCGACCATACTCAGCTGTTGGCTTTCGAGAATAATAATAAAACTCC
China3 MH165180 TAGCGTCACATAATGAACATATACGTGCTCAGAATGATGGAGTGTTAGTCACTGGCGACCATACTCAGCTGTTGGCTTTCGAGAATAATAATAAAACTCC
DWV-NVN         TCGCTTCACATAGTGAACACGCACGTGCTCAGAATGATGGAGTGTTAGTAACTGGCGACCATACTCAGTTGTTGGCTTTTGAGAATAACAATAAGACTCC
DWV-SVN         TCGCTTCACATAGTGAACACGCACGTGCTCAGAACGATGGAGTGTTAGTAACTGGCGACCATACTCAGTTGTTGGCTTTTGAGAATAACAATAAGACTCC
```

```
                         8010      8020      8030      8040      8050      8060      8070      8080      8090      8100
                    ....|....|....|....|....|....|....|....|....|....|....|....|....|....|....|....|....|....|....|....|
Japan AB070959      AATAAGTATCAACGCTGATGGTTTGTATGAGGTTATACTTCAAGGAGTATATACTTATCCATACCATGGCGATGGTGTTTGTGGTTCGATATTGTTGTCT
USA1 AY291384       AATAAGTATTAACGCTGATGGTTTGTATGAGGTTATACTTCAAGGAGTATATACCTATCCATACCACGGCGATGGTGTTTGTGGTTCGATATTGTTGTCT
Chile JQ413340      AATAAGTATCAATGCTGATGGTTTGTATGAGGTTATACTTCAAGGAGTATATACTTATCCATACCATGGCGATGGTGTTTGTGGTTCGATATTGTTATCT
Italy1 AJ489744     AATAAGTATCAACGCTGATGGTTTGTATGAGGTTATACTTCAAGGAGTATATACTTATCCATACCATGGCGATGGTGTTTGTGGTTCGATATTGCTGTCT
France KX373899     AATAAGTATCAACGCTGATGGTTTGTATGAGGTTATACTTCAAGGAGTATATACTTATCCATACCATGGCGATGGTGTTTGTGGTTCGATATTGTTGTCT
UK1 GU109335        AATAAGTATCAACGCTGATGGTTTGTATGAGGTTATACTTCAAGGAGTATATACTTATCCATACCATGGCGATGGTGTTTGTGGTTCGATATTGTTGTCT
UK2 KJ437447        AATAAGTATCAACGCTGATGGTTTGTATGAGGTTATACTTCAAGGAGTATATACTTATCCATACCATGGCGACGGTGTTTGTGGTTCGATATTGCTGTCT
Korea1 JX875304     AATAAGTATTAACGCTGATGGTTCGTATGAGGTTATACTTCAAGGAGTATATACTTATCCATACCATGGCGATGGTGTTTGTGGTCCGAAATTGTTATCT
Korea2 JX878305     AATAAGCATCAACGCTGATGGTTTGTATGAGGTTATACTTCAAGGAGTATATACTTACCCATACCATGGCGATGGTGTTTGTGGTTCGATATTGTTGTCT
China1 MF770715     GATAAGTATCAACGCTGATGGTTTGTATGAGGTTATACTTCAAGGGGTATATACTTATCCATACCATGGTGATGGTGTTTGTGGTTCGATATTGTTGTCT
China2 MF036686     AATAAGTATCAACGCTGATGGTTTGTATGAGGTTATACTTCAAGGAGTATATACTTATCCATACCATGGCGATGGTGTTTGTGGTTCGATATTGTTGTCT
China3 MH165180     AATAAGTATCAACGCTGATGGTTTGTATGAGGTTATACTTCAAGGAGTATATACTTATCCATACCATGGTGATGGTGTTTGTGGTTCGATATTGTTGTCT
DWV-NVN             AATAAGCATTAACGCTGATGGTTTGTATGAGGTTATACTTCAAGGAGTATATACTTATCCATATCATGGCGATGGTGTTTGTGGTTCGATATTGTTGTCT
DWV-SVN             AATAAGCATTAACGCTGACGGTTTGTATGAGGTTATACTTCAAGGAGTATATACTTATCCATACCATGGCGATGGTGTTTGTGGTTCGATATTGTTGTCT

                         8110      8120      8130      8140      8150      8160      8170      8180      8190      8200
                    ....|....|....|....|....|....|....|....|....|....|....|....|....|....|....|....|....|....|....|....|
Japan AB070959      CGGAATTTACAACGGCCGATTATAGGTATCCATGTTGCTGGTACTGAAGGATTGCATGGCTTTGGAGTTGCTGAACCACTTGTACATGAAATGTTCACTG
USA1 AY291384       CGGAATTTACAACGGCCAATTATAGGTATCCATGTTGCTGGTACTGAAGGATTGCATGGCTTTGGAGTTGCTGAACCACTTGTACATGAAATGTTCACCG
Chile JQ413340      CGGAATTTACAACGGCCAATTATAGGTATCCATGTTGCTGGTACTGAAGGATTGCATGGCTTTGGAGTTGCTGAACCACTTGTACATGAAATGTTCACTG
Italy1 AJ489744     CGGAATTTACAACGGCCAATTATAGGTATCCATGTTGCTGGTACTGAAGGATTGCATGGCTTTGGAGTYGCTGAACCAYTGGTACATGAAATGTTCACCG
France KX373899     CGGAATTTACAAAGGCCAATTATAGGTATCCATGTTGCTGGTACTGAAGGATTGCATGGCTTTGGAGTTGCTGAACCACTGGTACATGAAATGTTCACTG
UK1 GU109335        CGGAATTTACAACGGCCAATTATAGGTATCCATGTTGCTGGTACTGAAGGATTGCATGGCTTTGGAGTCGCTGAACCACTGGTACATGAAATGTTCACTG
UK2 KJ437447        CGGAATTTACAACGGCCGATTATAGGTATCCATGTTGCTGGTACTGAAGGATTGCATGGCTTTGGAGTTGCTGAACCACTGGTACATGAAATGTTCACTG
Korea1 JX875304     CGGAATTTACAACGGCCGATTATAGGTATCCATGTTGCTGGTACTGAAGGATTGCATGGCTTTGGAGTTGCTGAACCACTTGTACATGAAATGTTCACTG
Korea2 JX878305     CGGAATTTGCAACGGCCAATTATAGGTATCCATGTTGCTGGTCCTGAAGGATTGCATGGCTTTGGAGTTGCCGAACCACTTGTACATGAAATGTTCACTG
China1 MF770715     CGGAATTTACAGCGGCCGATTATAGGTATCCATGTTGCTGGTACTGAAGGATTGCATGGCTTTGGAGTTGCTGAACCACTTGTACATGAAATGTTCACTG
China2 MF036686     CGGAATTTACAGCGGCCGATTATAGGTATTCATGTTGCTGGTACTGAAGGATTGCATGGCTTTGGAGTTGCTGAACCACTTGTACATGAAATGTTCACCG
China3 MH165180     CGGAATTTACAACGGCCGATTATAGGTATCCATGTTGCTGGTACTGAAGGATTGCATGGCTTTGGAGTTGCTGAACCACTTGTACATGAAATGTTTACTG
DWV-NVN             CGAAATTTACAGCGACCGATTATAGGTATCCACGTTGCTGGCACTGAAGGATTGCATGGTTTTGGAGTTGCTGAACCGCTTGTACATGAGATGTTTACCG
DWV-SVN             CGAAATTTACAGCGACCGATTATAGGTATCCATGTTGCTGGCACTGAAGGATTGCATGGCTTTGGAGTTGCTGAACCGCTTGTACATGAGATGTTTACCG

                         8210      8220      8230      8240      8250      8260      8270      8280      8290      8300
                    ....|....|....|....|....|....|....|....|....|....|....|....|....|....|....|....|....|....|....|....|
Japan AB070959      GTAAAGCAATCGAGAGTGAAAGAGAGCCGTATGATCGTGTGTATGAACTTCCGTTGCGTGAATTAGATGAATCTGATATTGGTTTAGATACTGATTTATA
USA1 AY291384       GTAAAGCAATCGAGAGTGAAAGAGAGCCGTATGATCGTGTGTATGAACTTCCGTTGCGTGAATTAGATGAATCTGATATTGGTTTAGATACCGATTTATA
Chile JQ413340      GTAAAGCAATCGAGAGTGAAAGAGAGCCGTATGATCGTGTGTATGAACTTCCGTTGCGTGAATTAGATGAATCTGATATTGGTTTAGATACCGATTTATA
Italy1 AJ489744     GTAAAGCAATCGAGAGTGAAAGAGAGCCGTATGATCGTGTGTATGAACTTCCGTTGCGTGAATTAGATGAATCTGATATTGGTTTAGATACCGATTTATA
France KX373899     GTAAAGCAATTGAGAGTGAAAGAGAGCCGTATGATCGTGTGTATGAACTTCCGTTGCGCGAATTAGATGAATCTGATATTGGTTTAGATACTGATTTATA
UK1 GU109335        GTAAAGCAATCGAGAGTGAAAGAGAGCCGTATGATCGTGTGTATGAACTTCCGTTGCGTGAACTAGACGAATCTGATATTGGTTTAGATACCGATTTATA
UK2 KJ437447        GTAAAGCAATCGAGAGTGAAAGAGAGCCGTATGATCGTGTGTATGAACTTCCATTGCGTGAATTAGATGAATCCGATATTGGTTTAGATACCGACTTATA
Korea1 JX875304     GTAAAGCAATCGAGAGTGAAAGAGAGCCGTATGAACGTGTGTATGAACTCCCGTTGCGTGAATTAGATGAATCTGATATTGGTTTAGATACCGACTTATA
Korea2 JX878305     GTAAAGCAATCGAGAGTGAAAGAGAGCCGTATGACCGTGTGTATGAACTCCCGTTGCGTGAATTAGATGAATCTGATATTGGTTTAGATACCGATTTATA
China1 MF770715     GTAAAGCAATTGAGAGTGAAAGAGAGCCGTATGATCGTGTGTATGAACTTCCGTTGCGTGAATTAGATGAATCTGATATTGGTTTAGATACTGATTTATA
China2 MF036686     GTAAAGCAATCGAGAGTGAAAGAGAGCCGTATGATCGTGTGTATGAACTTCCGTTGCGTGAATTAGATGAATCTGATATTGGTTTAGATACTGATTTATA
China3 MH165180     GTAAAGCAATCGAGAGCGATAGAGAGCCGTATGATCGTGTGTACGAGCTTCCGTTGCGTGAATTAGATGAATCTGATATTGGTTTAGATACCGACTTATA
DWV-NVN             GTAAAGCAATTGAGAGTGAAAGAGAACCGTACGATCGTGTGTATGAGCTTCCATTGCGTGAATTAGATGAATCCGATATTGGTTTAGATACTGATCTATA
DWV-SVN             GTAAAGCAATTGAGAGTGAAAGAGAACCGTACGATCGTGTGTATGAGCTTCCGTTGCGTGAATTAGATGAATCTGACATTGGTTTAGATACTGATCTATA

                         8310      8320      8330      8340      8350      8360      8370      8380      8390      8400
                    ....|....|....|....|....|....|....|....|....|....|....|....|....|....|....|....|....|....|....|....|
Japan AB070959      TCCAATTGGTAGAGTGGATGCAAAGCTAGCTCATGCTCAAAGCCCTTCTACTGGGATTAAAAAGACGCTTATCCATGGAACATTTGATGTAAGGACTGAA
USA1 AY291384       TCCGATTGGTAGAGTGGATGCAAAGCTAGCTCATGCTCAAAGCCCTTCTACTGGGATTAAAAAGACGCTTATCCATGGAACATTTGATGTAAGGACTGAA
Chile JQ413340      TCCGATTGGTAGAGTGGATGCAAAGCTAGCTCATGCTCAAAGCCCTTCTACTGGGATTAAAAAGACGCTTATCCATGGAACATTTGATGTAAGGACTGAA
Italy1 AJ489744     TCCGATTGGTAGAGTGGATGCAAAGTTAGCTCATGCTCAAAGCCCTTCTACTGGGATCAAAAAGACGCTTATCCATGGAACATTTGATGTAAGGACTGAA
France KX373899     TCCGATTGGTAGAGTGGACGCAAAGTTGGCTCATGCTCAAAGCCCTTCTACTGGGATCAAAAAGACGCTTATCCATGGAACATTTGATGTAAGGACTGAA
UK1 GU109335        TCCGATTGGTAGAGTGGATGCAAAATTAGCTCATGCTCAAAGCCCTTCTACTGGGATCAAAAAGACGCTTATCCATGGAACATTTGATGTAAGGACTGAA
UK2 KJ437447        TCCGATTGGTAGAGTGGATGCAAAGTTAGCTCATGCTCAAAGCCCTTCTACTGGGATCAAAAAGACGCTTATCCATGGAACATTTGATGTAAGGACTGAA
Korea1 JX875304     TCCGATTGGTAAAGTGGATGCAAAGCTAGCTCATGCTCAAAGCCCTTCTACTGGGATTAAAAAGACGCTTATCCATGGAACATTTGATGTAAGGACTGAA
Korea2 JX878305     TCCGATTGGTAAAGTGGATGCAAAGCTAGCTCATGCTCAAAGCCCTTCTACTGGGATTAAAAAGACGCTTATCCATGGAACATTTGATGTAAGGACTGAA
China1 MF770715     TCCGATTGGTAGAGTGGATGCAAAGCTAGCTCATGCTCAAAGCCCTTCTACTGGGATTAAAAAGACGCTTATCCATGGAACATTTGATGTAAGGACTGAA
China2 MF036686     TCCAATTGGTAGAGTGGATGCAAAGCTAGCTCATGCTCAAAGCCCTTCTACTGGGATTAAAAAGACGCTTATCCATGGAACATTTGATGTAAGGACTGAA
China3 MH165180     TCCGATTGGTAAAGTGGATGCAAAGCTAGCTCATGCTCAAAGCCCTTCTACTGGGATTAAAAAGACGCTTATCCATGGAACATTTGATGTAAGGACTGAA
DWV-NVN             TCCGATTGGTAAGGTGGATGCAAAGCTAGCTCATGCTCAAAGTCCTTCTACTGGGATTAAAAAGACGCTTATTCATGGAACGTTTGATGTAAGGACTGAA
DWV-SVN             TCCGATTGGTAAGGTGGATGCAAAGTTAGCTCATGCTCAAAGTCCTTCTACTGGGATTAAAAAGACGCTTATTCATGGAACGTTTGATGTAAGGACTGAA
```

```
                        8410      8420      8430      8440      8450      8460      8470      8480      8490      8500
                    ....|....|....|....|....|....|....|....|....|....|....|....|....|....|....|....|....|....|....|....|
Japan AB070959      CCAAATCCGATGTCATCACGTGATCCAAGAAYAGCACCGCACGATCCTTTGAAGTTAGGGTGTGAAAAGCATGGCATGCCCTGTTCACCGTTTAATAGGA
USA1 AY291384       CCAAATCCGATGTCATCACGTGATCCAAGAATAGCGCCGCATGATCCTTTGAAGTTAGGGTGCGAAAAGCATGGCATGCCTTGTTCACCGTTTAATAGGA
Chile JQ413340      CCAAACCCGATGTCATCACGTGATCCAAGAATAGCGCCGCACGATCCTTTGAAGTTAGGGTGTGAAAAGCATGGCATGCCCTGTTCACCGTTTAATAGGA
Italy1 AJ489744     CCAAATCCGATGTCGTCACGTGATCCAAGAATAGCGCCGCATGATCCTTTGAAGTTAGGGTGTGAAAAGCATGGTATGCCTTGTTCACCGTTTAATAGGA
France KX373899     CCAAATCCGATGTCGTCACGTGATCCAAGAATAGCGCCGCATGATCCTTTGAAATTAGGGTGTGAAAAGCATGGTATGCCTTGTTCACCGTTTAATAGGA
UK1 GU109335        CCAAATCCGATGTCGTCACGTGATCCAAGAATAGCGCCGCATGATCCTTTGAAGTTAGGGTGTGAAAAGCATGGTATGCCTTGTTCACCGTTTAATAGGA
UK2 KJ437447        CCAAATCCGATGTCGTCACGTGATCCAAGAATAGCGCCGCATGATCCTTTGAAGTTAGGGTGTGAAAAGCATGGTATGCCTTGTTCACCGTTTAATAGGA
Korea1 JX875304     CCAAATCCGATGTCATCACGTGATCCAAGAATAGCGCCGCATGATCCTTTGAAGTTAGGGTGTGAAAAGCATGGCATGCCTTGCTCACCGTTTAATAGGA
Korea2 JX878305     CCAAATCCGATGTCATCACGTGATCCAAGAATAGCGCCGCAAGATCCTTTGAAGTTAGGGTGTGAAAAGCATGGCATGCCTTGCTCACCGTTTAATAGGA
China1 MF770715     CCAAATCCGATGTCATCACGTGATCCAAGAATAGCGCCGCACGATCCTTTGAAGTTAGGGTGTGAAAAGCACGGCATGCCTTGTTCGCCGTTTAATAGGA
China2 MF036686     CCAAATCCGATGTCATCACGTGATCCAAGAATAGCACCGCATGATCCCTTGAAGTTAGGGTGTGAAAAGCATGGTATGCCCTGTTCACCGTTTAATAGGA
China3 MH165180     CCAAATCCGATGTCATCACGTGATCCAAGAATAGCGCCGCATGATCCTTTGAAGTTAGGATGTGAAAAGCATGGCATGCCTTGTTCACCGTTTAATAGGA
DWV-NVN             CCAAATCCTATGTCATCACGTGATCCAAGGATAGCGCCGCATGATCCTTTGAAGTTAGGGTGTGAAAAGCATGGTATGCCTTGTTCACCATTTAATAGGA
DWV-SVN             CCAAATCCTATGTCATCACGTGATCCACGGATAGCGCCACATGATCCTTTGAAGTTAGGGTGTGAAAAGCATGGTATGCCTTGTTCACCATTTAATAGGA

                        8510      8520      8530      8540      8550      8560      8570      8580      8590      8600
                    ....|....|....|....|....|....|....|....|....|....|....|....|....|....|....|....|....|....|....|....|
Japan AB070959      AACATCTGGAATTAGCGACAAATCATTTGAAAGAAAAATTAGTTTCAGTAGTTAAACCAATAAATGGTTGCAAGATTAGAAGTTTGCAAGATGCTGTATG
USA1 AY291384       AACATCTGGAATTAGCGACAAATCATTTGAAAGAAAAATTAGTTTCAGTAGTTAAACCAATAAATGGTTGCAAGATTAGAAGTTTGCAAGATGCTGTATG
Chile JQ413340      AACATCTGGAATTAGCGACAAATCATTTGAAAGAAAAATTAGTTTCAGTAGTTAAACCAATAAATGGTTGCAAGATTAGAAGTTTGCAAGATGCTGTATG
Italy1 AJ489744     AACATCTGGAATTAGCGACAAATCATTTGAAAGAAAAATTAGTTTCAGTAGTTAAACCAATAAATGGTTGCAAGATTAGAAGTTTGCAAGATGCTRTATG
France KX373899     AACATCTGGAATTAGCGACAAATCATTTGAAAGAAAAATTAGTTTCAGTAGTTAAACCAATAAATGGTTGCAAGATTAGAAGTTTGCAAGATGCTGTATG
UK1 GU109335        AACATCTGGAATTAGCGACAAATCATTTGAAAGAAAAATTAATTTCAGTAGTTAAACCAATAAATGGTTGCAAGATTAGAAGTTTGCAAGATGCTGTATG
UK2 KJ437447        AACATCTGGAATTAGCGACAAATCATTTGAAAGAAAAATTAATTTCAGTAGTTAAACCAATAAATGGTTGCAAGATTAGAAGTTTGCAAGATGCTGTATG
Korea1 JX875304     AACATCTGGAATTAGCGACAAATCATTTGAAAGAAAAATTGGTTTCAGTAGTTAAACCAATAAATGGTTGCAAGATTAGAAGTTTGCAAGATGCTGTATG
Korea2 JX878305     AACATCTGGAATTAGCGACAAATCATTTGAAAGAAAAATTAATTTCAGTAGTTAAACCAATAAATGGTTGCAAGATTAGAAGTTTGCAAGATGCTGTATG
China1 MF770715     AACATCTGGAATTAGCGACAAATCATTTGAAAGAAAAATTAGTTTCAGTAGTTAAACCAATAAATGGTTGCAAGATTAGAAGTTTGCAAGATGCTGTATG
China2 MF036686     AACATCTGGAATTAGCGACAAATCATTTGAAAGAAAAATTAATTTCAGTAGTTAAACCAATAAATGGTTGCAAGATTAGAAGTTTGCAAGATGCTGTATG
China3 MH165180     AACATCTGGAATTAGCGACAAATCATTTGAAAGAAAAATTAGTTTCGGTAGTTAAACCAATAAATGGTTGCAAGATTAGAAGTTTGCAAGACGCTGTATG
DWV-NVN             AACATTTGGAATTAGCGACAAATCACCTGAAAGAAAAGTTAGTTTCAGTAGTTAAACCAATAAATGGCTGTAAGATTAGAAGTTTGCAAGATGCTGTGTG
DWV-SVN             AACATTTGGAATTAGCGACAAATCACCTGAAAGAAAAGTTAGTTTCAGTAGTTAAACCAATAAATGGCTGTAAGATTAGAAGTTTGCAAGATGCTGTATG

                        8610      8620      8630      8640      8650      8660      8670      8680      8690      8700
                    ....|....|....|....|....|....|....|....|....|....|....|....|....|....|....|....|....|....|....|....|
Japan AB070959      TGGTGTGCCTGGTTTAGATGGGTTTGATTCAATATCTTGGAATACTAGTGCTGGTTTTCCTTTGTCTTCATTAAAGCCACCTGGAGCATCAGGTAAGCGA
USA1 AY291384       TGGTGTGCCTGGATTAGATGGGTTTGATTCGATATCTTGGAATACTAGTGCTGGTTTTCCTTTGTCTTCATTAAAGCCACCTGGAACATCGGGTAAGCGA
Chile JQ413340      TGGTGTGCCTGGATTAGATGGGTTTGATTCGATATCTTGGAATACTAGTGCTGGTTTTCCTTTATCTTCATTAAAGCCACCTGGAACATCAGGTAAGCGA
Italy1 AJ489744     TGGTGTGCCTGGTTTAGATGGGTTTGATTCGATATCTTGGAATACTAGTGCTGGTTTTCCTTTGTCTTCATTAAAGCCACCTGGAACATCAGGYAAGCGA
France KX373899     TGGTGTGCCTGGTTTAGATGGGTTTGATTCGATATCTTGGAATACTAGTGCTGGTTTTCCTTTGTCTTCATTAAAGCCACCTGGAACATCAGGTAAGCGA
UK1 GU109335        TGGTGTGCCTGGTGTAGATGGGTTTGATTCGATATCTTGGAATACTAGTGCTGGTTTTCCTTTGTCTTCATTAAAGCCACCTGGAACATCAGGTAAGCGA
UK2 KJ437447        TGGTGTGCCTGGTGTAGATGGGTTTGATTCGATATCTTGGAATACTAGTGCTGGTTTTCCTTTGTCTTCATTAAAGCCACCTGGAACATCAGGTAAGCGA
Korea1 JX875304     TGGGGTGCCTGGTTTAGATGGGTTTGATTCGATATCTTGGAATACTAGTGCTGGTTTTCCTTTGTCTTCATTAAAGCCGCCTGGAACATCAGGTAAGCGA
Korea2 JX878305     TGGTGTGCCTGGTTTAGATGGGTTTGATTCGATATCTTGGAATACTAGTGCTGGTTTTCCTTTGTCTTCATTAAAGCCACCTGGAACTTCAGGTAAGCGA
China1 MF770715     TGGTGTGCCTGGATTAGATGGGTTTGATTCGATATCCTGGAATACTAGTGCTGGTTTTCCTTTGTCTTCATTAAAGCCACCTGGAACATCAGGCAAGCGA
China2 MF036686     TGGTGTGCCTGGTTTAGATGGGTTTGATTCAATTTCTTGGAATACTAGTGCTGGTTTTCCTTTGTCTTCATTGAAGCCACCTGGAACATCGGGTAAGCGA
China3 MH165180     TGGTGTGCCTGGTTTAGATGGGTTTGATTCGATATCTTGGAATACTAGTGCTGGTTTTCCTTTGTCTTCATTAAAGCCACCTGGAACATCAGGTAAACGA
DWV-NVN             TGGTGTGCCTGGAGTAGATGGGTTTGACTCGATATCTTGGAATACTAGTGCTGGTTTTCCTTTGTCTTCATTAAAGCCACCTGGAACATCAGGTAAGCGG
DWV-SVN             TGGTGTGCCTGGTGTAGATGGGTTTGACTCGATATCTTGGAATACTAGTGCTGGTTTTCCTTTGTCTTCATTAAAGCCACCTGGAACATCAGGTAAGCGG

                        8710      8720      8730      8740      8750      8760      8770      8780      8790      8800
                    ....|....|....|....|....|....|....|....|....|....|....|....|....|....|....|....|....|....|....|....|
Japan AB070959      TGGTTGTTTGACATTGAGCTACAAGACTCGGGATGTTATCTCTTGCGTGGAATGCGTCCCGAACTTGAGATTCAATTATCAACGACACAGTTAATGAGGA
USA1 AY291384       TGGTTGTTTGACATTGAGCTACAAGACTCGGGATGTTATCTCCTGCGTGGAATGCGTCCCGAACTTGAGATTCAATTATCAACGACACAGTTAATGAGGA
Chile JQ413340      TGGTTGTTTGACATTGAGCTACAAGATTCGGGATGTTATCTCCTGCGTGGAATGCGTCCCGAACTTGAGATTCAATTATCAACGACACAGTTAATGAGGA
Italy1 AJ489744     TGGTTGTTTGAYATTGAGCTACAAGAYTCGGGATGTTATCTCYTGCGTGGAATGCGTCCCGAACTTGAGATTCAATTATCAACGACACAGTTAATGAGGA
France KX373899     TGGTTGTTTGATATTGAGCTACAAGACTCGGGATGTTATCTTTTGCGAGGAATGCGTCCCGAACTCGAGATTCAATTATCAACGACACAGTTAATGAGGA
UK1 GU109335        TGGTTGTTTGACATTGAGCTACAAGACTCGGGATGTTATCTCTTGCGTGGAATGCGTCCCGAACTTGAGATTCAATTATCAACGACACAGTTAATGAGGA
UK2 KJ437447        TGGTTGTTTGACATTGAGCTACAAGACTCGGGATGTTATCTCTTGCGTGGAATGCGTCCCGAACTTGAGATTCAATTATCAACGACACAGTTAATGAGGA
Korea1 JX875304     TGGTTGTTTGACATTGAGCTACAAGACTCGGGATGTTATCTCTTGCGTGGAATGCCCCCCGAACTTGAGATTCAGCTGTCGACAACCCAGTTAATGAGGA
Korea2 JX878305     TGGTTGTTTGACATTGAGCTACAAGACTCGGGATGTTATCTCTTGCGTGGAATGCGTCCCGATCTTGAGATTCAATTATCAACGACACAGTTAATGAGGA
China1 MF770715     TGGTTGTTCGACATTGAGCTACAAGACTCGGGATGTTATCTCTTGCGTGGCATGCGTCCCGAACTTGAGATTCAATTATCAACGACACAGTTAATGAGGA
China2 MF036686     TGGTTGTTTGACATTGAGTTACAAGATTCGGGATGTTATCTTTTGCGTGGAATGCGTCCCGAACTTGAGATTCAATTATCAACAACACAGTTAATGAGGA
China3 MH165180     TGGCTGTTTGATATTGAGCTACAAGATTCGGGATGTTATCTCTTGCGTGGAATGCGTCCCGAACTTGAGATTCAATTATCGACGACACAGTTAATGAGGA
DWV-NVN             TGGTTGTTCGATATTGAGCTTCAAGATTCAGGATGTTATCTATTGCGTGGAATGCGTCCTGAACTTGAGATTCAATTGTCGACAACCCAGTTAATGAGGA
DWV-SVN             TGGTTGTTCGATATTGAGCTTCAAGATTCGGGATGTTATCTATTGCGTGGAATGCGTCCTGAACTTGAGATTCAACTGTCGACGACCCAGTTAATGAGGA
```

```
                         8810      8820      8830      8840      8850      8860      8870      8880      8890      8900
                    ....|....|....|....|....|....|....|....|....|....|....|....|....|....|....|....|....|....|....|....|
Japan AB070959      AAAAGGGAATAAAACCTCACACTATATTCACGGATTGTTTGAAAGATACTTGTTTGCCTGTAGAAAAATGTAGAATACCTGGTAAGACTAGAATATTTAG
USA1 AY291384       AAAAGGGAATAAAACCTCACACTATATTCACGGATTGTTTGAAGGATACTTGTTTGCCTGTTGAAAAATGTAGAATACCTGGTAAGACTAGAATATTTAG
Chile JQ413340      AAAAGGGAATAAAACCTCACACTATATTCACGGATTGTTTGAAAGATACTTGTTTGCCTGTGGAAAAATGTAGAATACCTGGTAAGACTAGAATATTTAG
Italy1 AJ489744     AAAAGGGAATAAAACCTCACACTATATTCACGGATTGTTTGAAAGATACTTGTTTGCCTGTTGAAAAATGTAGAATACCTGGTAAGACTAGAATATTTAG
France KX373899     AAAAAGGAATAAAACCTCACACTATATTCACGGATTGTTTGAAAGATACTTGTTTGCCTGTTGAAAAATGTAGAATACCTGGTAAGACTAGAATATTTAG
UK1 GU109335        AAAAGGGAATAAAACCTCACACTATATTCACGGATTGTTTGAAAGATACCTGTTTGCCTGTGGAAAAATGTAGAATACCTGGTAAGACTAGAATATTTAG
UK2 KJ437447        AAAAGGGAATAAAACCTCACACTATATTCACGGATTGTTTGAAAGATACCTGTTTGCCTGTGGAAAAATGTAGAATACCTGGTAAGACTAGAATATTTAG
Korea1 JX875304     AAAAGGGAATAAAACCTCACACTATATTCACGGATTGTTTGAAAGATACTTGTTTGCCTGTAGAAAAATGTAGAATACCTGGTAAGACTAGAATATTTAG
Korea2 JX878305     AAAAGGGAATAAAACCTCACACTATATTCACGGATTGCTTGAAAGATACTTGTTTGCCTGTGGAAAAATGTAGAATACCTGGTAAGACTAGAATATTTAG
China1 MF770715     AAAAGGGAATAAAACCTCACACTATATTCACGGATTGTTTGAAAGATACTTGTTTGCCTGTGGAAAAATGTAGAATACCTGGTAAGACTAGAATATTTAG
China2 MF036686     AAAAGGGAATAAAACCTCACACTATATTCACGGATTGTTTGAAAGATACTTGTTTGCCTGTGGAAAAATGTAGAATACCTGGTAAGACTAGAATATTTAG
China3 MH165180     AAAAGGGAATAAAACCCCACACTATATTCACGGATTGTTTGAAAGATACTTGTTTGCCTGTGGAAAAATGTAGAATACCTGGTAAGACTAGAATATTTAG
DWV-NVN             AAAAGGGAATAAAACCCCACACTATATTCACGGATTGTCTGAAAGATACTTGTTTGCCTGTCGAAAAATGTAGAATACCTGGTAAGACTAGAATATTTAG
DWV-SVN             AAAAAGGAATAAAACCTCACACTATATTTACGGATTGCCTGAAAGATACTTGTTTGCCTGTCGAAAAATGTAGAATACCTGGTAAGACTAGAATATTTAG

                         8910      8920      8930      8940      8950      8960      8970      8980      8990      9000
                    ....|....|....|....|....|....|....|....|....|....|....|....|....|....|....|....|....|....|....|....|
Japan AB070959      TATAAGTCCAGTACAGTTTACTATACCGTTTAGACAGTATTATTTAGATTTTATGGCATCCTATCGAGCTGCACGCCTTAATGCTGAGCATGGTATAGGT
USA1 AY291384       TATAAGTCCGGTACAGTTTACCATACCGTTTCGACAGTATTACTTAGACTTTATGGCATCCTATCGAGCTGCACGACTTAATGCTGAGCATGGTATTGGT
Chile JQ413340      TATAAGTCCGGTACAGTTTACCATACCGTTTCGACAGTATTATTTAGACTTTATGGCATCCTATCGAGCTGCACGACTTAATGCTGAGCATGGTATTGGT
Italy1 AJ489744     TATAAGTCCGGTACAGTTTACCATACCGTTTCGACAGTATTACTTAGACTTTATGGCATCCTATCGAGCTGCACGACTTAATGCTGAGCATGGTATTGGT
France KX373899     TATAAGTCCGGTACAGTTTACCATACCGTTTCGACAGTATTACTTAGATTTTATGGCATCCTATCGAGCTGCACGACTTAATGCTGAGCATGGTATTGGT
UK1 GU109335        TATAAGTCCGGTACAGTTTACTATACCTTTTAGACAGTATTACTTAGATTTTATGGCATCCTATCGAGCTGCACGACTTAATGCTGAGCATGGTATTGGT
UK2 KJ437447        TATAAGTCCGGTACAGTTTACTATACCTTTTAGACAGTATTACTTAGATTTTATGGCATCCTATCGAGCTGCACGACTTAATGCTGAGCATGGTATTGGT
Korea1 JX875304     TATAAGTCCGGTACAGTTTACTATACCGTTTAGACAGTATTACTTAGATTTTATGGCATCCTATCGAGCTGCGCGACTTAATGCTGAGCATGGTATTGGT
Korea2 JX878305     TATAAGTCCGGTACAGTTCACTATACCGTTTAGACAGTATTACTTAGATTTTATGGCATCCTATCGAGCTGCACGACTTAATGCTGAGCATGGTATTGGT
China1 MF770715     TATAAGTCCTGTACAGTTTACTATACCGTTTAGACAGTATTACTTAGATTTTATGGCATCCTATCGAGCTGCACGTCTTAATGCTGAGCATGGTATTGGT
China2 MF036686     TATAAGTCCGGTACAGTTTACTATACCGTTTAGACAGTATTACTTAGATTTTATGGCATCCTATCGAGCTGCACGACTAAATGCTGAGCACGGTATTGGT
China3 MH165180     CATAAGTCCGGTACAATTTACTATACCGTTTAGACAGTATTACTTAGATTTTATGGCATCCTATCGAGCTGCACGACTTAATGCTGAGCATGGTATTGGT
DWV-NVN             TATAAGTCCTGTACAGTTTACTATACCGTTTCGACAGTATTACTTAGATTTTATGGCATCCTATCGAGCTGCGCGGCTCAATGCTGAGCATGGTATTGGT
DWV-SVN             TATAAGTCCTGTACAGTTTACTATACCGTTTCGACAGTATTACTTAGATTTTATGGCATCCTATCGAGCTGCGCGGCTCAATGCTGAGCATGGTATTGGT

                         9010      9020      9030      9040      9050      9060      9070      9080      9090      9100
                    ....|....|....|....|....|....|....|....|....|....|....|....|....|....|....|....|....|....|....|....|
Japan AB070959      ATTGATGTTAATAGCTTAGAATGGACAAATTTGGCAACAAGTTTGTCAAAGTATGGCACTCATATCGTGACGGGTGACTATAAGAATTTTGGCCCTGGAT
USA1 AY291384       ATTGATGTTAACAGCTTAGAGTGGACAAATTTGGCAACAAGGTTGTCTAAGTATGGCACTCACATCGTGACAGGAGACTATAAGAATTTTGGTCCTGGGT
Chile JQ413340      ATTGATGTTAACAGCTTAGAGTGGACAACTTTGGCAACAAGGTTGTCTAAGTATGGCACTCACATCGTGACAGGAGACTATAAGAATTTTGGTCCTGGGT
Italy1 AJ489744     ATTGATGTTAACAGCTTAGAGTGGACAAATTTGGCAACAAGGTTGTCTAAGTWTGGCACTCACATCGTGACAGGAGATTATAAGAATTTTGGTCCTGGGT
France KX373899     ATTGATGTTAACAGCTTAGAGTGGACAAATTTGGCAACAAGGTTGTCTAAGTATGGCACTCATATCGTGACAGGAGACTACAAGAATTTTGGTCCTGGGT
UK1 GU109335        ATTGATGTTAACAGCTTAGAATGGACAAATTTGGCAACAAGTCTGTCAAAGTATGGCACTCATATCGTGACGGGTGACTATAAGAATTTTGGTCCTGGGT
UK2 KJ437447        ATTGATGTTAACAGCTTAGAATGGACAAATTTGGCAACAAGTCTGTCAAAGTATGGCACTCATATCGTGACGGGTGACTATAAGAATTTTGGTCCTGGAT
Korea1 JX875304     ATTGATGTTAACAGCTTAGAATGGACAAATTTGGCAACAAGTTTGTCAAAGTATGGCACTCATATCGTGACGGGTGACTATAAGAATTTTGGCCCTGGAT
Korea2 JX878305     ATTGATGTTAACAGCTTAGAATGGACAAATTTGGCAACAAGTTTGTCAAAGTACGGTACTCATATCGTGACGGGTGACTATAAGAATTTTGGTCCTGGAT
China1 MF770715     ATTGATGTTAACAGCTTAGAATGGACAAATTTGGCAACAAGTTTGTCAAAGTATGGCACTCATATCGTGACAGGTGACTATAAGAATTTTGGCCCTGGAT
China2 MF036686     ATTGATGTTAACAGCTTAGAGTGGACAAATTTGGCAACAAGTTTGTCTAAGTATGGCACTCACATCGTGACGGGTGACTATAAGAATTTTGGCCCTGGAT
China3 MH165180     ATTGATGTTAACAGCTTAGAATGGACAAATTTGGCAACAAGTTTGTCAAAGTATGGCACTCATATCGTGACGGGTGACTATAAGAACTTTGGCCCTGGAT
DWV-NVN             ATTGATGTTAACAGCTTAGAATGGACAAATTTGGCGACATGTTTGTCCAAGTATGGCACTCATATCGTGACAGGTGACTATAAGAATTTTGGTCCTGGAT
DWV-SVN             ATTGATGTTAACAGCTTAGAATGGACAAATTTGGCGACATGTTTGTCCAAGTATGGCACTCATATTGTGACGGGTGACTATAAGAATTTTGGTCCTGGAT

                         9110      9120      9130      9140      9150      9160      9170      9180      9190      9200
                    ....|....|....|....|....|....|....|....|....|....|....|....|....|....|....|....|....|....|....|....|
Japan AB070959      TAGATTCCGATGTTGCAGCTTCGGCGTTCGAAATTATTATCGATTGGGTATTACATTATACTGAAGAAGATAATAAAGACGAAATGAAGCGAGTAATGTG
USA1 AY291384       TAGATTCCGATGTTGCAGCTTCAGCGTTCGAAATTATTATCGACTGGGTATTACATTACACCGAAGAAGATAATAAAGACGAAATGAAGCGAGTAATGTG
Chile JQ413340      TAGATTCCGATGTTGCAGCTTCAGCGTTCGAAATTATTATCGACTGGGTATTACATTACACCGAAGAAGATAATAAAGACGAAATGAAGCGAGTAATGTG
Italy1 AJ489744     TAGATTCCGATGTTGCAGCTTCAGCGTTCGAAATTATTATCGACTGGGTATTACATTACACCGAAGAAGATAATAAAGAYGAAATGAAGCGAGTAATGTG
France KX373899     TAGATTCCGATGTTGCAGCTTCAGCGTTTGAAATTATTATCGACTGGGTATTACATTATACCGAAGAAGATAATAAAGATGAAATGAAGCGAGTAATGTG
UK1 GU109335        TAGATTCCGATGTTGCAGCTTCAGCGTTTGAAATTATTATCGACTGGGTATTACATTATACTGAAGAAGATAATAAAGACGAAATGAAGCGAGTAATGTG
UK2 KJ437447        TAGATTCCGATGTTGCAGCTTCAGCGTTTGAAATTATTATCGACTGGGTATTACATTATACTGAAGAAGATAATAAAGACGAAATGAAGCGAGTAATGTG
Korea1 JX875304     TAGATTCTGACGTTGCAGCTTCGGCGTTCGAAATTATTATCGACTGGGTATTACATTATACTGAAGAAGATAATAAAGACGAAATGAAGCGAGTAATGTG
Korea2 JX878305     TAGATTCTGATGTTGCAGCTTCGGCGTTCAAAATTATTATCGACTGGGTATTACATTATACTGAAGAAGATAATAAAGACGAAATGAAGCGAGTAATGTG
China1 MF770715     TAGATTCCGATGTTGCAGCTTCGGCGTTCGAAATTATTATCGACTGGGTATTGCATTATACTGAAGAAGATAATAAAGACGAAATGAAGCGAGTAATGTG
China2 MF036686     TAGATTCCGACGTTGCAGCTTCGGCGTTTGAAATTATTATCGACTGGGTATTACATTATACCGAAGAAGATAATAAAGACGAAATGAAGCGAGTAATGTG
China3 MH165180     TAGATTCTGATGTTGCAGCTTCGGCGTTCGAAATTATTATCGACTGGGTGCTACATTACACCGAAGAAGATAATAAAGACGAAATGAAGCGAGTAATGTG
DWV-NVN             TAGATTCTGATGTTGCAGCCTCAGCGTTCGAAATTATTATTGACTGGGTATTACATTATACTGAAGAAGATAATAAAGACGAAATGAAGCGAGTGATGTG
DWV-SVN             TAGATTCCGATGTTGCAGCCTCAGCCTTCGAAATTATTATTGACTGGGTATTACATTATACTGAAGAAGATAATAAAGACGAAATGAAGCGAGTGATGTG
```

```
                          9210      9220      9230      9240      9250      9260      9270      9280      9290      9300
                  ....|....|....|....|....|....|....|....|....|....|....|....|....|....|....|....|....|....|....|....|
Japan AB070959    GACCATGGCGCAAGAGATTTTAGCGCCTAGTCATCTATGTCGTGATTTAGTGTACCGGGTACCTTGTGGAATTCCATCAGGTTCTCCGATAACGGACATT
USA1 AY291384     GACCATGGCGCAAGAGATCTTAGCGCCTAGTCATCTATATCGCGACTTGGTGTACCGAGTACCTTGCGGAATTCCATCAGGTTCTCCAATAACGGACATA
Chile JQ413340    GACCATGGCGCAAGAGATCTTAGCGCCTAGTCATCTATATCGTGACTTGGTGTACCGAGTACCTTGCGGAATTCCATCAGGTTCTCCAATAACGGACATA
Italy1 AJ489744   GACCATGGCGCAAGAGATCTTAGCGCCTAGTCATCTATATCGCGACYTGGTGTACCGAGTACCTTGTGGAATTCCATCAGGTTCTCCAATAACGGACATA
France KX373899   GACCATGGCGCAAGAGATTTTAGCGCCTAGTCATCTATGTCGCGACTTGGTGTACCGAGTACCTTGTGGAATTCCATCAGGTTCTCCAATAACGGACATA
UK1 GU109335      GACCATGGCGCAGGAGATTTTAGCGCCTAGTCATTTATGTCGTGATTTAGTGTACCGAGTACCTTGTGGAATTCCATCAGGTTCTCCGATAACGGACATT
UK2 KJ437447      GACCATGGCGCAGGAGATTTTAGCGCCTAGTCATCTATGTCGTGATTTAGTGTACCGAGTACCTTGTGGAATTCCATCAGGTTCTCCGATAACGGACATT
Korea1 JX875304   GACCGTGGCGCAGGAGATCTTAGCGCCTAGTCATCTATGTCGTGATTTAGTGTACCGAGTACCTTGTGGAATTCCATCAGGTTCTCCAATAACGGACATT
Korea2 JX878305   GACCATGGCGCAAGAGATCTTAGCGCCTAGTCATCTATGTCGCGATTTGGTGTACCGAGTACCTTGTGGAATTCCATCAGGTTCTCCAATAACGGACATA
China1 MF770715   GACCATGGCGCAAGAGATCTTAGCGCCTAGTCACCTATGTCGCGATTTGGTGTACCGAGTACCTTGTGGAATTCCATCAGGTTCTCCAATAACGGACATT
China2 MF036686   GACCATGGCGCAAGAGATCTTAGCGCCTAGTCATCTATGTCGCGATTTGGTGTACCGAGTACCTTGTGGAATTCCATCAGGTTCTCCAATAACGGACATA
China3 MH165180   GACCATGGCGCAAGAGATCTTAGCGCCTAGTCATCTATGTCGCGATTTGGTGTACCGAGTACCTTGTGGAATTCCATCAGGTTCACCGATAACGGACATT
DWV-NVN           GACCATGGCGCAAGAGATCTTAGCGCCTAGTCATCTATGTCGAGATTTGGTGTACCGAGTACCTTGTGGAATTCCATCAGGTTCTCCGATAACGGACATA
DWV-SVN           GACCATGGCGCAAGAGATCTTAGCGCCTAGTCATCTATGTCGAGATTTGGTGTACCGAGTACCTTGTGGGATTCCATCAGGTTCTCCAATAACAGACATA

                          9310      9320      9330      9340      9350      9360      9370      9380      9390      9400
                  ....|....|....|....|....|....|....|....|....|....|....|....|....|....|....|....|....|....|....|
Japan AB070959    TTAAATACAATTTCAAATTGTCTGTTAATTAGGTTAGCTTGGTTAGGTATTACTGATTTGCCTTTATCCGAGTTCTCTCAAAATGTTGTTCTTGTTTGTT
USA1 AY291384     TTGAATACAATTTCAAATTGTTTGTTAATTAGGTTAGCTTGGTTAGGTATTACTGATTTGCCTTTGTCCGAGTTCTCTCAAAATGTTGTTCTTGTCTGTT
Chile JQ413340    TTGAATACAATTTCAAATTGTTTGTTAATTAGGTTAGCTTGGTTAGGTATTACTGATTTGCCTTTGTCCGAGTTCTCTCAAAATGTTGTTCTTGTCTGTT
Italy1 AJ489744   TTGAAYACAATTTCAAATTGTTTGTTAATTAGGTTAGCTTGGTTAGGTATTACTGACTTGCCTTTGTCCGAGTTCTCTCAAAATGTTGTTCTTGTCTGTT
France KX373899   TTGAACACAATTTCAAATTGTTTGTTAATTAGGTTAGCTTGGTTAGGTATTACTGACTTGCCTTTGTCCGAGTTCTCTCAAAATGTTGTTCTTGTCTGTT
UK1 GU109335      TTGAATACAATTTCGAATTGTCTGTTGATTAGGTTAGCTTGGTTAGGTATTACTGATTTGCCTTTATCCGAGTTCTCTCAAAATGTTGTTCTTGTTTGTT
UK2 KJ437447      TTGAATACAATTTCGAATTGTCTGTTGATTAGGTTAGCTTGGTTAGGTATTACTGATTTGCCTTTATCCGAGTTCTCTCAAAATGTTGTTCTTGTTTGTT
Korea1 JX875304   TTGAATACAATTTCAAATTGTCTGTTAATTAGGTTAGCTTGGTTAGGTATTACTGATTTGCCTTTATCCGAGTTCTCTCAAAATGTTGTTCTTGTTTGTT
Korea2 JX878305   TTGAACACAATTTCAAATTGTTTGTTAATTAGGTTAGCTTGGTTAGGTATTACTGACTTGCCTTTGTCCGAGTTCTCTCAAAATGTTGTTCTTGTTTGTT
China1 MF770715   TTGAATACAATTTCAAATGGTCTGTTAATTAGGTTAGCTTGGTTAGGTATTACTGATTTGCCTTTATCCGAGTTTTCTCAAAATGTTGTTCTTGTTTGTT
China2 MF036686   TTGAATACAATTTCAAATTGTCTGTTAATTAGGTTGGCTTGGTTAGGTATTACTAATTTGCCTTTATCCGAGTTTTCTCAAAATGTTGTTCTTATTTGTT
China3 MH165180   TTGAATACAATTTCAAATTGTCTGTTAATTAGGTTAGCTTGGTTAGGTATTACTGATTTGCCTTTGTCCGAGTTCTCTCAAAATGTTGTTCTTGTTCGTT
DWV-NVN           TTGAATACAATTTCAAATTGTCTGTTAATTAGGTTAGCTTGGTTAGGTATTACTGATTTACCTTTATCCGAATTCTCTCAAAATGTTGTTCTCGTTTGTT
DWV-SVN           TTGAATACGATCTCAAATTGTCTGTTAATTAGGTTAGCTTGGTTAGGTATTACTGATTTACCTTTATCCGAGTTCTCTCAAAATGTTGTTCTTGTTTGTT

                          9410      9420      9430      9440      9450      9460      9470      9480      9490      9500
                  ....|....|....|....|....|....|....|....|....|....|....|....|....|....|....|....|....|....|....|
Japan AB070959    ATGGTGATGATCTTATCATGAATGTTAGTGATAACATGATTGATAAATTTAATGCTGTAACAATAGGGAAATTCTTTTCACAATATGAGATGGTATTTAC
USA1 AY291384     ATGGCGACGATCTTATCATGAATGTTAGCGATAACATGATTGATAAGTTTAACGCCGTGACGATAGGAAAATTCTTTTCACAATATAAGATGGAATTTAC
Chile JQ413340    ATGGCGATGATCTTATCATGAATGTTAGCGATAATATGATTGATAAGTTTAACGCCGTGACGATAGGAAAATTCTTTTCACAATATAAGATGGAATTTAC
Italy1 AJ489744   ATGGCGACGATCTTATCATGAATGTTAGYGATAACATGATTGATAAGTTTAATGCCGTGACGATAGGAAAATTCTTTTCACAATATAAGATGGAATTTAC
France KX373899   ATGGCGATGATCTTATCATGAATGTTAGTGATAACATGATTGATAAGTTTAATGCCGTGACGATAGGAAAATTCTTTTCACAATATAAGATGGAATTTAC
UK1 GU109335      ATGGTGATGATCTTATCATGAATGTTAGTGATAACATGATTGATAAATTTAATGCTGTGACAATAGGGAAATTCTTTTCACAATATAAGATGGAATTTAC
UK2 KJ437447      ATGGTGATGATCTTATCATGAATGTTAGTGATAACATGATTGATAAATTTAATGCTGTGACAATAGGGAAATTCTTTTCACAATATAAGATGGAATTTAC
Korea1 JX875304   ATGGTGATGATCTTATCATGAATGTTAGTGATAACATGATTGATAAATTTAATGCTGTGACGATAGGGAGATTCTTTTCACAATATAAGATGGAATTTAC
Korea2 JX878305   ATGGTGATGATCTTATCATGAATGTTAGTGATAACATGATTGATAAATTTAATGCTGTGACAATAGGGAAATTCTTTTCACAATATAAGATGGAATTTAC
China1 MF770715   ATGGTGATGATCTTATCATGAATGTTAGTGATAACATGATTGATAAATTTAATGCTGTGACAATAGGGAAATTCTTTTCACAATATAAGATGGAATTCAC
China2 MF036686   ATGGTGATGATCTTATCATGAATGTTAGTGATAACATGATTGATAAATTTAATGCTGTGACAATAGGGAAATTCTTTTTACAATATAAGATGGAATTTAC
China3 MH165180   ATGGTGATGATCTTATCATGAATGTTAGTGATAACATGATTGATAAATTCAACGCCGTGACAATAGGGAAATTCTTTTCACAATATAAGATGGAATTTAC
DWV-NVN           ACGGTGATGATCTTATCATGAACGTTAGTGATAATATGATTGATAAATTCAATGCTGTGACAATAGGGAAATTCTTTTCACAATATAAGATGGAATTTAC
DWV-SVN           ACGGTGATGATCTTATCATGAACGTTAGTGATAATGTGATTGATAAATTTAATGCTGTGACAATAGGGAAATTCTTTTCACAATATAAAATGGAATTTAC

                          9510      9520      9530      9540      9550      9560      9570      9580      9590      9600
                  ....|....|....|....|....|....|....|....|....|....|....|....|....|....|....|....|....|....|....|
Japan AB070959    GGATCAGGACAAATCAGGAAATACTGTGAAGTGGCGGACGTTACAGACTGCTACTTTCTTGAAGCATGGGTTTTTAAAACATCCAACTAGACCTGTGTTT
USA1 AY291384     GGATCAGGATAAATCAGGAAATACTGTAAAGTGGCGGACGTTACAGACTGCTACTTTCTTAAAACATGGGTTCTTAAAACATCCAACTAGACCTGTGTTT
Chile JQ413340    GGATCAGGATAAATCAGGAAATACTGTAAAGTGGCGGACGTTACAGACTGCTACTTTCTTAAAACATGGGTTCTTAAAACATCCAACTAGACCTGTGTTT
Italy1 AJ489744   GGATCAGGATAAATCAGGAAATACTGTAAAGTGGCGGACGTTACAGACTGCTACTTTCTTAAAACATGGGTTCTTAAAACATCCAACTAGACCTGTGTTT
France KX373899   GGATCAGGATAAATCAGGAAATACTGTAAAGTGGCGGACGTTACAGACTGCCACTTTCTTAAAACATGGGTTCTTAAAACATCCAACTAGACCTGTGTTT
UK1 GU109335      GGATCAGGACAAATCAGGAAATACTGTGAAGTGGCGGACGTTACAGACTGCTACTTTCTTGAAGCATGGGTTTTTAAAACATCCAACTAGACCTGTGTTT
UK2 KJ437447      GGATCAGGACAAATCAGGAAATACTGTGAAGTGGCGGACGTTACAGACTGCTACTTTCTTGAAGCATGGGTTTTTAAAACATCCAACTAGACCTGTGTTT
Korea1 JX875304   GGATCAGGACAAATCAGGAAATACAGTGAAGTGGCGGACGTTACAGACTGCTACTTTCTTGAAGCATGGGTTTTTAAAACATCCAACTCGACCTGTGTTT
Korea2 JX878305   GGATCAGGACAAATCAGGAAATACTGTGAAGTGGCGAACGTTACAGACTGCTACTTTCTTGAAGCATGGGTTTTTAAAACATCCAACTAGACCTGTGTTT
China1 MF770715   GGATCAGGACAAATCAGGAAATACTGTGAAGTGGCGGACGTTACAGACTGCTACTTTCTTGAAACATGGGTTTTTGAAACATCCAACTAGACCTGTGTTT
China2 MF036686   GGATCAGGACAAATCAGGAAATACAGTAAAGTGGCGGACGTTACAGACTGCTACTTTCTTGAAGCATGGGTTTTTAAAACATCCAACTAGACCTGTGTTT
China3 MH165180   GGATCAGGACAAGTCAGGAAATACTGTGAAGTGGCGGACCTTACAGACTGCTACTTTTTTGAAGCATGGGTTTTTAAAGCATCCAACTAGACCCGTGTTT
DWV-NVN           GGATCAGGATAAATCAGGAAATACTGTAAAGTGGCGGACGTTACAGACTGCCACTTTCTTGAAACACGGGTTTTTGAAACATCCAACTAGACCTGTGTTT
DWV-SVN           GGATCAGGATAAATCAGGAAATACTGTGAAGTGGCGGACGTTACAGACTGCTACTTTCTTGAAACACGGGTTTTTGAAACATCCAACTAGACCTGTGTTT
```

```
                              9610      9620      9630      9640      9650      9660      9670      9680      9690      9700
                     ....|....|....|....|....|....|....|....|....|....|....|....|....|....|....|....|....|....|....|....|
Japan AB070959       CTGGCTAACCTAGACAAGGTTTCGGTAGAAGGAACGACGAATTGGACCCATGCTCGAGGATTGGGTCGTCGTGCAGCAACCATAGAGAATGCTAAACAAG
USA1 AY291384        CTAGCTAACCTGGACAAGGTCTCGGTAGAAGGAACGACGAATTGGACTCATGCTCGAGGATTGGGTCGTCGTACAGCAACCATAGAAAATGCTAAACAAG
Chile JQ413340       CTAGCTAACCTGGACAAGGTCTCGGTAGAAGGAACGACGAATTGGACTCATGCTCGAGGATTGGGTCGTCGTACAGCAACCATAGAAAATGCTAAACAAG
Italy1 AJ489744      CTAGCTAACCTGGACAAGGTCTCGGTAGAAGGAACGACGAATTGGACTCATGCTCGAGGATTGGGTCGTCGTACAGCAACCATAGAAAATGCTAAACAAG
France KX373899      TTAGCTAACCTGGACAAGGTCTCGGTAGAAGGAACGACGAATTGGACTCATGCTCGAGGATTGGGTCGTCGTACAGCAACCATAGAAAATGCTAAACAAG
UK1 GU109335         CTGGCTAACCTAGACAAGGTTTCGGTAGAAGGAACGACGAATTGGACCCATGCTCGAGGATTGGGTCGTCGTACAGCAACCATAGAGAATGCTAAGCAAG
UK2 KJ437447         CTGGCTAACCTAGACAAGGTTTCGGTAGAAGGAACGACGAATTGGACCCATGCTCGAGGATTGGGTCGTCGTACAGCAACCATAGAAAATGCTAAACAAG
Korea1 JX875304      CTGGCTAACCTAGACAAGGTTTCGGTAGAAGGAACAACGAATTGGACCCATGCTCGAGGATTGGGTCGCCGTGCAGCAACCATAGAGAATGCTAAGCAAG
Korea2 JX878305      CTGGCTAACCTAGACAAGGTTTCGGTAGAAGGAACGACGAATTGGACTCATGCTCGAGGGTTGGGTCGTCGTGCAGCAACCATAGAGAATGCTAAACAAG
China1 MF770715      CTGGCTAACCTAGACAAGGTTTCGGTAGAAGGAACGACGAATTGGACCCATGCTCGAGGATTGGGTCGTCGTACAGCAACCATAGAGAATGCTAAACAAG
China2 MF036686      CTGGCCAACCTAGACAAGGTTTCGGTAGAAGGAACGACGAACTGGACCCATGCTCGAGGGTTGGGTCGTCGCGCAGCAACCATAGAGAATGCTAAACAAG
China3 MH165180      CTGGCTAACCTAGACAAGGTTTCGGTAGAAGGAACGACGAATTGGACCCATGCTCGAGGGTTGGGTCGTCGTACAGCAACCATAGAGAATGCTAAACAAG
DWV-NVN              CTAGCTAACCTGGACAAGGTGTCGGTAGAAGGAACGACGAACTGGACTCATGCTCGAGGATTGGGTCGTCGTGCAGCAACCATAGAGAATGCTAAACAAG
DWV-SVN              CTAGCTAACCTGGACAAGGTGTCGGTAGAAGGAACGACGAACTGGACTCATGCTCGAGGATTGGGTCGTCGTGCAGCAACCATAGAGAATGCTAAACAAG

                              9710      9720      9730      9740      9750      9760      9770      9780      9790      9800
                     ....|....|....|....|....|....|....|....|....|....|....|....|....|....|....|....|....|....|....|....|
Japan AB070959       CGCTAGAGTTAGCATTCGGATGGGGTCCAGAATACTTTAACTATGTCAGAAATACTATTAAAATGGCTTTTGACAAGTTGGGTATTTATGAAGACCTTAT
USA1 AY291384        CGTTAGAGTTAGCATTTGGGTGGGGTCCTGAATATTTTAATTATGTCAGAAATACCATTAAAATGGCTTTTGACAAGTTGGGTATTTATGAGGACCTTAT
Chile JQ413340       CGTTAGAGTTAGCATTTGGGTGGGGTCCTGAATATTTTAATTATGTCAGAAATACCATTAAAATGGCTTTTGACAAGTTGGGTATTTATGAGGACCTTAT
Italy1 AJ489744      CGTTAGAGTTAGCATTTGGGTGGGGTCCTGAATATTTTAATTATGTCAGAAATACCATTAAAATGGCTTTTGACAAGTTGGGTATTTATGAGGACCTTAT
France KX373899      CGTTAGAGTTAGCATTTGGATGGGGTCCTGAATATTTTAATTATGTCAGAGATACCATTAAAATGGCTTTTGACAAGTTGGGTATTTATGAGGACCTTAT
UK1 GU109335         CGCTAGAGTTAGCATTCGGATGGGGTCCAGAATACTTTAACTATGTCAGAAATACTATTAAAATGGCTTTTGACAAGTTGGGTATTTATGAAGACCTTAT
UK2 KJ437447         CGTTAGAGTTAGCATTTGGGTGGGGTCCTGAATATTTTAATTATGTCAGAAATACCATTAAAATGGCTTTTGACAAATTGGGTATTTATGAGGACCTTAT
Korea1 JX875304      CGTTAGAGTTAGCATTTGGGTGGGGTCCAGAATACTTTAACTATGTCAGAAGTACTATTAAAATGGCTTTTGATAAGTTGGGTATTTATGAAGACCTTAT
Korea2 JX878305      CGTTAGAGTTAGCATTCGGATGGGGTCCGGAATACTTTAACTATGTCAGAAGTACTATTAAAATGGCTTTTGACAAGTTGGGTATTTATGAAGACCTTAT
China1 MF770715      CGCTAGAGTTAGCGTTCGGATGGGGTCCAGAATACTTTAATTATGTCAGAAGTACTATTAAAATGGCTTTTGACAAGTTGGGTATTTATGAAGACCTTAT
China2 MF036686      CGCTAGAGTTAGCATTCGGATGGGGTCCAGAATACTTTAACTATGTCAGAAGTACTATTAAAATGGCTTTTGACAAGTTGGGTATTTATGAAGACCTCAT
China3 MH165180      CGCTAGAGTTAGCATTCGGATGGGGTCCAGAATACTTTAACTATGTCAGAAGTACTATTAAAATGGCTTTTGACAGGTTGGGTATTTATGAAGACCTTAT
DWV-NVN              CGCTAGAGTTAGCATTCGGATGGGGTCCCGAATACTTTAACTATGTCAGAAATACCATTAAAATGGCTTTTGACAAGTTGGGTATTTATGAAGACCTTAT
DWV-SVN              CGCTAGAGTTAGCATTCGGATGGGGTCCCGAATACTTTAACTATGTCAGAAATACCATTAAAATGGCTTTTGACAAGTTGGGTATTTATGAAGACCTTAT

                              9810      9820      9830      9840      9850      9860      9870      9880      9890      9900
                     ....|....|....|....|....|....|....|....|....|....|....|....|....|....|....|....|....|....|....|....|
Japan AB070959       CACATGGGAAGAAATGGATGTTAGATGTTATGCTAGCGCGTAGTATTTAATT-TTAAATACTTATTAGTTTTAATTTTATTTT-AGGTTATTGGAATTGA
USA1 AY291384        CACATGGGAAGAAATGGATGTTAGATGTTACGCTAGCGCGTAATATTAAATT-TTAAATACTCATTAATTTTAATTTTATTTT-AGGTTATTGGAATTGA
Chile JQ413340       CACATGGGAAGAAATGGATGCTAGATGTTACGCTAGCACGTAATATTAAATT-TTAAATACTCATTAATTTTAATTTTATTTT-AGGTTATTGGAATTGA
Italy1 AJ489744      CACATGGGAAGAAATGGATGTTAGATGTTACGCTAGTGCGTAATATTAAATT-TTAAATACTCATTAATTTTAATTTTATTTT-AGGTTATTGGAATTGA
France KX373899      CACATGGGAAGAAATGGATGTTAGATGTTACGCTAACGCGTAATATTAAATT-TTAAATACTCATTAATTTTAATTTTATTTT-AGGTTATTGGAATTGA
UK1 GU109335         CACATGGGAAGAAATGGATGTTAGATGTTATGCTAGCGCGTAGTATTTAATT-TTGAATACTTATTAGTTTTAATTTTATTTT-AGGTTATTGGAATTGA
UK2 KJ437447         CACATGGGAAGAAATGGATGTTAGATGTTACGCTAGCGCGTAATATTGAATT-TTAAATATTCATTAATTTTAATTTTATTTT-AGGTTATTGGAATTGA
Korea1 JX875304      CACATGGGAGGAAATGGATGTTAGATGTTATGCTAGCGCGTAGTATTTAATT-TTGAATACTCATTAGTTTTAATTTTATTTT-AGGTTATTGGAATTGA
Korea2 JX878305      CACATGGGAAGAAATGGATGTTAGATGTTATGCTAGCGCGTAGTATTTAATT-TTGAATACTCATTAGTTTTAATGTTATTTT-AGGTTATTGGAATTGA
China1 MF770715      CACATGGGAAGAGATGGATGTTAGATGTTATGCTAGCGCGTAGTATTTAATT-GTAAATACTTATTAGTTTTAATTTTATTTT-AGGTTATTGGAATTGA
China2 MF036686      CACATGGGAAGAAATGGATGTTAGATGTTATGCTAGCGCGTAGTATTTAATT-TTGAATATTTATTAGTTTTAATTTTATTTT-AGGTTATTGGAATTGA
China3 MH165180      CACATGGGAAGAAATGGATGTTAGATGTTATGCTAGCGCGTAGTATTTGATTTATGAATACTGATTAGTTTTAATTTTATTTTTAGGTTATTGGAATTGA
DWV-NVN              CACATGGGAAGAAATGGATGTTAGATGTTATGCTAGCGCGTAGTATTTAATTTCTAAATACTCATTAGTTTTAATTTTTATTTTAGGTTATTGGAATTGA
DWV-SVN              CACATGGGAAGAAATGGATGTTAGATGTTATGCTAGCGCGTAGTATTTAATTTCTAAATACTCATTAGTTTTAATTTTTATTTTAGGTTATTGGAATTGA

                              9910      9920      9930      9940      9950      9960      9970      9980      9990     10000
                     ....|....|....|....|....|....|....|....|....|....|....|....|....|....|....|....|....|....|....|....|
Japan AB070959       GGGAAGTACCACCCCCCAAGACCTTCGTTTTAAATCTACTAAAAGGAGTGGACCTATATATAAGAGCCTGAAGACAGAGTGGATTAGACCACCATTTTTA
USA1 AY291384        GGGAAGTACCACCCCCCAAGACCTTCGTTTTAAATCTACTAAGAGGAGTAAACCTATATATAAGAGTCTAAAGACAGAGTGGATTAGACCATCATCTTTA
Chile JQ413340       GGGAAGTACCACCCCCCAAGACCTTCGTTTTAAATCTACTAAGAGGAGTAAACCTATATATAAGAGTCTAAAGACAGAGTGGATTAGACCATCATCTTTA
Italy1 AJ489744      GGGAAGTACCACCCCCCAAGACCTTCGTTTTAAATCTACTAAGAGGAGTAAACCTATATATAAGAGTCTAAAGACAGAGTGGATTAGACCATCATCTTTA
France KX373899      GGGAAGTACCACCCCCCAAGACCTTCGTTTTAAATCTACTAAGAGGAGTAAACCTATATATAAGAGTCTAAAGACAGAGTGGATTAGACCATCATCTTTA
UK1 GU109335         GGGAAGTACCACCCCCCAAGACCTTCGTTTTAAATCTACTAAAAGGAGTGAACCTATATATAAGAGTCTAACGACAGAGTGGATCAGACCACCATCTTTA
UK2 KJ437447         GGGAAGTACCACCCCCCAAGACCTTCGTTTTAAATCTACTAAGAGGAGTAAACCTATATATAAGAGTCTAAAGACAGAGTGGATTAGACCATCATCTTTA
Korea1 JX875304      GGGAAGTACCACCCCCCAAGACCTTCGTTTTAAATCTACTAAAAGGAGTGAACCTATATATAAGAGTCTAAAGACAGAGTGGATTAGACCACCATTTTTA
Korea2 JX878305      GGGAAGTACCACCCCCCAAGACCTTCGTTTTAAATCTACTAAAAGGAGTGAACCTATATATAAGAGTCTAAAGACAGAGTGGATTAGACCACCATTTTTA
China1 MF770715      GGGAAGTACCACCCCCCGAGACCTTCGTTATAAGTCTACTAAAAGGAGTGAACCTATATGTAAGAGTCTAAAGACAGAGTGGATTAGACCACCATTTTTA
China2 MF036686      GGGAAGTACCACCCCCCAAGACCTTCGTTTTAAATCTACTAAAAGGAGTGAACCTATATATAAGAGTCTAAAGACAGAGTGGAATAGACCACCATTT---
China3 MH165180      GGGAAGTACCACCCCCCAAGACCTTCGTTTTAAATCTACTAAAAGGAGTGAACCTGTATATAAGAGTCTAAAGACAGAGTGGATTAGACCACCATTTTTA
DWV-NVN              GGGAAGTACCACCCCCCAAGACCTTCGTTTTAAATCTACTAAGAGGAGTGAACCTATATATAAGAGTCCAAAGACAGAGTGGATTAGACCACCATCTTTG
DWV-SVN              GGGAAGTACCACCCCCCAAGACCTTCGTTTTAAATCTACTAAGAGGAGTGAACCTATATATAAGAGTCCAAAGACAGAGTGGATTAGACCACCATCTTTG
```

```
                        10010     10020     10030     10040     10050     10060     10070     10080     10090     10100
                ....|....|....|....|....|....|....|....|....|....|....|....|....|....|....|....|....|....|....|....|
Japan AB070959  GCTTATATATGGGAAAGGTTGAGTTGCCTCTAAAGACTCAGCTCCATAGTAGAGTAGTTTTAATTACGATTAAAGTGGTACTCTAGGTTAGGTGTTACTC
USA1 AY291384   GCTTATATATGGGGAAGGTTGAGTTGCCTCTAAAGACTCAGCTCCATAGTAGAGTAGTTTTAATTACGATTAAAGTGGTACTCTAGGTTAGGTGTTACTC
Chile JQ413340  GCTTATATATGGGGAAGGTTGAGTTGCCTCTAAAGACTCAGCTCCATAGTAGAGTAGTTTTAATTACGATTAAAGTGGTACTCTAGATTAGGTGTTACTC
Italy1 AJ489744 GCTTATATACGGGGAAGGTTGAGTTGCCTCTAAAGACTCAGCTCCATAGTAGAGTAGTTTTAATTACGATTAAAGTGGTACTCTAGGTTAGGTGTTACTC
France KX373899 GCTTATATACGGGGAAGGGTGAGTTGCCTCTAAAGACTCAGCTCCATAGTAGAGTAGTTTTAATTACGATTAAAGTGGTACTCTAGGTTAGGTGTTACTC
UK1 GU109335    GCTTATATATGGGAAAGGTTGAGTTGCCTCTAAAGACTCAGCTCCATAGTAGAGTAGTTTTAATTACGATTAAAGTGGTACTCTAGGTTAGGTGTTACTC
UK2 KJ437447    GCTTATATATGGGGAAGGTTGAGTTGCCTCTAAAGACTCAGCTCCATAGTAGAGTAGTTTTAATTACGATTAAAGTGGTACTCTAAGTTAGGTGTTACTC
Korea1 JX875304 GCTTATATATGGGAAAGGTTGAGTTGCCTCTAAAGACTCACCTCCATAGTATATTAGTTTTAATTACCATTAACGTGGTACTCTAGATTAGGTGTTACTC
Korea2 JX878305 GCTTATATATGGGAAAGGTTGAGTTGCCTCTAAAGACTCAGCTCCATAGTAGAGTAGTTTTAATTACGATTAAAGTGGTACTCTAGGTTAGGTGTTACTC
China1 MF770715 GCTTATATATGGGAAAGGTTGAGTTGCCTCTAAAGACTCAGCTCCATAGTAGAGTAGGTTTAATTACGATTAAAGTGGTACTCTAGGTTAGGTGTTACTC
China2 MF036686 ----------------------------------------------------------------------------------------------------
China3 MH165180 GCTTATATATGGGAAAGGTTGAGTTGCCTCTAAAGACTCAGCTCCATAGTAGAGTAGTTTTAATTACGATTAAAGTGGTACTCTAGGTTAGGTGTTACTC
DWV-NVN         GCTTATATATGGGACAGGTTGAGTTGCCTCTAAAGACTCAGCTCCATAGTAGAGTAGTTTTAATTACGATTAAAGTGGTACTCTAGGTTAGGTGTTACTC
DWV-SVN         GCTTATATATGGGACAGGTTGAGTTGCCTCTAAAGACTCAGCTCCATAGTAGAGTAGTTTTAATTACGATTAAAGTGGTACTCTAGGTTAGGTGTTACTC

                        10110     10120     10130     10140     10150     10160     10170     10180     10190
                ....|....|....|....|....|....|....|....|....|....|....|....|....|....|....|....|....|....|
Japan AB070959  GCGTATTATCAACTAGTGGTAATGCGTCCTAATTTTAGTATAGTTTTAACCATAATAGT-------------------------------
USA1 AY291384   GCGTATTATCAACTAGTGGTAATGCGTCCTAATTTTAGTATAGTTTTAACCATAATAGTAAAAAAAAAAAAAAAAAAAAAAAAAAAAAAA
Chile JQ413340  GCGTATTATCAACTAGTGGTAATGCGTCCTAATTTTAGTATAGTTTTAACCATAATAGTAAAAAAAAAAAAAAAAAAAAAAAAAAAAAAA
Italy1 AJ489744 GCGTATTATCAACTAGTGGTAATGCGTCCTAATTTTAGTATAGTTTTAACCATAATAGT-------------------------------
France KX373899 GCGTATTATCAATTAGTGGTAATGCGTCCTAATTTTAGTATAGTTTTAACCATAATAGCAAAAAA-------------------------
UK1 GU109335    GCGTATTATCAACTAGTGGTAATGCGTCCTAATTTTAGTATAGTTTTAACCATAATAGTAAAAAAAAAAAAAAAAAAAAAAAAAAA----
UK2 KJ437447    GCGTATTATCAACTAGTGGTAATGCGTCCTAATTTTAGTATAGTTTTAACCATAATAGTAAAAAAAAAAAAAAAAAAAAAAAAAAA----
Korea1 JX875304 GCGTATTATCAACTAGTGGTAATGCGTCCTAAAAAAAAAAAAAAAAA-------------------------------------------
Korea2 JX878305 GCGTATTATCAACTAGTGGTAATGCGTCCTAAAAAAAAAAAAAAAAAA------------------------------------------
China1 MF770715 GCGTATTATCAACTAGTGGTAATGCGTCCTAATTTTAGTATAGTTTTAACCATAATAGTAAAAAAAAAAAAAAAAAAAAAAAAA------
China2 MF036686 ------------------------------------------------------------------------------------------
China3 MH165180 GCGTATTATCAACTAGTGGTAATGCGTCCTAA----------------------------------------------------------
DWV-NVN         GCGTATTATCAACTAGTGGTAATGCGTCCTAATCTTAGTAAGTA----------------------------------------------
DWV-SVN         GCGTATTATCAACTAGTGGTAATGCGTCCTAATCTTAGTAAGTA----------------------------------------------
```
