# Supplementary material for: Genome analysis and phylogenetic characterization of two deformed wing virus strains from Apis cerana in Vietnam
Source: PeerJ. 2020 Sep 21;8:e9911. doi: 10.7717/peerj.9911 (PMC7513742; doi:10.7717/peerj.9911)
Supplement: Supplemental Information 3 [file peerj-08-9911-s003.pdf]

Table S1. Nucleotide sequences and amino acid sequences for the coding region homology (%) between DWV-VN and the other reference sequences.

	Complete nucleotide sequences (%)		Nucleotide sequence for 5'UTR region (%)		Nucleotide sequence for 3'UTR region (%)		Amino acid sequences (%)	
	DWV-NVN MN607197	DWV-SVN MN607198	DWV-NVN MN607197	DWV-SVN MN607198	DWV-NVN MN607197	DWV-SVN MN607198	DWV-NVN MN607197	DWV-SVN MN607198
USA1 AY292384	92.0	91.7	94.6	95.1	82.6	82.6	97.9	97.7
Chile JQ413340	91.7	91.4	94.4	94.9	82.3	82.3	97.7	97.6
Italy1 AJ489744	91.8	91.6	94.0	94.4	90.1	90.1	97.7	97.5
France KX373899	91.8	91.6	94.2	94.7	87.8	87.8	97.6	97.4
UK1 GU109335	91.8	91.7	93.7	94.1	90.1	90.1	97.8	97.7
UK2 KJ437447	89.4	89.2	93.7	94.1	83.3	83.3	97.4	97.2
Japan AB070959	91.9	91.7	93.2	93.6	82.7	82.7	97.6	97.5
Korea1 JX878304	91.7	91.4	93.1	93.5	90.7	90.7	96.8	96.7
Korea2 JX878305	91.9	91.8	93.8	94.3	92.1	92.1	97.4	97.3
China1 MF770715	91.6	91.4	93.9	94.4	82.7	82.7	97.1	97.1
China2 MF036686	89.6	89.6	82.8	83.1	NA	NA	97.6	97.6
China3 MH165180	91.9	91.7	94.0	94.8	92.0	92.0	97.0	97.0
